# Real-World Evaluation Study of Azvudine for the Treatment of Patients With COVID-19: A Systematic Review and Meta-Analysis

**DOI:** 10.1155/cjid/3645253

**Published:** 2025-09-26

**Authors:** Abiden Kapar, Huling Li, Qian He, Dandan Lin, Dandan Tang, Kai Peng, Yida Wang, Kai Wang

**Affiliations:** ^1^School of Biomedical Engineering, Hainan University, Sanya, China; ^2^School of Public Health, Xinjiang Medical University, Urumqi, China; ^3^Department of Medical Engineering and Technology, Xinjiang Medical University, Urumqi, China

**Keywords:** Azvudine, COVID-19, meta-analysis, SARS-CoV-2

## Abstract

**Background:** Azvudine, as an antiviral drug, has been approved for the treatment of COVID-19, and multiple randomized controlled trials (RCTs) and retrospective cohort studies have been conducted. This study aimed to systematically evaluate the efficacy and safety of Azvudine in treating COVID-19 patients.

**Methods:** As of December 1, 2023, we searched databases including PubMed, Web of Science, Ovid, ICTRP, Cochrane Library, Clinical Trials, MedRxiv, and Springer Link for relevant RCTs and retrospective cohort studies. EndNote X9 was used for literature screening and management, and R software was employed for meta-analysis.

**Results:** A total of 1142 COVID-19 patients from five RCTs were included, with 575 patients receiving Azvudine treatment. Azvudine significantly reduced the hospitalization time and the time to nucleic acid conversion to negative in patients with mild to moderate COVID-19. However, compared to the control group, Azvudine did not significantly reduce the incidence of adverse events (AEs) (risk ratio: 0.89, 95% confidence interval [CI]: 0.80, 1.00). Additionally, eight ongoing clinical trials were included to evaluate the efficacy and safety of Azvudine. In fourteen retrospective cohort studies, a total of 6602 COVID-19 patients were analyzed, with 3118 patients receiving Azvudine treatment. Azvudine significantly reduced all-cause mortality (odds ratio [OR]: 0.49, 95% CI: 0.38, 0.63). The incidence of AEs in the Azvudine group and the Nirmatrelvir/Ritonavir group was 4.13% (60/1453) and 5.08% (67/1319), respectively, indicating that Azvudine significantly reduced the incidence of AEs compared to Nirmatrelvir/Ritonavir (OR: 0.68, 95% CI: 0.47, 0.98).

**Conclusions:** Azvudine significantly reduced the hospitalization time and the time to nucleic acid conversion to negative in COVID-19 patients and significantly lowered all-cause mortality (Grading of Recommendations Assessment, Development, and Evaluation [GRADE]: high-certainty evidence). In terms of safety, Azvudine demonstrated a favorable safety profile (GRADE: moderate-certainty evidence because of suspected publication bias and residual confounding). Further large-scale studies are needed to validate its efficacy and safety.

## 1. Introduction

Since the outbreak of Coronavirus Disease 2019 (COVID-19) at the end of 2019, it has caused hundreds of millions of infections and millions of deaths worldwide [1, 2]. This pandemic is the most severe public health event humanity has faced in nearly a century, posing a significant threat to human life and health [3, 4]. COVID-19 is primarily transmitted through respiratory routes, and due to its high infectivity, rapid spread, and diverse transmission pathways, it can lead to severe respiratory diseases and even life-threatening conditions [5]. In the early stages of the pandemic, some broad-spectrum antiviral drugs and drugs targeting other specific viruses, such as Molnupiravir [6], Favipiravir [7], and Paxlovid (Nirmatrelvir/Ritonavir) [8], played important roles in combating the epidemic. However, there is still a lack of specific drugs for treating COVID-19 infections, making the search for effective treatments crucial for controlling the pandemic and improving patient outcomes.

Azvudine is a nucleoside reverse transcriptase inhibitor that has shown efficacy in treating HIV and HBV infections [9, 10]. It is the first dual-target antiviral drug that inhibits both the auxiliary protein and reverse transcriptase of HIV [11]. Azvudine has also demonstrated significant effects in treating COVID-19 patients. After entering host cells, Azvudine is phosphorylated by kinases into its active form, which then binds to the RNA-dependent RNA polymerase (RdRp) of COVID-19, leading to the termination of viral RNA synthesis and inhibition of viral replication [12–14]. Additionally, Azvudine has been shown to inhibit the RdRp of the virus [15, 16]. Overall, Azvudine suppresses COVID-19 by interfering with viral RNA synthesis and replication. On July 25, 2022, the National Medical Products Administration of China conditionally approved Azvudine tablets (1 mg/tablet) for the treatment of adult patients with moderate COVID-19 [17, 18]. Subsequently, the National Health Commission and the State Administration of Traditional Chinese Medicine included Azvudine in the COVID-19 treatment guidelines. On January 6, 2023, Azvudine was officially included in the treatment of COVID-19, and in February 2023, the Russian Ministry of Health approved its use for COVID-19 patients [19].

However, with the widespread use of Azvudine, instances of inappropriate use and adverse events (AEs) have been reported. Although Azvudine is considered a safe and effective treatment for adult patients with moderate COVID-19 and has good patient compliance [20], more research and clinical trials are needed to confirm its efficacy and safety. Currently, there is a lack of meta-analyses on the efficacy and safety of Azvudine in treating COVID-19. Therefore, to provide more reliable evidence for the clinical application of Azvudine in COVID-19 treatment, this study will conduct a systematic review and meta-analysis of its therapeutic effects and safety.

## 2. Methods

### 2.1. Eligibility Criteria

The inclusion criteria were: (1) Study population includes patients aged 18 years and older with varying severity of COVID-19 (mild, moderate, severe, and critical). (2) Patients receiving Azvudine as an antiviral treatment during hospitalization were included in the intervention group, while those receiving placebo or standard treatment (including antiviral and symptomatic treatments) were included in the control group. (3) Both randomized controlled trials (RCTs) and cohort studies were included, and studies without control groups (single-arm trials), studies without data, and related meta-analyses were excluded during the screening phase to ensure the reliability and scientific rigor of this study. (4) All studies included in this review were required to be registered on international clinical trial registration platforms (ICTRP), ClinicalTrials.gov, or the Chinese Clinical Trial Registry (https://www.chictr.org.cn/) or approved by institutional ethics review boards.

### 2.2. Search Strategy and Literature Screening Criteria

The search terms used in this study were “COVID-19” or “SARS-CoV-2” and “Azvudine” or “FNC” (Appendix 1). The initial search was conducted on December 1, 2023, across databases including PubMed, Web of Science, Ovid, ICTRP, Cochrane Library, Clinical Trials, MedRxiv, and Springer Link. To ensure no relevant studies were missed, a second search using the same terms was conducted on January 20, 2024.

To avoid overlooking any potentially relevant studies, we also reviewed the reference lists of the selected articles and used EndNote X9 to filter out duplicate records. Subsequently, we read the titles and abstracts of the relevant articles and performed an initial screening. For articles that met the preliminary inclusion criteria, we further reviewed the full text to determine their eligibility for inclusion. This process helped ensure that we included all reliable and relevant literature while minimizing potential bias.

### 2.3. Data Extraction

1. For RCTs, the following information was extracted: author, publication year, country, clinical trial registration number, phase, center, study population, gender distribution, median age, intervention and control group drug names, and sample size. We also extracted information from ongoing clinical trials, including registration date, clinical trial registration number, trial status, phase, center, study type, age range of participants, and intervention and control group drug names and sample sizes. We carefully reviewed the full text and recorded AEs and serious adverse events (SAEs) during hospitalization.
2. For retrospective cohort studies, the following information was extracted: author, publication year, country, patient enrollment period, center, gender distribution, median age, and intervention and control group drug names and sample sizes. We carefully reviewed the full text and recorded all-cause mortality and composite disease progression outcomes (e.g., ICU admission, invasive mechanical ventilation).

### 2.4. Risk of Bias Assessment and Certainty of Evidence

The quality of RCTs was assessed using the Cochrane Risk of Bias tool, while the quality of cohort studies was evaluated using the Newcastle–Ottawa Scale (NOS). Additionally, the certainty of evidence was assessed using the Grading of Recommendations Assessment, Development, and Evaluation (GRADE) tool, which considers domains such as risk of bias, inconsistency, indirectness, and imprecision to evaluate the level of certainty in the evidence.

### 2.5. Statistical Analysis

All data processing and analysis were performed using R statistical software (Version 4.3.0). The “meta” and “randomForest” packages were used for data analysis and visualization. For RCTs, we calculated the risk ratio (RR) with a 95% confidence interval (CI) to assess outcomes, whereas for retrospective cohort studies, we used the odds ratio (OR) with a 95% CI. Heterogeneity between studies was assessed using the *I*^2^ test. If *p* > 0.05 and *I*^2^ < 50%, a fixed-effects model was used; otherwise, a random-effects model was applied.

## 3. Results

**Figure 1:**
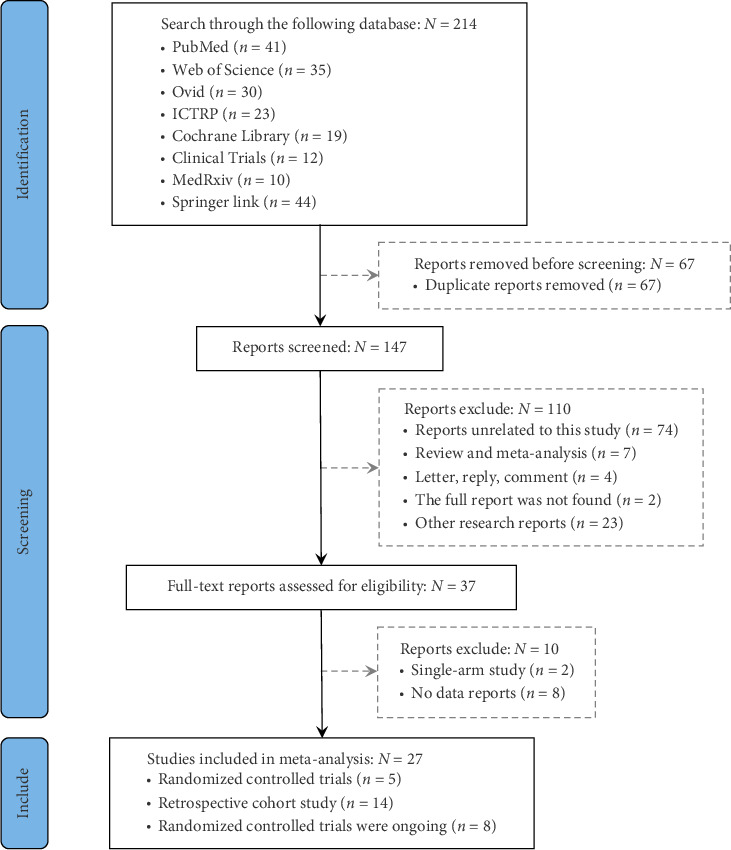
Flowchart of study selection.

A total of 214 articles were retrieved from 8 databases, including 41 from PubMed, 35 from Web of Science, 30 from Ovid, 23 from ICTRP, 19 from Cochrane Library, 12 from Clinical Trials, 10 from MedRxiv, and 44 from Springer Link. After removing 67 duplicate records, 74 articles were excluded based on title and abstract screening for being irrelevant to the study topic, 7 were excluded as systematic reviews or meta-analyses, 8 were excluded due to lack of relevant data, and other studies (e.g., in vitro studies, letters, single-arm trials, and studies with unavailable full text) were also excluded. The detailed screening process is shown in the PRISMA flowchart (Figure 1). After thorough screening, five RCTs [21–24] (Table 1), eight ongoing clinical trials (Table 2), and fourteen retrospective cohort studies [25–38] (Table 3) were included.

### 3.1. RCTs

The five RCTs [21–24] included a total of 1142 participants, with 575 patients randomized to the Azvudine group and 567 to the control group. Participants in the intervention group (FNC group) received 5 mg of Azvudine tablets plus standard treatment, while the control group received placebo or Azvudine dummy tablets plus standard treatment. These five Phase III clinical trials were conducted in China [21, 22] (two studies), Brazil [23, 24] (two studies), and Russia [22] (one study), with two being multicenter studies. Table 1 summarizes the basic information of the included RCTs, Table 4 presents their outcome metrics, and Table 2 presents the eight ongoing clinical trials.

#### 3.1.1. Efficacy of Azvudine

In the two RCTs conducted in China, one single-center study [21] showed that the time to first nucleic acid conversion to negative was 2.6 days in the Azvudine group, significantly shorter than the 5.6 days in the control group (*p*=0.008), indicating that Azvudine significantly reduced the time to nucleic acid conversion to negative. Another multicenter study [22] found no significant difference in viral load (primary outcome) and time to nucleic acid conversion to negative (secondary outcome) between the Azvudine and control groups. In the two RCTs conducted in Brazil, one study [23] involving moderate COVID-19 patients showed that the time to first nucleic acid conversion to negative was 6.24 days in the Azvudine group, significantly shorter than the 7.94 days in the control group (*p*=0.002); the hospitalization time was 6.5 days and 8 days, respectively (*p*=0.028), indicating that Azvudine significantly reduced both the time to nucleic acid conversion to negative and hospitalization time in moderate COVID-19 patients. Another multicenter study [24] involving mild COVID-19 patients showed that the time to first nucleic acid conversion to negative was 5.55 days in the Azvudine group, significantly shorter than the 8.27 days in the control group (*p* < 0.001). In the study conducted in Russia [22], the proportion of patients with clinical improvement on day 7 of Azvudine treatment was significantly higher in the Azvudine group than in the control group (36.31% vs. 9.55%, *p* < 0.001), and the median time to clinical improvement was significantly shorter (10 days vs. 13 days, *p* < 0.001). Additionally, the viral load in the Azvudine group was reduced [22–24] (Table 4).

#### 3.1.2. Safety of Azvudine

**Figure 2:**
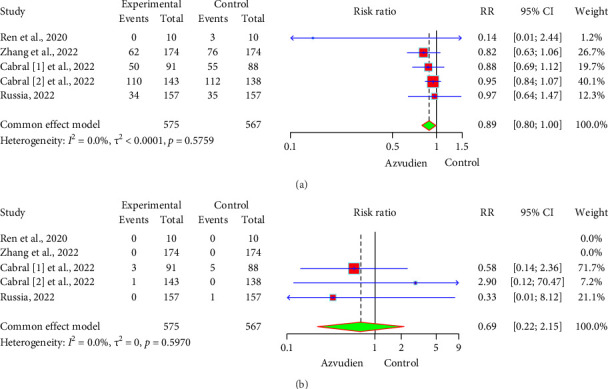
Forest plot of adverse events and serious adverse events in the Azvudine group and control group. (a) Adverse events. (b) Serious adverse events.

In the comparison of AEs between the Azvudine and control groups, the forest plot (Figure 2(a)) showed that the incidence of AEs was 44.52% (256/575) in the Azvudine group and 49.56% (281/567) in the control group. The RR between the two groups was 0.89 (95% CI: 0.80, 1.00), indicating that the incidence of AEs in the Azvudine group was 11% lower than in the control group. However, due to the wide CI, this result was not statistically significant (*p* > 0.05). In the comparison of SAEs between the Azvudine and control groups, the forest plot (Figure 2(b)) showed that the incidence of SAEs was 0.70% (4/575) in the Azvudine group and 1.06% (6/567) in the control group. The RR between the two groups was 0.69 (95% CI: 0.22, 2.15), indicating that the incidence of SAEs in the Azvudine group was 31% lower than in the control group. However, due to the wide CI, this result was not statistically significant (*p* > 0.05). Heterogeneity testing showed low heterogeneity between studies (*I*^2^ = 0.0%, *p* > 0.05), indicating high consistency among the study results.

#### 3.1.3. Subgroup and Sensitivity Analyses

The subgroup analysis results indicated that there were no significant differences in the RR of AEs and SAEs between different countries and levels of disease severity (Table 5). The sensitivity analysis results indicated that the heterogeneity among studies was low (*I*^2^ = 0.0%, *p* > 0.05), suggesting a high level of consistency in the findings. Through the “leave-one-out” approach, the estimated RR remained stable, further confirming the robustness of the primary analysis results (Figures S1–S10).

#### 3.1.4. Risk of Bias and Certainty of Evidence

The certainty of evidence for AEs was moderate, with an RR of 0.89 (95% CI: 0.80, 1.00), indicating that the incidence of AEs in the Azvudine group was slightly lower than that in the control group. However, the results demonstrated some imprecision, primarily due to the single-center design and small sample size. Similarly, the certainty of evidence for SAEs was also moderate, with an RR of 0.69 (95% CI: 0.22, 2.15), suggesting a potential reduction in the incidence of SAEs in the Azvudine group compared to the control group. Nevertheless, the wide CI indicated a higher degree of imprecision in these results (Table 6). The quality assessment of the included studies is presented in Table S1.

#### 3.1.5. Ongoing RCTs

In the eight RCTs, a total of 4148 participants were included. These trials cover Phase II, III, and IV clinical trials, with study designs including single-center and multicenter studies. The control groups were divided into placebo, traditional treatment, and antiviral treatment groups (e.g., Monotamivir, Paxlovid) based on the intervention (Table 2).

### 3.2. Cohort Study

The 14 retrospective cohort studies [25–38] included a total of 6602 participants, with 3118 patients in the Azvudine group and 3484 in the control group. These studies included 9 single-center [25–28, 31, 32, 35, 37, 38] and 5 multicenter studies [29, 30, 33, 34, 36], and all participants were over 18 years old. Detailed information is provided in Table 2. Supporting Table 2 presents the quality assessment of the included studies using the NOS. The symptoms of patients in the Azvudine group at admission included mild (252 cases), moderate (640 cases), severe (918 cases), and critical (186 cases); the symptoms of patients in the control group at admission were mild (524 cases), moderate (484 cases), severe (982 cases), and critical (168 cases) (Table S3). The study found that most COVID-19 patients had comorbidities, with the most common being heart disease, hypertension, and diabetes (Figure S72). Table 3 summarizes the basic information of the included retrospective cohort studies, while Table 7 presents their outcome metrics.

#### 3.2.1. Efficacy of Azvudine

In the cohort studies comparing Azvudine and Nirmatrelvir/Ritonavir [25–29], two studies [25, 28] showed no significant difference in hospitalization time between the two groups. Additionally, two other studies [26, 29] showed no significant difference in composite disease progression outcomes (including ICU admission, invasive mechanical ventilation, and in-hospital mortality) between the two groups. Deng [27] et al.'s study showed that Azvudine was associated with a significant reduction in the risk of composite disease progression outcomes (including all-cause mortality, ICU admission, invasive mechanical ventilation, and oxygen therapy) (HR: 0.55, 95% CI: 0.32, 0.94, *p*=0.029); however, in the cohort studies comparing Azvudine and the control group [30–38], four studies [33–35, 38] showed a significant reduction in mortality in patients taking Azvudine, while two other studies [31, 37] showed a significant reduction in the risk of composite disease progression outcomes in COVID-19 patients (*p* < 0.05). One study [36] showed that Azvudine significantly reduced the time to nucleic acid conversion and had significant therapeutic effects on asymptomatic and mild-to-moderate patients.

**Figure 3:**
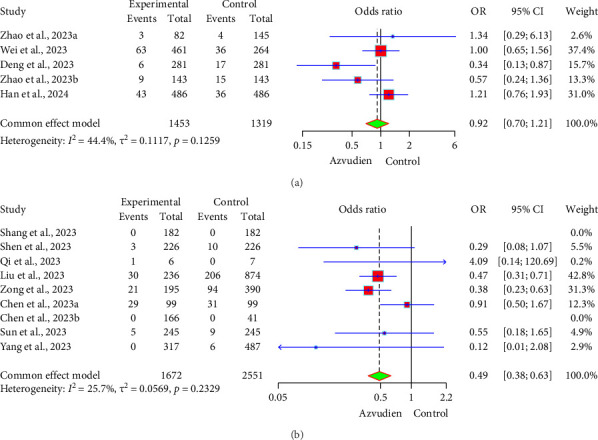
Forest plot of all-cause mortality in the Azvudine group and controls. (a) Azvudine and Nirmatrelvir/Ritonavir. (b) Azvudine and the control group.

In the comparison of all-cause mortality between Azvudine and Nirmatrelvir/Ritonavir, the forest plot (Figure 3(a)) shows that the incidence of all-cause mortality in the Azvudine and Nirmatrelvir/Ritonavir groups was 8.53% (124/1453) and 8.19% (108/1319), respectively. The OR between the two groups was 0.92 (95% CI: 0.70, 1.21), indicating no significant difference in all-cause mortality between the Azvudine and Nirmatrelvir/Ritonavir groups. In the comparison of all-cause mortality between Azvudine and the control group, the forest plot (Figure 3(b)) shows that the incidence of all-cause mortality in the Azvudine and control groups was 5.32% (89/1672) and 13.95% (356/2551), respectively. The OR between the two groups was 0.49 (95% CI: 0.38, 0.63), indicating that the incidence of all-cause mortality in the Azvudine group was 51% lower than in the control group, and this result was statistically significant (*p* < 0.05). Heterogeneity tests showed low heterogeneity between studies (*I*^2^ = 25.7%, *p* > 0.05), indicating high consistency among the study results.

**Figure 4:**
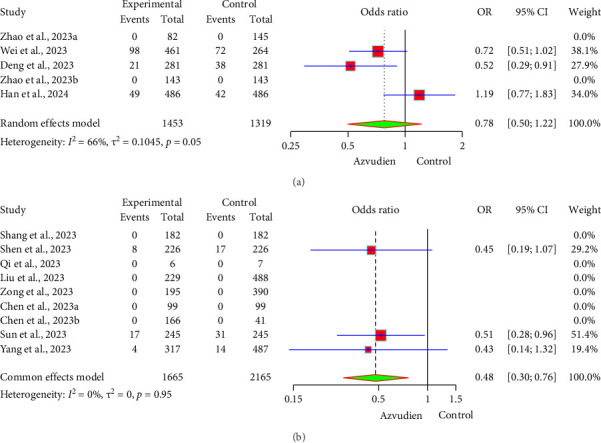
Forest plot of composite disease progression of the Azvudine group and controls. (a) Azvudine and Nirmatrelvir/Ritonavir. (b) Azvudine and the control group.

In the comparison of composite disease progression outcomes between Azvudine and Nirmatrelvir/Ritonavir, the forest plot (Figure 4(a)) shows that the incidence of composite disease progression outcomes in the Azvudine and Nirmatrelvir/Ritonavir groups was 11.56% (168/1453) and 11.52% (152/1319), respectively. The OR between the two groups was 0.78 (95% CI: 0.50, 1.22), indicating that the incidence of composite disease progression outcomes in the Azvudine group was 22% lower than in the Nirmatrelvir/Ritonavir group. However, this result was not statistically significant (*p* > 0.05). The forest plot (Figure 4(b)) shows that the incidence of composite disease progression outcomes was 1.74% (29/1665) in the Azvudine group compared to 2.86% (62/2165) in the control group, with an OR of 0.48 (95% CI: 0.30, 0.76). This indicates that the incidence of composite disease progression outcomes in the Azvudine group was 52% lower than in the control group, and this result was statistically significant (*p* < 0.05). Heterogeneity tests showed low heterogeneity between studies (*I*^2^ = 0.0%, *p* > 0.05), indicating high consistency among the study results.

#### 3.2.2. Safety of Azvudine

**Figure 5:**
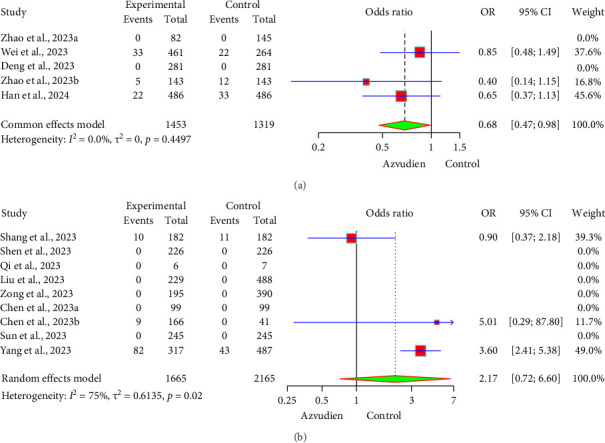
Forest plot of adverse events in the Azvudine group and control group. (a) Azvudine and Nirmatrelvir/Ritonavir. (b) Azvudine and the control group.

One study [30] showed that 10 patients in the Azvudine group experienced AEs, while 11 AEs occurred in the control group receiving basic treatment. There was no significant difference between the two groups, and no significant changes in liver or kidney function were observed. Three studies [26, 28, 29] found a significant difference in AEs between the Azvudine and Nirmatrelvir/Ritonavir groups. Importantly, no drug-related AEs or liver/kidney organ damage were reported in either group. The forest plot (Figure 5(a)) shows that the incidence of AEs in the Azvudine and Nirmatrelvir/Ritonavir groups was 4.13% (60/1453) and 5.08% (67/1319), respectively. The OR between the two groups was 0.68 (95% CI: 0.47, 0.98), indicating that the incidence of AEs in the Azvudine group was 32% lower than in the Nirmatrelvir/Ritonavir group, and this result was statistically significant (*p* < 0.05). In the comparison of AEs between Azvudine and the control group, the forest plot (Figure 5(b)) shows that the incidence of AEs in the Azvudine and control groups was 6.07% (106/1665) and 2.49% (54/2165), respectively. The OR between the two groups was 2.17 (95% CI: 0.72, 6.60), indicating that the incidence of AEs in the Azvudine group was 117% higher than in the control group. However, due to the wide CI, this result was not statistically significant (*p* > 0.05).

#### 3.2.3. Subgroup and Sensitivity Analyses

Subgroup analysis showed that Azvudine significantly reduced all-cause mortality in patients with long-term follow-up (> 14 days), significantly decreased the incidence of AEs in patients with a longer mean COVID-19 negative conversion time (> 7 days), and significantly lowered all-cause mortality in patients with mild infections, demonstrating significant clinical benefits for these patient groups (Table 8). Sensitivity analysis indicated low heterogeneity for all-cause mortality, with moderate heterogeneity observed in a few studies. By applying the “leave-one-out” approach, the estimated OR remained stable, further confirming the robustness of the primary analysis results (Figures S11–S24). Sensitivity analysis results for composite disease progression are presented in Figures S25–S38, and those for AEs are shown in Figures S39–S71.

#### 3.2.4. Risk of Bias and Certainty of Evidence

The GRADE assessment indicated that Azvudine significantly lowered all-cause mortality (OR: 0.54, 95% CI: 0.45, 0.64) but was associated with a higher incidence of composite disease progression (OR: 1.33, 95% CI: 1.14, 1.56) and AEs (OR: 1.28, 95% CI: 1.04, 1.58) compared to the control group. The evidence certainty was high for all-cause mortality and moderate for composite disease progression and AEs, warranting further validation in future studies (Table 9). The quality assessment of the included studies is presented in Table S2.

## 4. Discussion

Due to the high mutability and strong immune evasion capabilities of the COVID-19 virus, the global pandemic continues. Various treatment options exist for COVID-19 patients with different levels of severity. Although antiviral treatments have shown significant efficacy in some cases, particularly for mild to moderate patients, it is important to note that antiviral drugs may also cause side effects in patients. Therefore, there is a need to identify effective antiviral drugs that can reduce hospitalization time, time to nucleic acid conversion, and the incidence of composite outcomes (such as ICU admission, invasive mechanical ventilation, and patient mortality). Zhang [9] et al.'s study showed that in animal experiments, Azvudine reduced viral load, alleviated inflammation, and mitigated organ damage. A randomized single-arm clinical trial found that the side effects of Azvudine were mild, with only transient dizziness and nausea reported, and it also improved immunity.

In RCTs, Azvudine has been found to significantly reduce hospitalization time and time to nucleic acid conversion, primarily because most of the included patients had mild to moderate symptoms. This was consistent with the findings of Chen et al. [36] However, in the Zhang et al. [22] study, there was no significant difference in viral load and time to nucleic acid conversion between the intervention and control groups. This may be due to the higher proportion of severe patients or the higher rate of mild-to-moderate patients progressing to severe disease. Azvudine did not show efficacy in clearing the severe COVID-19 virus, thus failing to demonstrate significant clinical outcomes. Additionally, in Cabral et al.'s [23] study, 8 patients experienced all-cause mortality, with 7 showing disease progression (ICU admission). The all-cause mortality rates in the Azvudine and control groups were 3.26% (3/92) and 4.54% (4/88), respectively. This may be due to the fact that patients received Azvudine treatment 5 days after symptom onset, which is similar to the findings of the Shen et al. [31] study, where most patients received Azvudine treatment 5 days after symptom onset, and subgroup analysis did not show significant results. Our study is consistent with the findings of the meta-analysis conducted by Amani et al. [39, 40], which showed that Azvudine significantly reduced all-cause mortality compared to standard care/placebo (RR: 0.48, 95% CI: 0.40, 0.57). However, there was no significant difference between Azvudine and Nirmatrelvir/Ritonavir (OR: 0.84, 95% CI: 0.59, 1.21). Currently, the “Diagnosis and Treatment Protocol for Novel Coronavirus Infection (Trial Version 10)” issued by the National Health Commission of China specifies the dosage of Azvudine for adult COVID-19 patients as 5 mg/day, with a treatment course not exceeding 14 days. However, it does not specify the optimal time after symptom onset for initiating treatment.

In retrospective cohort studies, no significant difference in all-cause mortality or composite disease progression outcomes was found between the Azvudine and Nirmatrelvir/Ritonavir groups. This is consistent with previous studies showing comparable efficacy between Molnupiravir and Nirmatrelvir/Ritonavir in COVID-19 patients [41]. However, we were unable to identify significant differences between Azvudine and Nirmatrelvir/Ritonavir in treating COVID-19 patients, possibly because most studies were single-center studies [25–28]. In these studies, most deaths occurred in severe and critical patients, and the proportion of patients receiving treatment 5 days after symptom onset was much higher than those treated within 5 days. Additionally, some studies involved combination therapies [10], which may have affected the actual efficacy of Azvudine. In studies comparing Azvudine with the control group, the efficacy and safety of Azvudine were evident. Azvudine significantly reduced patient mortality and composite disease progression outcomes, and the incidence of AEs was not significantly different from the control group, indicating that Azvudine has no significant side effects on patients and does not adversely affect liver or kidney function.

In the safety analysis, Azvudine demonstrated a significant reduction in AEs among COVID-19 patients compared to Nirmatrelvir/Ritonavir. However, the incidence of AEs was comparable between the Azvudine and control groups, aligning with the findings of Amani et al.'s meta-analysis [39]. No statistically significant difference was observed in the incidence of severe AEs between the Azvudine and control groups, consistent with the research focus of Chen et al. [42]. The most frequently reported adverse reactions to Azvudine included dizziness, headache, nausea, abdominal pain, and elevated transaminase levels, with higher proportions of neurological, gastrointestinal, and hepatobiliary system involvement [38, 42, 43]. In the adverse clinical events reported by Chen et al. [35], 51 patients (15.36%) required ICU admission, 57 (17.17%) developed multi-organ dysfunction, 34 (10.24%) experienced arrhythmias, and 34 (10.24%) presented with shock. These outcomes showed no significant differences between the Azvudine and control groups.

Our meta-analysis demonstrated that Azvudine significantly reduced hospitalization time, time to nucleic acid conversion to negative, and all-cause mortality in patients with moderate COVID-19, while exhibiting a comparable safety profile to Nirmatrelvir/Ritonavir. Currently, Azvudine has been incorporated into the COVID-19 treatment guidelines by the National Health Commission and the National Administration of Traditional Chinese Medicine for use in treating adult patients with moderate COVID-19 symptoms [17, 18, 20] and has been adopted clinically in multiple countries, including China [21, 22, 25–38], Russia [22], and Brazil [23, 24]. Furthermore, Azvudine's relatively inexpensive cost (approximately U.S.$40 or CN¥270) per treatment course [44], combined with its efficacy and safety evidence, supports its prioritization as an antiviral option in clinical practice. However, it should be noted that existing studies mainly focus on the adult population, while research data on children, pregnant/lactating women, patients with moderate to severe liver and kidney function impairment, and immunocompromised populations are still insufficient. Medication in such populations requires caution, and it is recommended to use it under the guidance of doctors.

The strength of this study lies in its systematic review and meta-analysis of the efficacy and safety of Azvudine in treating COVID-19 patients. To reduce bias, this study separately analyzed RCTs and retrospective cohort studies. To demonstrate the efficacy of Azvudine, different control groups were categorized during the research process. However, this study has some limitations. Firstly, most of the included studies were conducted in China, with Chinese studies dominating, which reflects the regional focus on Azvudine, a domestically produced antiviral drug, in research. While this provides reliable data for the Chinese population, it limits the generalizability of our findings to other racial and ethnic groups. Secondly, there is a lack of RCTs, and most evidence comes from retrospective cohort studies, which may introduce selection bias. Thirdly, in retrospective cohort studies, the outcome measures and inclusion criteria varied significantly depending on the control group. Finally, most patients received treatment more than 5 days after symptom onset, which may affect the optimal efficacy of the drug.

## 5. Conclusions

Azvudine significantly reduced the hospitalization time and the time to nucleic acid conversion to negative in COVID-19 patients and significantly lowered all-cause mortality (GRADE: high-certainty evidence). In terms of safety, Azvudine demonstrated a favorable safety profile (GRADE: moderate-certainty evidence because of suspected publication bias and residual confounding). Further large-scale studies are needed to validate its efficacy and safety.

## Figures and Tables

**Table 1:** Characteristics of included RCTs.

Authors, title, journal name, published year, country	Clinical trials registration	Phase	Center	Study population	Gender	Age (years)	Group and sample size			
							Intervention group		Control group	
Ren Z, Luo H, Yu Z, Song J, Liang L, Wang L, Wang H, Cui G, Liu Y, Wang J, Li Q, Zeng Z, Yang S, Pei G, Zhu Y, Song W, Yu W, Song C, Dong L, Hu C, Du J, Chang J. A Randomized, Open-Label, Controlled Clinical Trial of Azvudine Tablets in the Treatment of Mild and Common COVID-19, a Pilot Study. *Adv Sci (Weinh)*. 2020, China [21]	ChiCTR2000029853	3	Single center	Patients with mild and common COVID-19	M: 12 (60%) F: 8 (40%)	52.0 (17–61) vs. 50.5 (29–76)	Azvudine 5 mg + standard treatment	10	Placebo + standard treatment	10

Zhu KW. Efficacy and safety evaluation of Azvudine in the prospective treatment of COVID-19 based on four phase III clinical trials. *Front Pharmacol.* 2023, China [22]	NCT04425772 ChiCTR2000032769	3	Multicenter	Patients with mild, common, and severe COVID-19	NR	18 years and older	Azvudine 5 mg + standard treatment	174	FNC dummy tablet + standard treatment	174

de Souza SB, Cabral PGA, da Silva RM, Arruda RF, Cabral SPF, de Assis ALEM, Viana Junior AB, Degrave WMS, Moreira ADS, Silva CG, Chang J, Lei P. Phase III, randomized, double-blind, placebo-controlled clinical study: a study on the safety and clinical efficacy of AZVUDINE in moderate COVID-19 patients. *Front Med (Lausanne)*. 2023, Brazil [23]	NCT04668235	3	Single center	Patients with moderate COVID-19	M: 104 (58.1%) F: 75 (41.9%)	51 ± 13 (48) vs. 48 ± 13 (48)	Azvudine 5 mg + standard treatment	91	Placebo + standard treatment	88

da Silva RM, Gebe Abreu Cabral P, de Souza SB, Arruda RF, Cabral SPF, de Assis ALEM, Martins YPM, Tavares CAA, Viana Junior AB, Chang J, Lei P. Serial viral load analysis by DDPCR to evaluate FNC efficacy and safety in the treatment of mild cases of COVID-19. *Front Med (Lausanne)*. 2023, Brazil [24]	NCT05033145	3	Multicenter	Patients with mild COVID-19	M: 111 (40%) F: 170 (60%)	45 ± 16 (43) vs. 45 ± 15 (46)	Azvudine 5 mg + standard treatment	143	Placebo + standard treatment	138

Zhu KW. Efficacy and safety evaluation of Azvudine in the prospective treatment of COVID-19 based on four phase III clinical trials. *Front Pharmacol.* 2023, Russia [22]	License No. 20 of the Ministry of Health of the Russian Federation	3	NR	Patients with mild, common and severe COVID-19	M: 135 (43%) F: 179 (57%)	18 Years and older	Azvudine	157	Control	157

Abbreviation: NR, not reported.

**Table 2:** Characteristics of included ongoing studies.

Date of registration	Clinical trials registration	Status	Phase	Center	Study type	Intervention group	Control group	Age range	Sample size
11/06/2021	ChiCTR2100052875	Pending	3	Single center	Interventional study	Azvudine + symptomatic treatment	Antiviral therapy + symptomatic treatment	18–60	30:30
11/30/2022	NCT05633433	Recruiting	2,3	Multicenter	Interventional study	Azvudine	Placebo	18 years and older	1550
12/07/2022	NCT05642910	Recruiting	NR	Single center	Interventional study	Azvudine	Paxlovid	18–85	540
12/28/2022	ChiCTR2200067174	Pending	4	Single center	Interventional study	Azovudine + Traditional Chinese medicine	Traditional Chinese medicine	15–65	50:50
01/10/2023	NCT05682599	Recruiting	2	Multicenter	Interventional study	Azvudine	Placebo	18–65	300
01/17/2023	NCT05689034	Not yet recruiting	2,3	Multicenter	Interventional study	Azvudine	Placebo	18–120	1096
01/20/2023	NCT05697055	Recruiting	4	Multicenter	Interventional study	Azvudine	Nirmatrelvir-Ritonavir	18 Years and older	410
02/27/2023	ChiCTR2300068643	Recruiting	4	Single center	Interventional study	Azvudine 5 mg + routine treatment	Nirmatrelvir 300 mg + Ritonavir 100 mg + routine treatment	18–90	46:46

*Note:* D-H group: Deuremidevir Hydrobromide (VV116) group; S-R group: Simnotrelvir/ritonavir (Xiannuoxin) group; U-D group: The untreated small-molecule drug (blank control) group.

Abbreviations: NR, not reported; N-R group, Nirmatrelvir/Ritonavir group.

**Table 3:** Characteristics of included retrospective cohort studies.

Authors, title, journal name, published year, country	Time	Center	Gender	Age (years)	Group and sample size			
					Intervention group		Control group	
Zhao X, Cheng Y, Zhang M, Qianda B, Zhouma B, Yangzhen B, Zheng Y, Zhang S, Zhao H. Efficacy of Nirmatrelvir-Ritonavir versus Azvudine for COVID-19 Treatment in Tibet: A Retrospective Study. *Infect Drug Resist.* 2023[a], China [25]	08/01/2022–09/30/2022	Single center	M: 105 (46.25%) F: 122 (53.75%)	46.5 vs. 51.0	Azvudine 5 mg	82	Nirmatrelvir + 300 mg ritonavir + 100 mg	145
Wei AH, Zeng L, Wang L, Gui L, Zhang WT, Gong XP, Li J, Liu D. Head-to-head comparison of azvudine and nirmatrelvir/ritonavir for the hospitalized patients with COVID-19: a real-world retrospective cohort study with propensity score matching. *Front Pharmacol.* 2023, China [26]	12/01/2022–01/31/2023	Single center	M: 467 (64.41%) F: 258 (35.59%)	68.0 vs. 65.0	Azvudine	461	Nirmatrelvir + 300 mg ritonavir + 100 mg	264
Deng G, Li D, Sun Y, Jin L, Zhou Q, Xiao C, Wu Q, Sun H, Dian Y, Zeng F, Pan P, Shen M. Real-world effectiveness of Azvudine versus nirmatrelvir-ritonavir in hospitalized patients with COVID-19: A retrospective cohort study. *J Med Virol.* 2023, China [27]	12/05/2022–01/31/2023	Single center	M: 348 (61.92%) F: 214 (38.08%)	67.5 (14.5) vs. 67.4 (14.7)	Azvudine	281	Nirmatrelvir–ritonavir	281
Zhao Q, Zheng B, Han B, Feng P, Xia Z, Jiang H, Ying Y, Zhu J, Fei C, Xiang J, Shen L, Luo Q, Wu Y, Wusiman A, Xin C, Zhang M, Li G, Li X. Is Azvudine Comparable to Nirmatrelvir-Ritonavir in Real-World Efficacy and Safety for Hospitalized Patients with COVID-19? A Retrospective Cohort Study. *Infect Dis Ther.* 2023 (b), China [28]	12/20/2022–01/31/2023	Single center	M: 175 (61.19%) F: 111 (38.81%)	76.45 (12.1) vs. 76.83 (13.5)	Azvudine 5 mg	143	Nirmatrelvir + 300 mg ritonavir + 100 mg	143
Han X, Gao D, Li C, Yuan X, Cui J, Zhao W, Xie F, Wang K, Liu Y, Muo G, Xi N, Zheng M, Wang R, Xiao K, Zhao D, Zhang X, Han X, Wang B, Zhang T, Xie W, Xie L. Real-world effectiveness of nirmatrelvir-ritonavir versus azvudine in hospitalized patients with COVID-19 during the omicron wave in Beijing: a multicenter retrospective cohort study. *BMC Infect Dis.* 2024, China [29]	12/10/2022–02/20/2023	Multicenter	M: 622 (63.99%) F: 350 (36.01%)	68.0 (19.9) vs. 67.9 (17.4)	Azvudine 5 mg	486	Nirmatrelvir + 300 mg ritonavir + 100 mg	486
Shang S, Fu B, Geng Y, Zhang J, Zhang D, Xiao F, Sheng Z, Zhai J, Li W, Chen X, Zheng C, Li Q. Azvudine therapy of common COVID-19 in hemodialysis patients. *J Med Virol.* 2023, China [30]	11/01/2022–01/10/2023	Multicenter	M: 183 (50.27%) F: 181 (49.73%)	54.0 (6.0) vs. 55.0 (5.0)	Azvudine 5 mg	182	Basic treatment	182
Shen M, Xiao C, Sun Y, Li D, Wu P, Jin L, Wu Q, Dian Y, Meng Y, Zeng F, Chen X, Deng G. Real-world effectiveness of Azvudine in hospitalized patients with COVID-19: a retrospective cohort study. *medRxiv*. 2023, China [31]	12/05/2022–01/13/2023	Single center	M: 261 (57.74%) F: 191 (42.26%)	64.4 (14.6) vs. 65.3 (15.4)	Azvudine + standard treatment	226	Standard treatment	226
Qi X, Yang Y, Gong B, Li Z, Liang D. Real-world effectiveness of azvudine for patients infected with the SARS-CoV-2 omicron subvariant BA.5 in an intensive care unit. *J Thorac Dis*. 2023, China [32]	08/13/2022–09/07/2022	Single center	M: 9 (69.23%) F: 4 (30.77%)	72.33 (17.4) vs. 65.14 (17.9)	Azvudine 5 mg	6	Non-Azvudine	7
Liu B, Yang M, Xu L, Li Y, Cai J, Xie B, Zong K, Guo S. Azvudine and mortality in patients with coronavirus disease 2019: A retrospective cohort study. *Int Immunopharmacol*. 2023, China [33]	12/01/2022–01/20/2023	Multicenter	M: 642 (89.54%) F: 75 (10.46%)	67.8 (16.4) vs. 70.0 (15.4)	Azvudine 5 mg	229	Non-Azvudine	488
Zong K, Zhou H, Li W, Jiang E, Liu Y, Li S. Azvudine reduces the in-hospital mortality of COVID-19 patients: A retrospective cohort study. *Acta Pharm Sin B.* 2023, China [34]	12/08/2022–01/20/2023	Multicenter	M: 329 (56.24%) F: 256 (43.76%)	67.8 (16.0) vs. 68.2 (16.6)	Azvudine	195	Non-Azvudine	390
Chen R, Guo Y, Deng S, Wang J, Gao M, Han H, Wang L, Jiang H, Huang K. All-cause mortality in moderate and severe COVID-19 patients with myocardial injury receiving versus not receiving azvudine: a propensity score-matched analysis. *Cardiol Plus*. 2023[a], China [35]	12/07/2022–12/30/2022	Single center	M: 140 (70.71%) F: 58 (29.29%)	18 years and older	Azvudine	99	Without Azvudine	99
Chen W, Xu H, Hong L, Yang R, Peng C, Wang G, Li W. Oral Azvudine (FNC) Tablets in Patients infected with SARS-CoV-2 Omicron Variant: A Retrospective Cohort Study. *medRxiv*. 2023 (b), China [36]	08/01/2022–09/30/2022	Multicenter	M: 95 (45.89%) F: 112 (54.11%)	36.0 vs. 29.0	Azvudine	166	Control group	41
Sun Y, Jin L, Dian Y, Shen M, Zeng F, Chen X, Deng G. Oral Azvudine for hospitalised patients with COVID-19 and pre-existing conditions: a retrospective cohort study. *EClinicalMedicine*. 2023, China [37]	12/05/2022–01/31/2023	Single center	M: 311 (63.47%) F: 179 (36.53%)	69.13 (13.4) vs. 69.25 (14.0)	Azvudine 5 mg	245	Control group	245
Yang H, Wang Z, Jiang C, Zhang Y, Zhang Y, Xu M, Zhang Y, Wang Y, Liu X, An Z, Tong Z. Oral azvudine for mild-to-moderate COVID-19 in high risk, nonhospitalized adults: Results of a real-world study. *J Med Virol.* 2023, China [38]	12/19/2022–01/05/2023	Single center	M: 445 (55.35%) F: 35 9 (44.65%)	67.0 VS 61.0	Azvudine	317	Control group	487

**Table 4:** Outcome metrics in included RCTs.

Authors, title, journal name, published year, country	Outcome metrics
Ren Z, Luo H, Yu Z, Song J, Liang L, Wang L, Wang H, Cui G, Liu Y, Wang J, Li Q, Zeng Z, Yang S, Pei G, Zhu Y, Song W, Yu W, Song C, Dong L, Hu C, Du J, Chang J. A Randomized, Open-Label, Controlled Clinical Trial of Azvudine Tablets in the Treatment of Mild and Common COVID-19, a Pilot Study. *Adv Sci (Weinh)*. 2020, China [21]	(1) Adverse events (0 patient vs. 3 patients, *p* < 0.06); (2) time to nucleic acid conversion to negative (2.60 ± 0.97 days vs. 5.60 ± 3.06 days, *p*=0.008); (3) nucleic acid conversion to negative rate (4 day, *p* < 0.0013); (4) improvement rate of chest CT image (5 days vs. 8 days, *p*=0.0401); (5) improvement in respiratory symptoms and signs; and (6) time of body temperature returning to normality.
Zhu KW. Efficacy and safety evaluation of Azvudine in the prospective treatment of COVID-19 based on four phase III clinical trials. *Front Pharmacol.* 2023, China [22]	(1) Adverse events (62 patients vs. 76 patients); (2) time to nucleic acid conversion to negative; (3) viral load (day 5, *p* < 0.001); (4) nucleic acid conversion to negative rate; (5) proportion of patients changing from mild-or-moderate COVID-19 to severe COVID-19 in the severity of the disease; (6) proportion of patients changing from severe COVID-19 to critical COVID-19 in severity; (7) time and proportion of improvement in pulmonary imaging; (8) time and rate of improvement in respiratory symptoms and other symptoms; (9) frequency of requirement for supplemental oxygen or noninvasive ventilation; changes in blood oxygen detection index; and (10) time and proportion of temperature return to normal levels.
de Souza SB, Cabral PGA, da Silva RM, Arruda RF, Cabral SPF, de Assis ALEM, Viana Junior AB, Degrave WMS, Moreira ADS, Silva CG, Chang J, Lei P. Phase III, randomized, double-blind, placebo-controlled clinical study: a study on the safety and clinical efficacy of AZVUDINE in moderate COVID-19 patients. *Front Med (Lausanne)*. 2023, Brazil [23]	(1) Adverse events (50 patients vs. 55 patients); (2) serious adverse events (3 patients vs. 5 patients); (3) time to nucleic acid conversion to negative (6.24 days vs. 7.94 days, *p*=0.002); (4) viral load on day 5 (RT-PCR: 2.355 ± 4.454 vs. 3.173 ± 4.960, *p* < 0.331; DDPCR: 0 [0, 202] vs. 284 [14, 16,827], *p* < 0.001); (5) clinical improvement score (0.02 ± 0.15 vs. 0.11 ± 0.31, *p* < 0.024); (6) time to improvement of symptoms; and (7) time and proportion of lung imaging improvement.
da Silva RM, Gebe Abreu Cabral P, de Souza SB, Arruda RF, Cabral SPF, de Assis ALEM, Martins YPM, Tavares CAA, Viana Junior AB, Chang J, Lei P. Serial viral load analysis by DDPCR to evaluate FNC efficacy and safety in the treatment of mild cases of COVID-19. *Front Med (Lausanne)*. 2023, Brazil [24]	(1) Adverse events (110 patients vs. 112 patients); (2) serious adverse events (1 patient vs. 0 patient); (3) time to nucleic acid conversion to negative (5.55 days vs. 8.27 days, *p* < 0.001); (4) viral load on day 5 (RT-PCR: 3.016 ± 4.897 vs. 5.513 ± 5.187, *p* < 0.001; DDPCR: 3828 ± 15,666 vs. 38,783 ± 70,409, *p* < 0.001); (5) clinical improvement score (*p*=0.700); and (6) time to improvement of symptoms.
Zhu KW. Efficacy and safety evaluation of Azvudine in the prospective treatment of COVID-19 based on four phase III clinical trials. *Front Pharmacol.* 2023, Russia [22]	(1) Adverse events (34 patients vs. 35 patients); (2) serious adverse events (0 patient vs. 1 patient); (3) median time to improvement in clinical conditions (10 days vs. 13 days, *p* < 0.001); and (4) proportion of improvement in clinical conditions on day 7 (57 patients vs. 15 patients, *p* < 0.001).

*Note:* A versus B (A: Azvudine group; B: control group).

**Table 5:** Subgroup analysis of included RCTs.

Subgroups	Number of studies	Number of people	RR	95% CI	*p* value
Country					
China [21, 22]					
Adverse events	2	368	0.78	0.60 to 1.02	0.07
Serious adverse events	2	368	0.33	0.03 to 3.18	0.34
Brazil [23, 24]					
Adverse events	2	460	0.93	0.82 to 1.04	0.19
Serious adverse events	2	460	0.77	0.21 to 2.84	0.70
Russia [22]					
Adverse events	1	314	0.97	0.64 to 1.47	0.89
Serious adverse events	1	314	0.33	0.01 to 8.12	0.50
Disease severity					
Mild	3	649	1.03	0.85 to 1.26	0.75
Moderate	4	861	1.01	0.79 to 1.30	0.91

**Table 6:** GRADE evidence profile for outcomes in RCTs.

Certainty assessment							No. of patients		Effect		Certainty	Importance
No. of studies	Study design	Risk of bias	Inconsistency	Indirectness	Imprecision	Other considerations	Azvudine	Control	Relative (95% CI)	Absolute (95% CI)		
*Adverse events*												
5	Randomized trials	Not serious	Not serious	Not serious	Serious^a^	Publication bias is strongly suspected; all plausible residual confounding would reduce the demonstrated effect^b^	256/575 (44.5%)	281/567 (49.6%)	**RR 0.89** (0.80–1.00)	**55 fewer per 1000** (from 99 fewer to 0 fewer)	⨁⨁⨁◯ Moderate^a,b^	CRITICAL

*Serious adverse events*												
5	Randomized trials	Not serious	Not serious	Not serious	Serious^a^	Publication bias is strongly suspected; all plausible residual confounding would reduce the demonstrated effect^b^	4/575 (0.7%)	6/567 (1.1%)	**RR 0.69** (0.22–2.15)	**3 fewer per 1000** (from 8 fewer to 12 more)	⨁⨁⨁◯ Moderate^a,b^	CRITICAL

Abbreviations: CI, confidence interval; RR, risk ratio.

^a^The findings of RCTs are less precise due to the single research center and small sample size, which contribute to the imprecision of the study results.

^b^The significance of research results can influence the choice of journal.

**Table 7:** Outcome metrics in included retrospective cohort studies.

Authors, title, journal name, published year, country	Outcome metrics
Zhao X, Cheng Y, Zhang M, Qianda B, Zhouma B, Yangzhen B, Zheng Y, Zhang S, Zhao H. Efficacy of Nirmatrelvir-Ritonavir versus Azvudine for COVID-19 Treatment in Tibet: A Retrospective Study. *Infect Drug Resist.* 2023[a], China [25]	(1) All-cause mortality (3 patients vs. 4 patients, *p*=0.706); (2) time to nucleic acid conversion to negative (9.0 [6.0,12.0] days vs. 7.0 [11.0,15.0] days, *p*=0.064); and (3) length of hospitalization (8.0 [5.0,10.0] days vs. 8.0 [5.5,10.5] days, *p*=0.378).
Wei AH, Zeng L, Wang L, Gui L, Zhang WT, Gong XP, Li J, Liu D. Head-to-head comparison of azvudine and nirmatrelvir/ritonavir for the hospitalized patients with COVID-19: a real-world retrospective cohort study with propensity score matching. *Front Pharmacol.* 2023, China [26]	(1) All-cause mortality (63 patients vs. 36 patients, *p*=0.991); (2) composite outcome (98 patients vs. 72 patients, *p*=0.066); (3) adverse events (33 patients vs. 22 patients, *p*=0.656); (4) intensive care unit admission (11 patients vs. 14 patients, *p*=0.038); and (5) invasive mechanical ventilation use (77 patients vs. 61 patients, *p*=0.035).
Deng G, Li D, Sun Y, Jin L, Zhou Q, Xiao C, Wu Q, Sun H, Dian Y, Zeng F, Pan P, Shen M. Real-world effectiveness of Azvudine versus nirmatrelvir-ritonavir in hospitalized patients with COVID-19: A retrospective cohort study. *J Med Virol.* 2023, China [27]	(1) All-cause mortality (6 patients vs. 17 patients, *p*=0.052); (2) composite outcome (21 patients vs. 38 patients, *p*=0.026); (3) intensive care unit admission (2 patients vs. 5 patients, *p*=0.273); (4) invasive mechanical ventilation use (3 patients vs. 10 patients, *p*=0.067); and (5) need for high‐flow oxygen therapy (12 patients vs. 14 patients, *p*=0.815).
Zhao Q, Zheng B, Han B, Feng P, Xia Z, Jiang H, Ying Y, Zhu J, Fei C, Xiang J, Shen L, Luo Q, Wu Y, Wusiman A, Xin C, Zhang M, Li G, Li X. Is Azvudine Comparable to Nirmatrelvir-Ritonavir in Real-World Efficacy and Safety for Hospitalized Patients with COVID-19? A Retrospective Cohort Study. Infect Dis Ther. 2023 (b), China [28]	(1) All-cause mortality (9 patients vs. 15 patients); (2) adverse events (5 patients vs. 12 patients, *p*=0.538); (3) time to nucleic acid conversion to negative (16.0 [12.0,21.0] days vs. 15.0 [10.0,21.0] days); (4) length of hospitalization (15.0 [10.0,20.0] days vs. 14.0 [9.0,23.0] days); (5) risk of progression to a critical condition (13 patients vs. 19 patients).
Han X, Gao D, Li C, Yuan X, Cui J, Zhao W, Xie F, Wang K, Liu Y, Muo G, Xi N, Zheng M, Wang R, Xiao K, Zhao D, Zhang X, Han X, Wang B, Zhang T, Xie W, Xie L. Real-world effectiveness of nirmatrelvir-ritonavir versus azvudine in hospitalized patients with COVID-19 during the omicron wave in Beijing: a multicenter retrospective cohort study. *BMC Infect Dis.* 2024, China [29]	(1) All-cause mortality (43 patients vs. 36 patients); (2) composite outcome (49 patients vs. 42 patients, *p*=0.538); (3) newly onset severe liver injury (1.9% vs. 3.5%); (4) newly onset severe kidney injury (2.7% vs. 3.3%); and (5) clinical improvement (389 patients vs. 376 patients).
Shang S, Fu B, Geng Y, Zhang J, Zhang D, Xiao F, Sheng Z, Zhai J, Li W, Chen X, Zheng C, Li Q. Azvudine therapy of common COVID-19 in hemodialysis patients. *J Med Virol.* 2023, China [30]	(1) Adverse events (10 patients vs. 11 patients) and (2) improvement rate of chest CT image (10 days vs. 15 days, *p* < 0.001).
Shen M, Xiao C, Sun Y, Li D, Wu P, Jin L, Wu Q, Dian Y, Meng Y, Zeng F, Chen X, Deng G. Real-world effectiveness of Azvudine in hospitalized patients with COVID-19: a retrospective cohort study. *medRxiv*. 2023, China [31]	(1) All-cause mortality (3 patients vs. 10 patients, *p*=0.027); (2) composite outcome (8 patients vs. 17 patients, *p*=0.041); (3) intensive care unit admission (1 patient vs. 4 patients, *p*=0.149); (4) invasive mechanical ventilation use (0 patient vs. 5 patients, *p*=0.020); and (5) need for high‐flow oxygen therapy (6 patients vs. 7 patients, *p*=0.641).
Qi X, Yang Y, Gong B, Li Z, Liang D. Real-world effectiveness of azvudine for patients infected with the SARS-CoV-2 omicron subvariant BA.5 in an intensive care unit. *J Thorac Dis.* 2023, China [32]	(1) All-cause mortality (1 patient vs. 0 patient) and (2) time to nucleic acid conversion to negative (14.11 ± 5.74 days vs. 16.33 ± 6.67 days).
Liu B, Yang M, Xu L, Li Y, Cai J, Xie B, Zong K, Guo S. Azvudine and mortality in patients with coronavirus disease 2019: A retrospective cohort study. *Int Immunopharmacol*. 2023, China [33]	(1) All-cause mortality (30 patients vs. 206 patients).
Zong K, Zhou H, Li W, Jiang E, Liu Y, Li S. Azvudine reduces the in-hospital mortality of COVID-19 patients: A retrospective cohort study. *Acta Pharm Sin B.* 2023, China [34]	(1) All-cause mortality (11% vs. 24%, *p* < 0.001) and (2) length of hospitalization (9.4 ± 6.4 days vs. 8.4 ± 5.2 days, *p*=0.345).
Chen R, Guo Y, Deng S, Wang J, Gao M, Han H, Wang L, Jiang H, Huang K. All-cause mortality in moderate and severe COVID-19 patients with myocardial injury receiving versus not receiving azvudine: a propensity score-matched analysis. *Cardiol Plus*. 2023[a], China [35]	(1) All-cause mortality (29 patients vs. 31 patients, *p*=0.757); (2) transnasal high-flow oxygen inhalation use (26 patients vs. 19 patients, *p*=0.235); (3) noninvasive ventilation use (24 patients vs. 16 patients, *p*=0.157); (4) invasive mechanical ventilation use (12 patients vs. 10 patients, *p*=0.651); and (5) extra corporeal membrane oxygenation (0 patient vs. 1 patient, *p*=0.316).
Chen W, Xu H, Hong L, Yang R, Peng C, Wang G, Li W. Oral Azvudine (FNC) Tablets in Patients infected with SARS-CoV-2 Omicron Variant: A Retrospective Cohort Study. *medRxiv*. 2023 (b), China [36]	(1) Adverse events (9 patients vs. 0 patient, *p*=0.273); (2) time to nucleic acid conversion to negative (5.0 [1.0,7.0] days vs. 6.0 [5.0,7.0] days, *p*=0.001); and (3) length of hospitalization (12.0 [8.0,15.0] days vs. 12.0 [10.0,14.0] days, *p*=0.793).
Sun Y, Jin L, Dian Y, Shen M, Zeng F, Chen X, Deng G. Oral Azvudine for hospitalised patients with COVID-19 and pre-existing conditions: a retrospective cohort study. *EClinicalMedicine*. 2023, China [37]	(1) All-cause mortality (5 patients vs. 9 patients, *p*=0.159); (2) composite outcome (17 patients vs. 31 patients, *p*=0.018); (3) intensive care unit admission (2 patients vs. 1 patient, *p*=0.911); (4) invasive mechanical ventilation use (2 patients vs. 2 patients, *p*=0.728); and (5) noninvasive respiratory support (16 patients vs. 26 patients, *p*=0.056).
Yang H, Wang Z, Jiang C, Zhang Y, Zhang Y, Xu M, Zhang Y, Wang Y, Liu X, An Z, Tong Z. Oral azvudine for mild-to-moderate COVID-19 in high risk, nonhospitalized adults: Results of a real-world study. *J Med Virol.* 2023, China [38]	(1) All-cause mortality (0 patient vs. 6 patients); (2) composite outcome (4 patients vs. 14 patients, *p*=0.002); (3) adverse events (82 patients vs. 43 patients, *p* < 0.001); (4) rate of hospitalization (2.36/10,000 person-days vs. 9.35/10,000 person-days, *p*=0.047); and (5) longer duration of fever (3.79 ± 2.44 days vs. 5.50 ± 11.31 days, *p*=0.013).

*Note:* A versus B (A: Azvudine group; B: control group).

**Table 8:** Subgroup analysis of included retrospective cohort studies.

Subgroups	Number of studies	Number of people	OR	95% CI	*p* value
Follow-up duration					
≤ 14 days	3	967	1.39	1.01 to 1.90	0.04
> 14 days	8	3941	0.65	0.51 to 0.82	< 0.05
Mean COVID-19 negative conversion time					
≤ 7 days	2	434	1.87	0.59 to 6.00	0.29
> 7 days	5	1097	0.00	0.00 to 0.05	< 0.05
Time from symptom onset to treatment exposure					
≤ 5 days	6	3566	1.14	0.95 to 1.37	0.17
> 5 days	4	2476	0.97	0.80 to 1.18	0.77
Disease severity					
Mild	8	3551	0.69	0.60 to 0.81	< 0.05
Moderate	12	5266	1.66	1.46 to 1.88	< 0.05
Severe	11	4462	0.99	0.88 to 1.11	0.81
Critical	6	2465	1.48	1.19 to 1.85	< 0.05
Vaccination status					
Unvaccinated	4	2083	1.66	1.39 to 1.99	< 0.05
Vaccinated	4	2083	1.01	0.85 to 1.21	0.91

**Table 9:** GRADE evidence profile for outcomes in cohort studies.

Certainty assessment							No. of patients		Effect		Certainty	Importance
No. of studies	Study design	Risk of bias	Inconsistency	Indirectness	Imprecision	Other considerations	Azvudine	Control	Relative (95% CI)	Absolute (95% CI)		
*All-cause mortality*												
14	Cohort studies	Not serious	Not serious	Not serious	Not serious	All plausible residual confounding would reduce the demonstrated effect	213/3125 (6.8%)	464/3870 (12.0%)	**OR 0.54** (0.45–0.64)	**51 fewer per 1000** (from 62 fewer to 40 fewer)	⨁⨁⨁⨁ High	CRITICAL

*Composite disease progression*												
14	Cohort studies	Not serious	Not serious	Not serious	serious^a^	Publication bias is strongly suspected; all plausible residual confounding would reduce the demonstrated effect^b^	376/3118 (12.1%)	325/3484 (9.3%)	**OR 1.33** (1.14–1.56)	**27 more per 1000** (from 12 more to 45 more)	⨁⨁⨁◯ Moderate^a,b^	CRITICAL

*Adverse events*												
14	Cohort studies	Not serious	Not serious	Not serious	serious^a^	Publication bias is strongly suspected; all plausible residual confounding would reduce the demonstrated effect^b^	196/3118 (6.3%)	173/3484 (5.0%)	**OR 1.28** (1.04–1.58)	**13 more per 1000** (from 2 more to 27 more)	⨁⨁⨁◯ Moderate^a,b^	CRITICAL

Abbreviations: CI, confidence interval; OR, odds ratio.

^a^Some studies did not achieve precise matching, which resulted in inadequate control of confounding factors between the Azvudine group and the control groups.

^b^The significance of research results can influence the choice of journal.

## Data Availability

Data are available on request from the authors.

## Appendix 1: Search Strategy (Using PubMed Database as an Example)

  #1. (COVID-19[MeSH Terms]) AND (Azvudine[All Fields])
  #2. (COVID-19[MeSH Terms]) AND (FNC[All Fields])
  #3. (SARS-CoV-2[MeSH Terms]) AND (Azvudine[All Fields])
  #4. (SARS-CoV-2[MeSH Terms]) AND (FNC[All Fields])
  #5. #1 OR #2 OR #3 OR #4

## Supporting Information

Figure S1–S5. Sensitivity analysis of adverse events in RCTs.

Figure S6–S10. Sensitivity analysis of serious adverse events in RCTs.

Figure S11–S24. Sensitivity analysis of all-cause mortality in retrospective cohort studies.

Figure S25–S38. Sensitivity analysis of composite disease progression in retrospective cohort studies.

Figure S39–S52. Sensitivity analysis of adverse events in retrospective cohort studies.

Figure S53–S71. Sensitivity analysis of adverse events in all included studies.

Table S1. Quality assessment of included RCTs.

Table S2. Quality assessment of included retrospective cohort studies.

Table S3. Severity of COVID-19 upon admission of patients.

Figure S72. Stacked graph of basic disease in patients in the Azvudine group and control group.
