# Supplementary material for: Real-World Evaluation Study of Azvudine for the Treatment of Patients With COVID-19: A Systematic Review and Meta-Analysis
Source: Can J Infect Dis Med Microbiol. 2025 Sep 26;2025:3645253. doi: 10.1155/cjid/3645253 (PMC12494476; doi:10.1155/cjid/3645253)
Supplement: Supporting Information — Additional supporting information can be found online in the Supporting Information section. [file 3645253.f1.docx]

**CONTENT**

[Ⅰ. Sensitivity analysis 1](#_Toc13430)

[1. Adverse events in RCTs 1](#_Toc5514)

[2. Serious adverse events in RCTs 3](#_Toc14757)

[3. All cause mortality in Retrospective cohort studies 4](#_Toc1971)

[4. Composite disease progression in Retrospective cohort studies 10](#_Toc3846)

[5. Adverse events in Retrospective cohort studies 14](#_Toc32618)

[6. Adverse events in all included studies 19](#_Toc4644)

[Ⅱ. Supplementary table 29](#_Toc1002)

[Table S1. Quality assessment of included studies. 29](#_Toc19434)

[Table S2. Quality assessment of included studies. 30](#_Toc3426)

[Table S3. Severity of COVID-19 upon admission of patients. 32](#_Toc17023)

[Ⅲ. Supplementary figure 33](#_Toc9414)

[Figure S72. Stacked graph of basic disease in patients in the Azvudine group and control group. 33](#_Toc28294)

# Ⅰ. Sensitivity analysis

1. Adverse events in RCTs


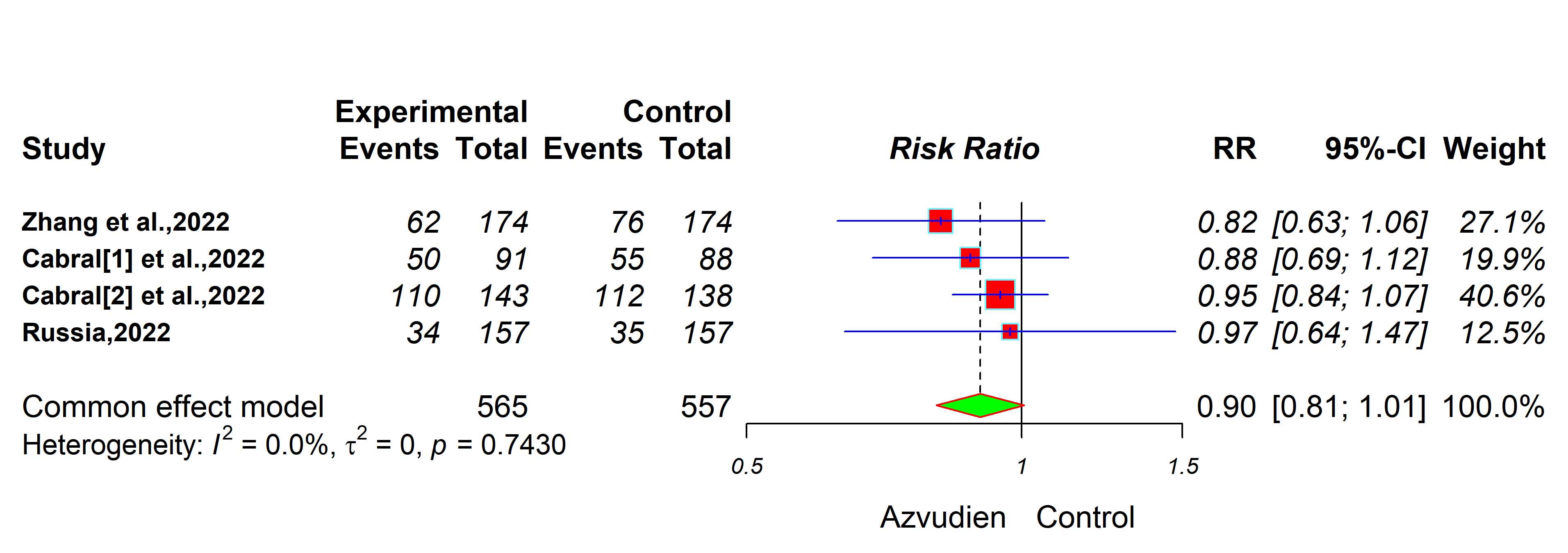


Figure S1. Adverse events (Removing Ren et al.,2020).


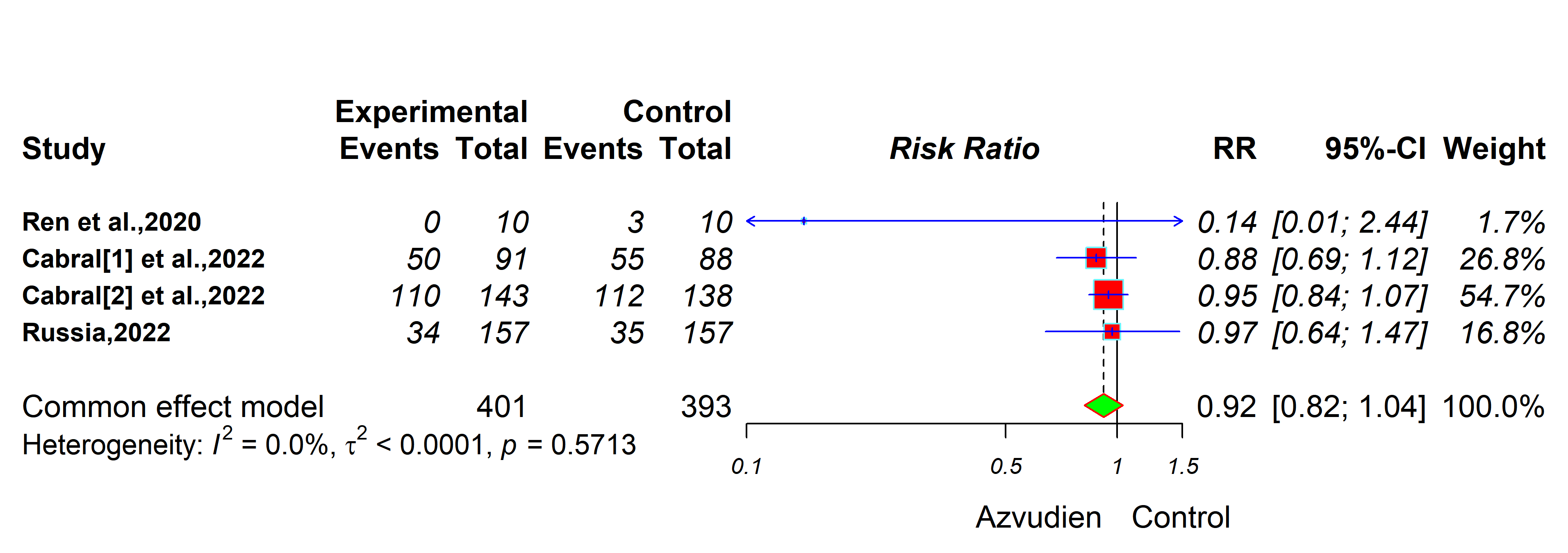


Figure S2. Adverse events (Removing Zhang et al.,2022).


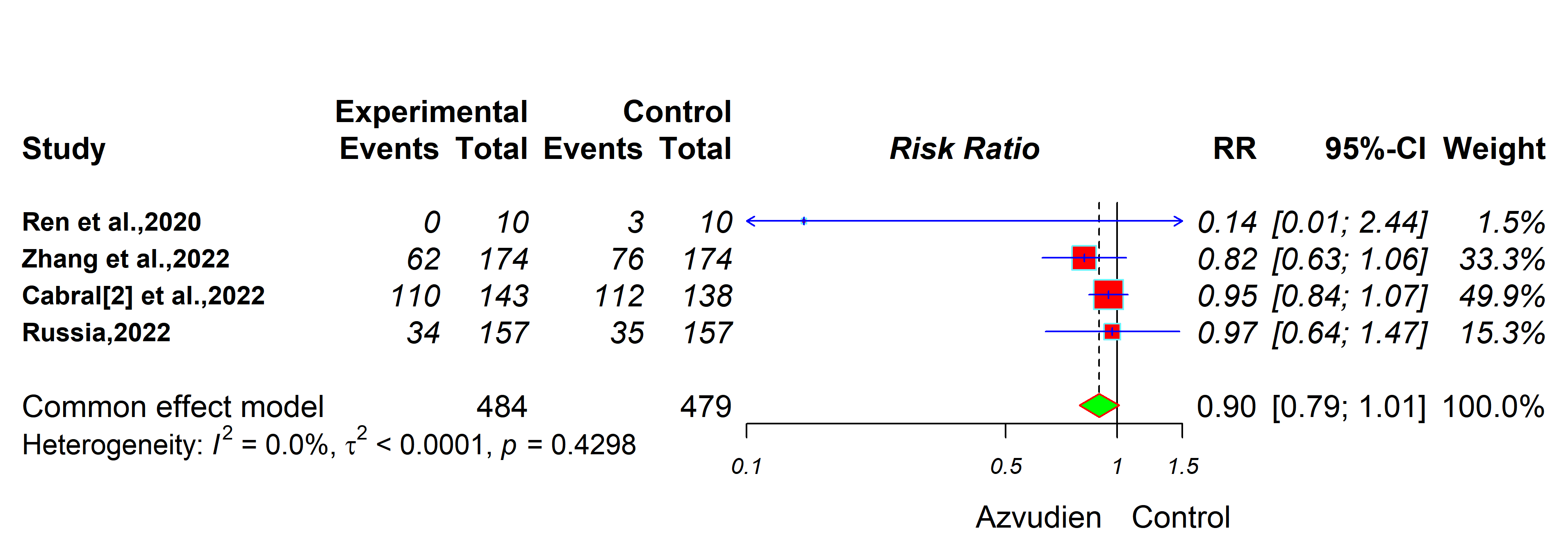


Figure S3. Adverse events (Removing Cabral[1] et al.,2022).


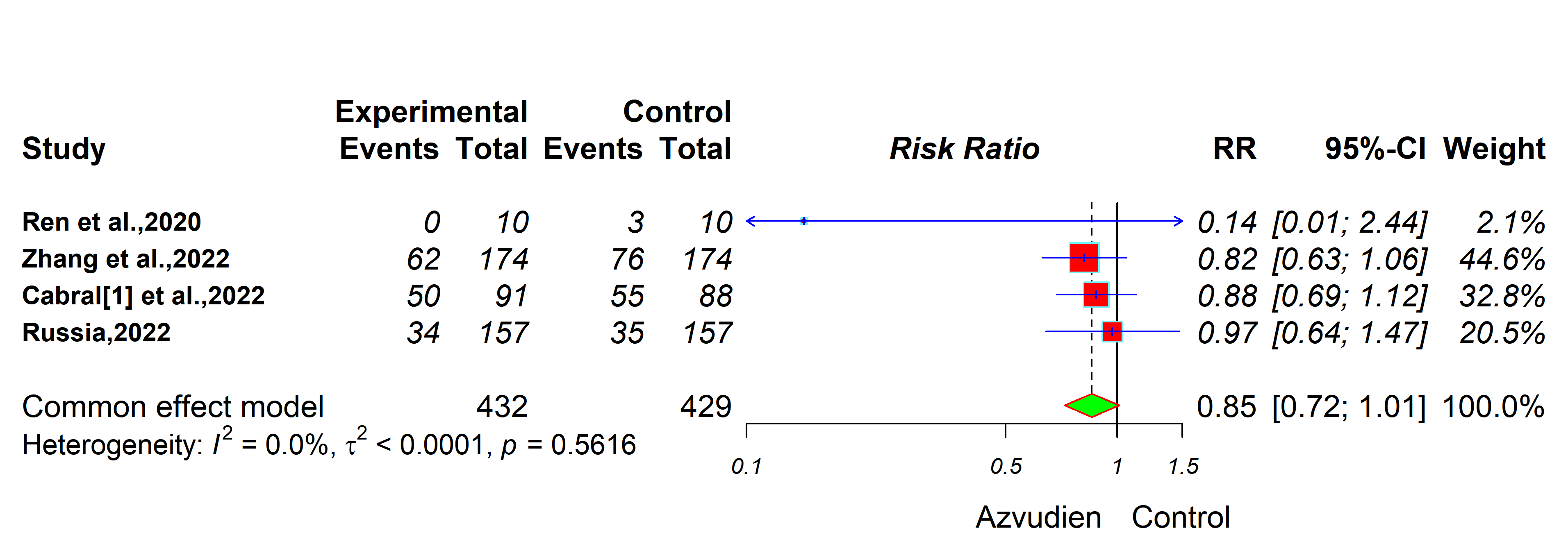


Figure S4. Adverse events (Removing Cabral[2] et al.,2022).


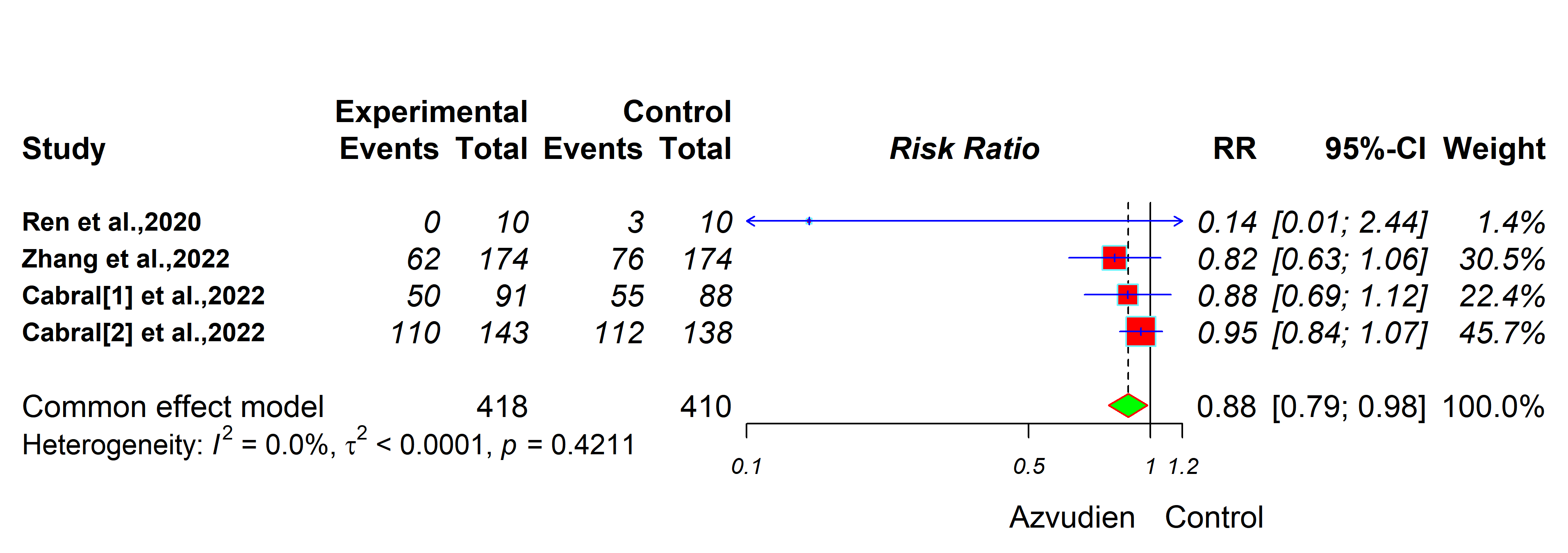


Figure S5. Adverse events (Removing Russia, 2022).

1. Serious adverse events in RCTs


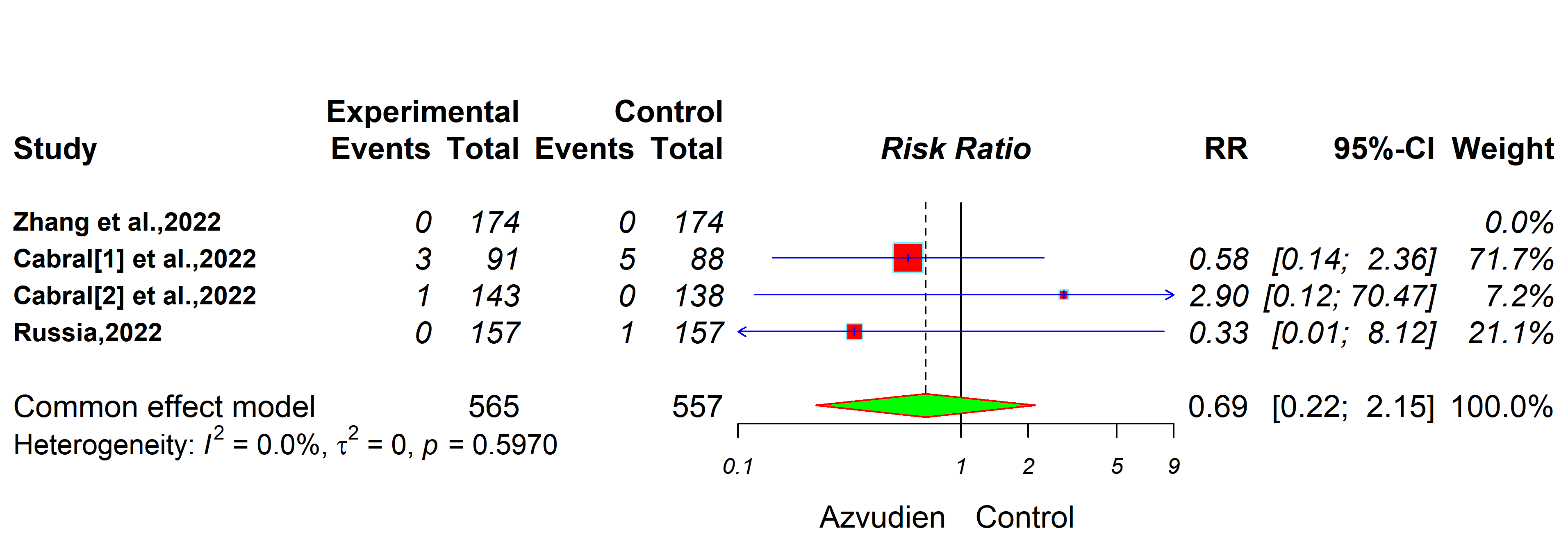


Figure S6. Serious adverse events (Removing Ren et al.,2020).


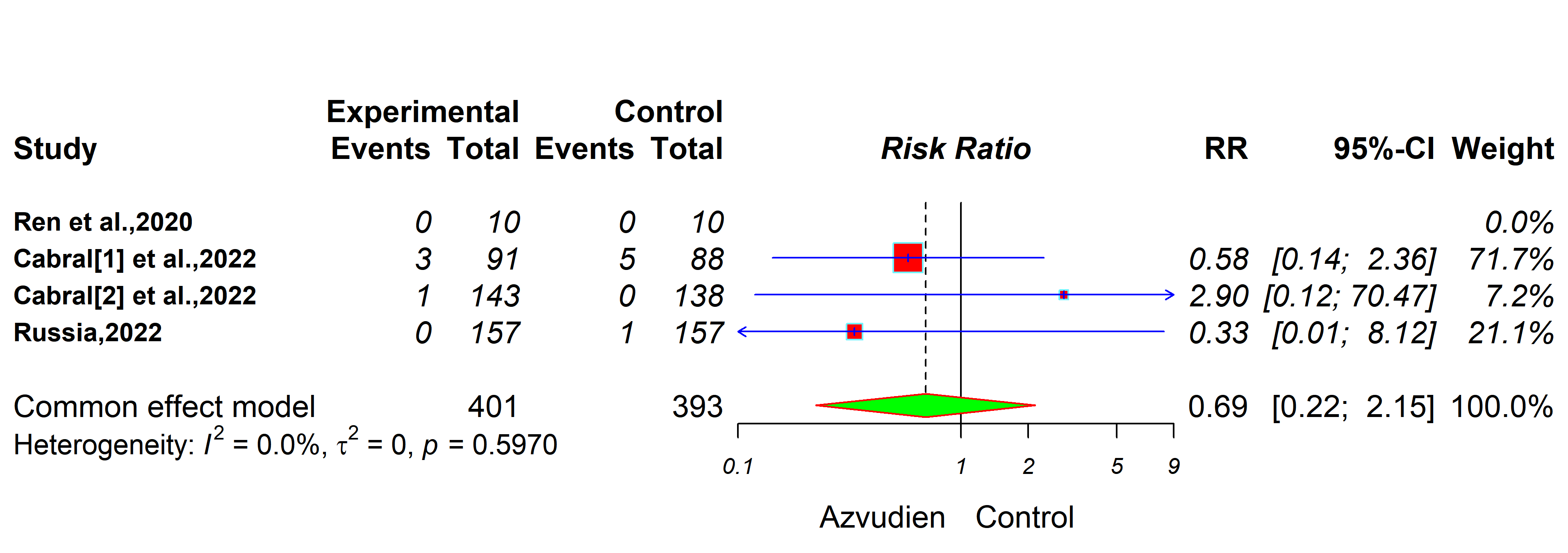


Figure S7. Serious adverse events (Removing Zhang et al.,2022).


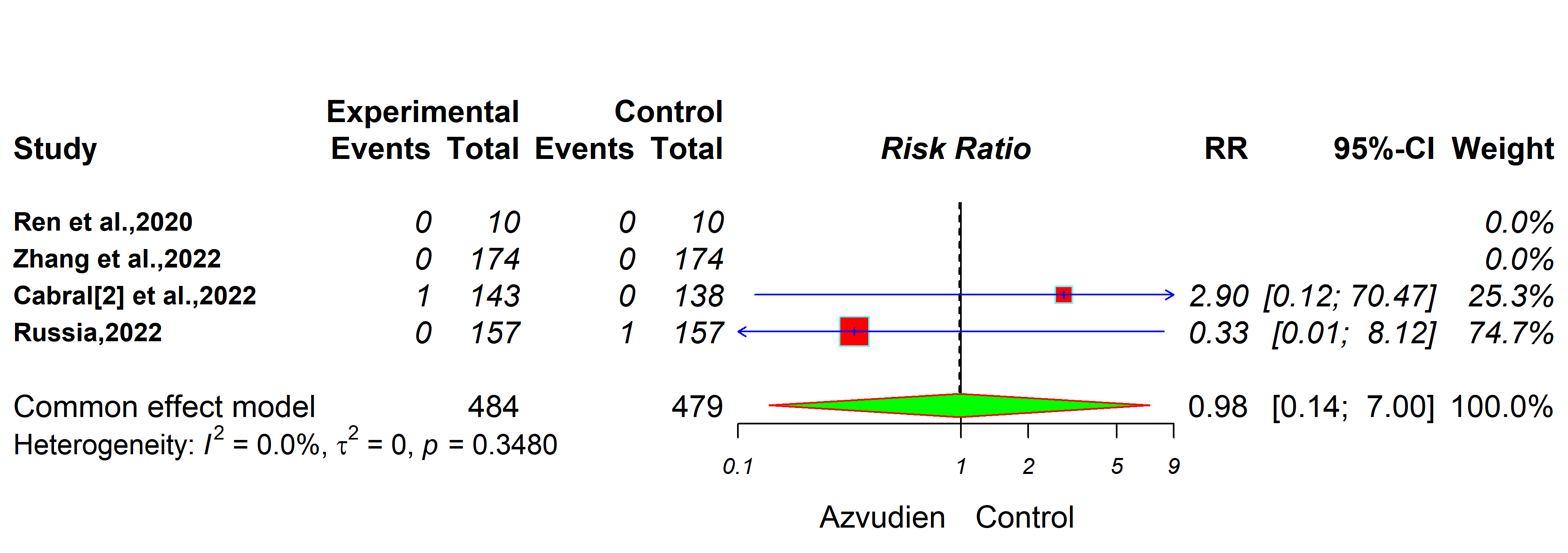


Figure S8. Serious adverse events (Removing Cabral[1] et al.,2022).


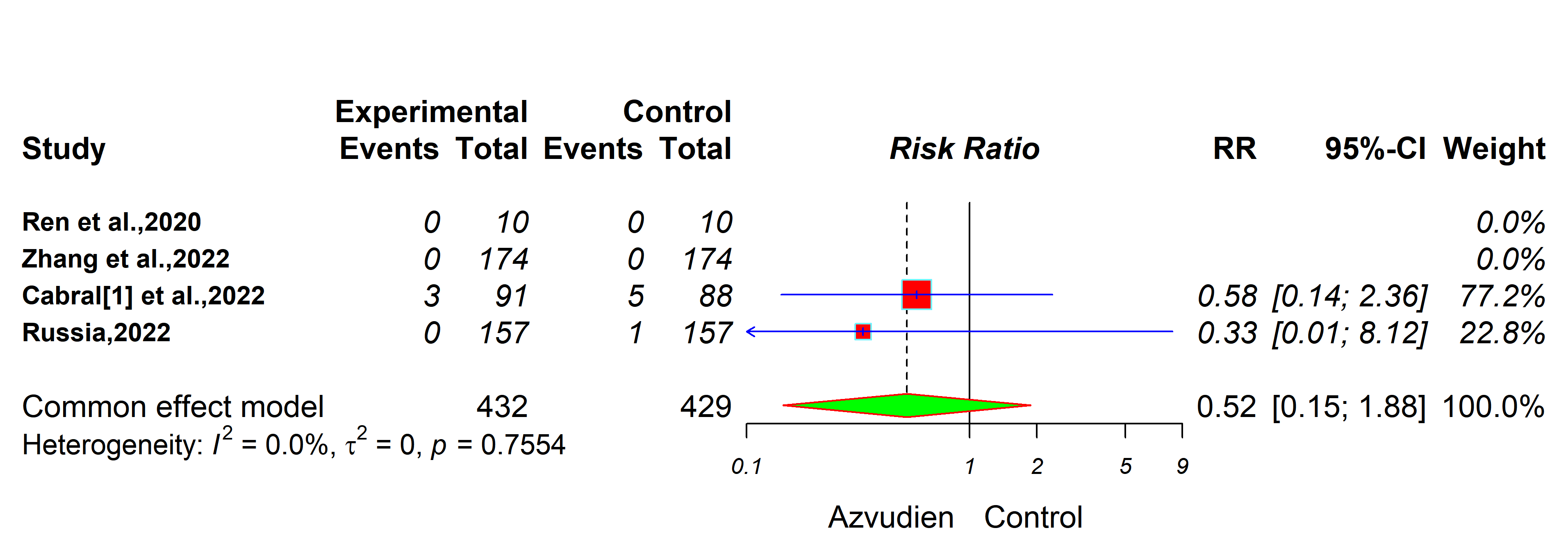


Figure S9. Serious adverse events (Removing Cabral[2] et al.,2022).


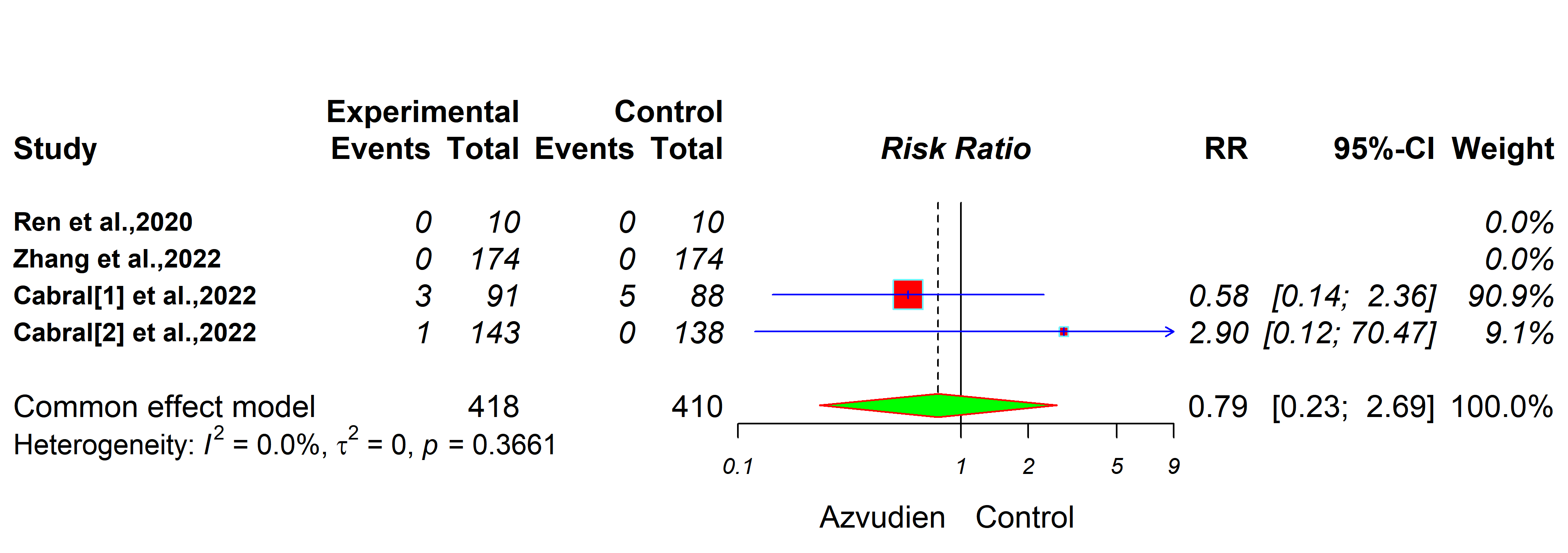


Figure S10. Serious adverse events (Removing Russia, 2022).

3. All cause mortality in Retrospective cohort studies


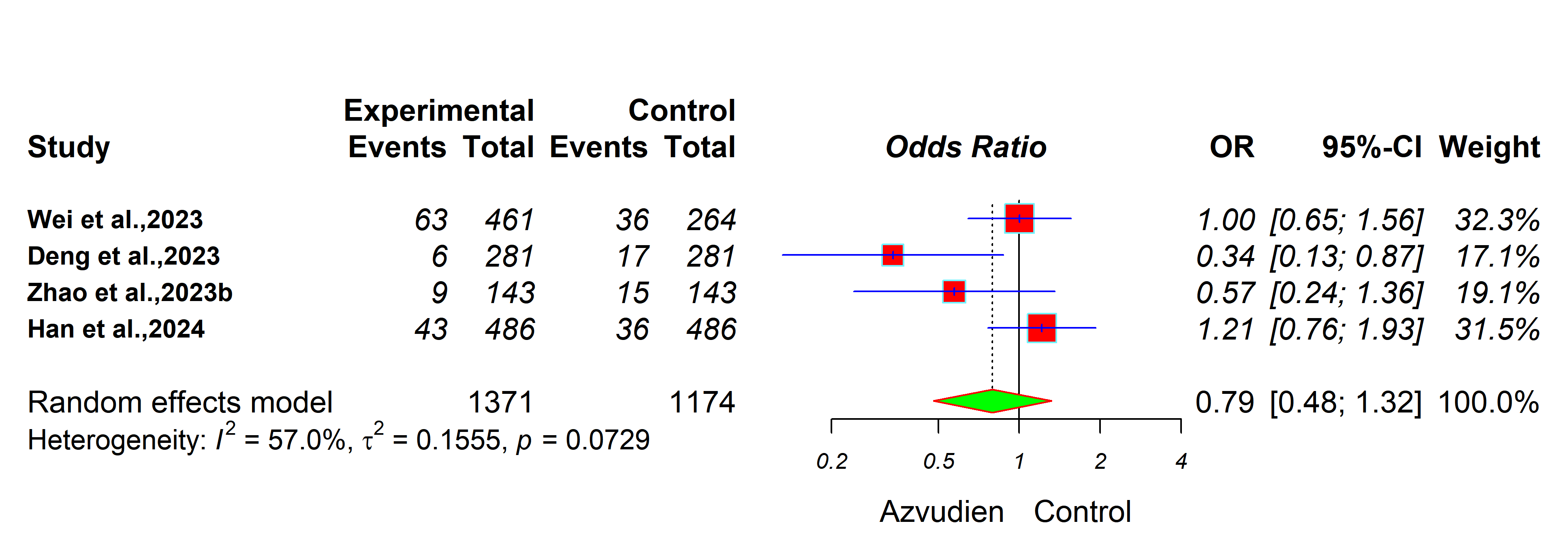


Figure S11. All cause mortality (Removing Zhao et al.,2023a).


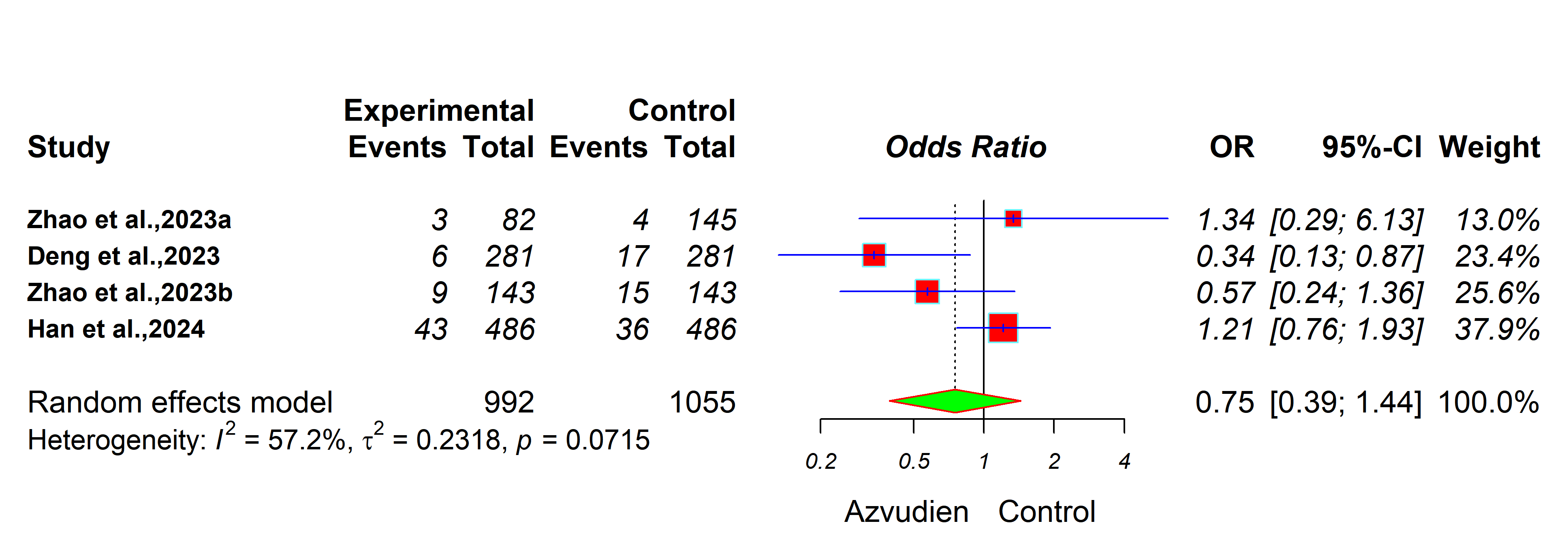


Figure S12. All cause mortality (Removing Wei et al.,2023).


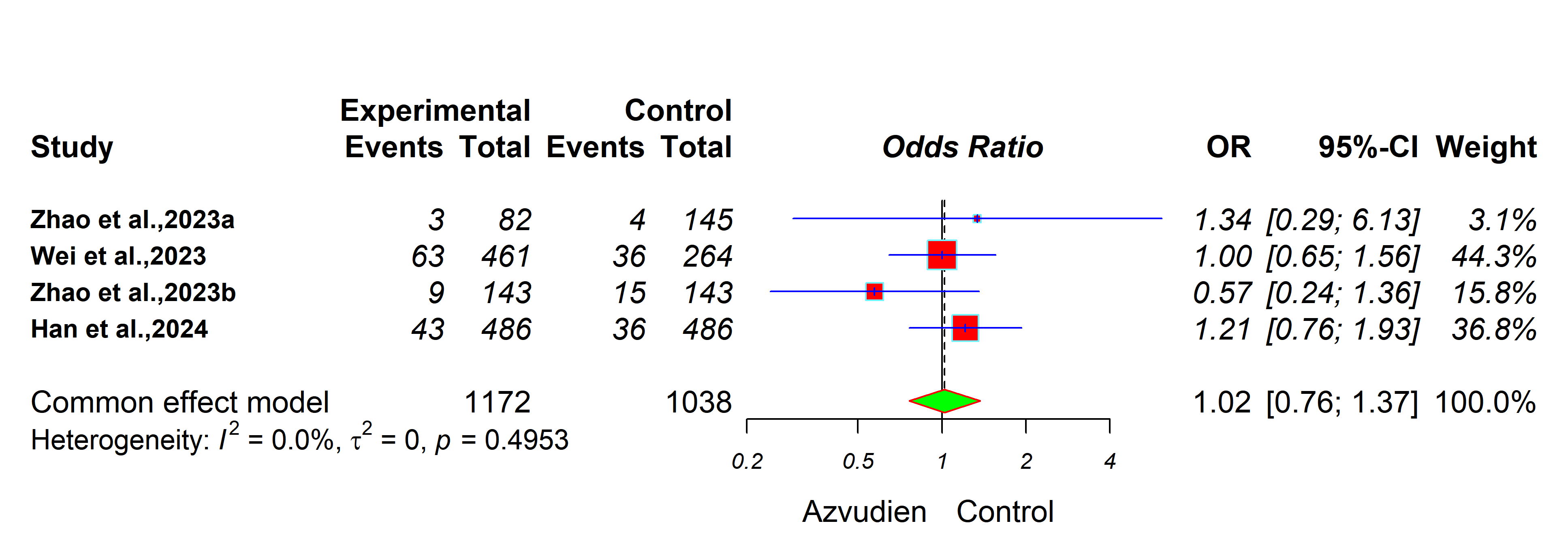


Figure S13. All cause mortality (Removing Deng et al.,2023).


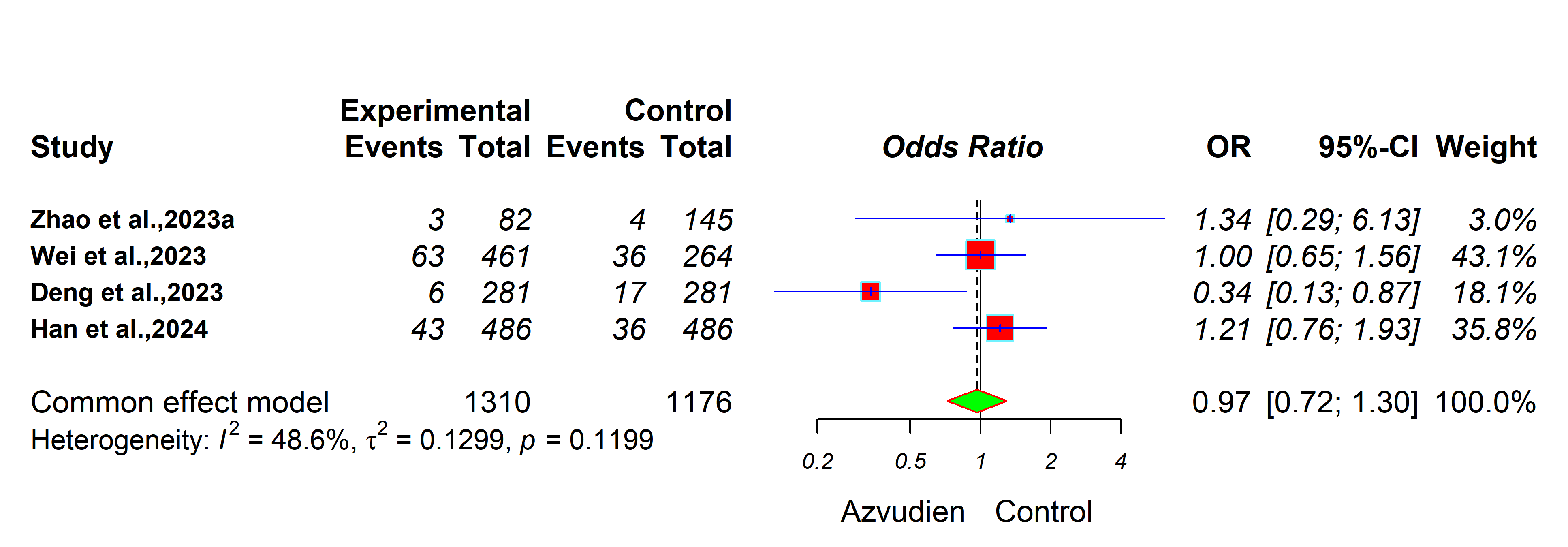


Figure S14. All cause mortality (Removing Zhao et al.,2023b).


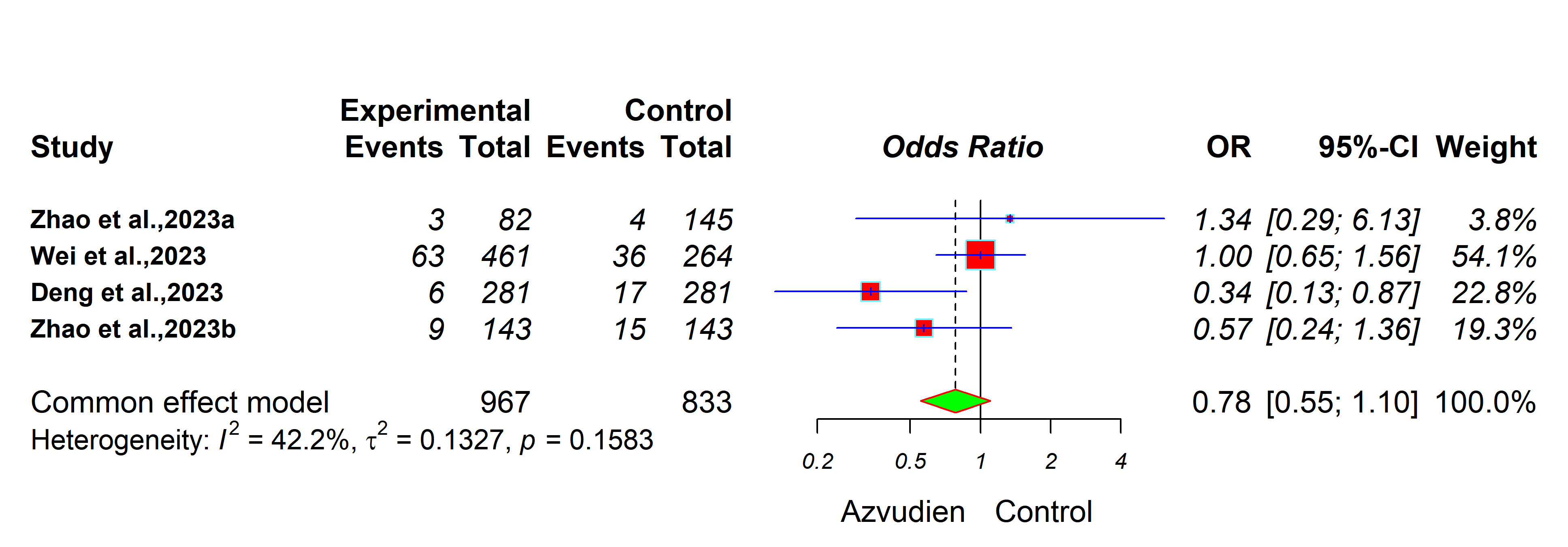


Figure S15. All cause mortality (Removing Han et al.,2024).


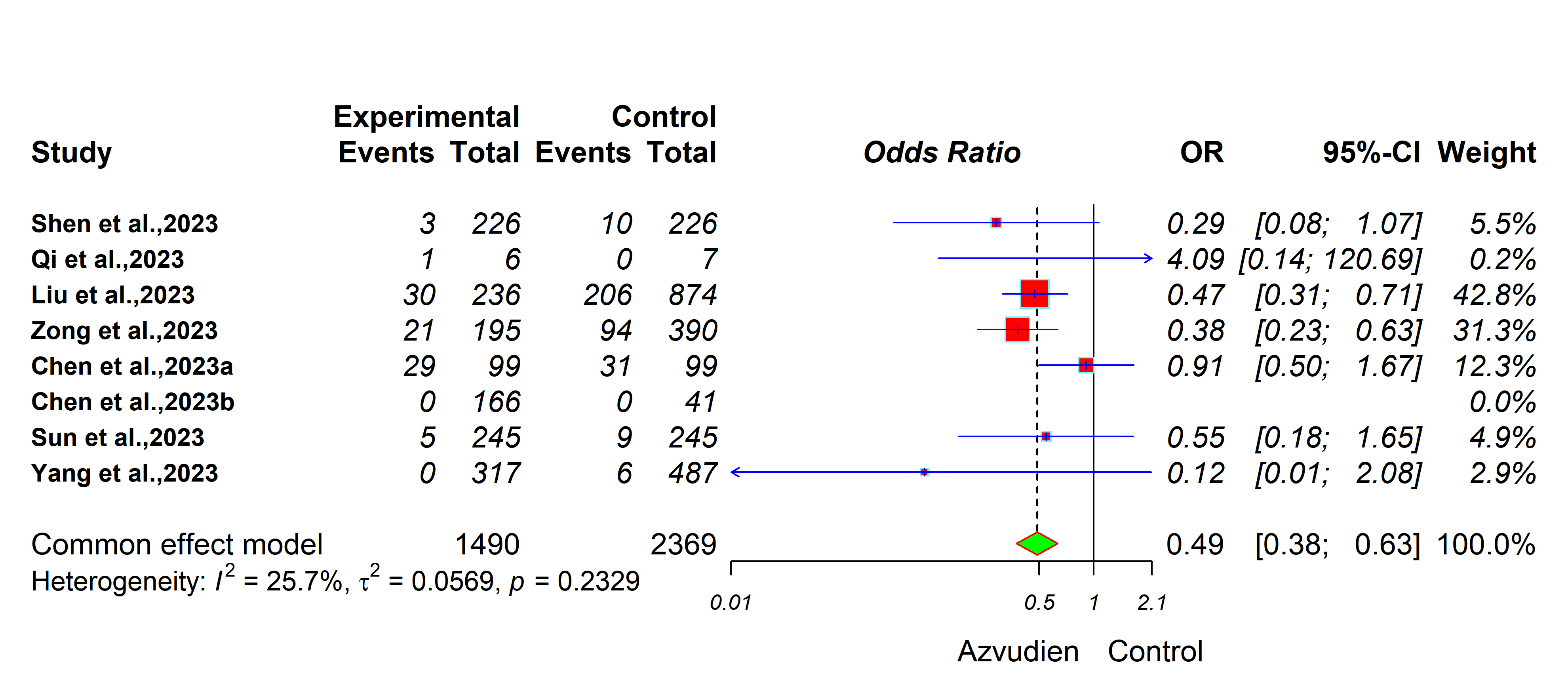


Figure S16. All cause mortality (Removing Shang et al.,2023).


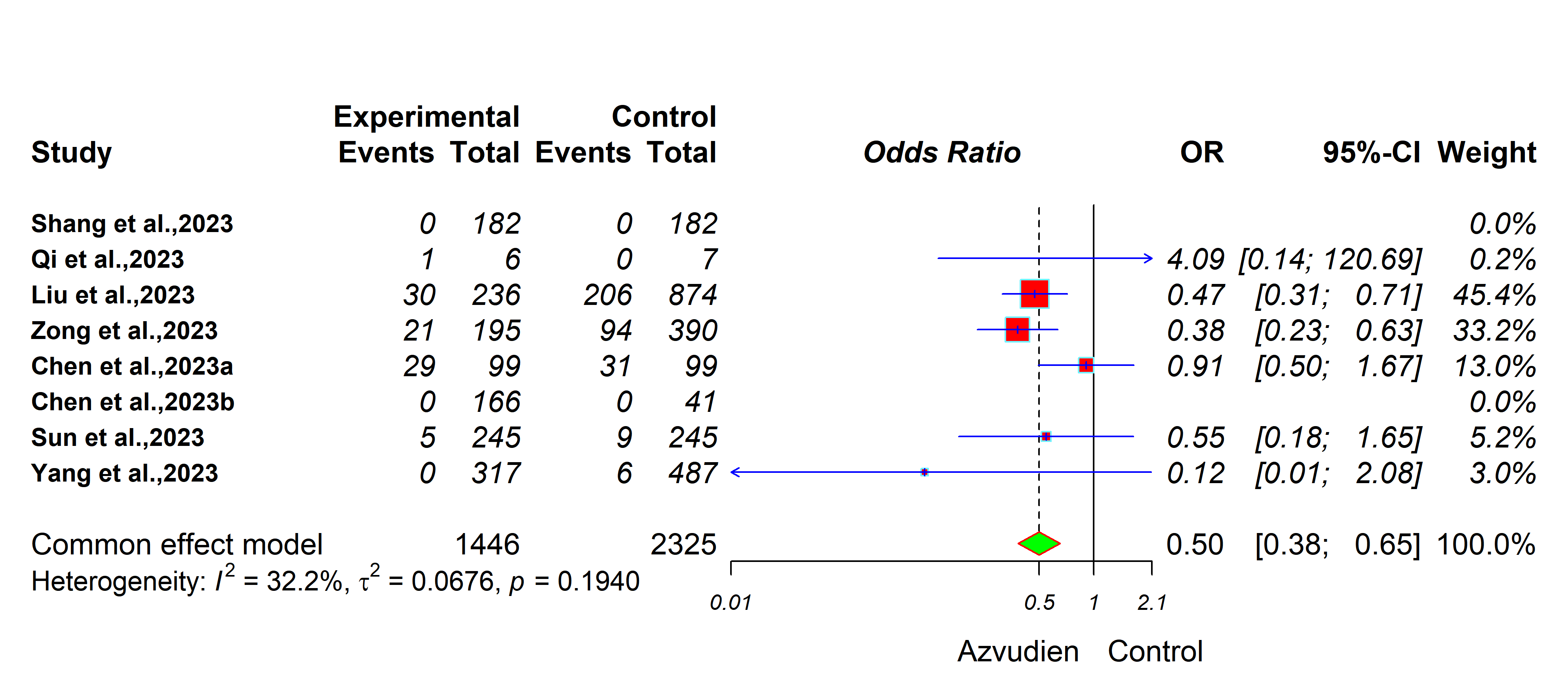


Figure S17. All cause mortality (Removing Shen et al.,2023).


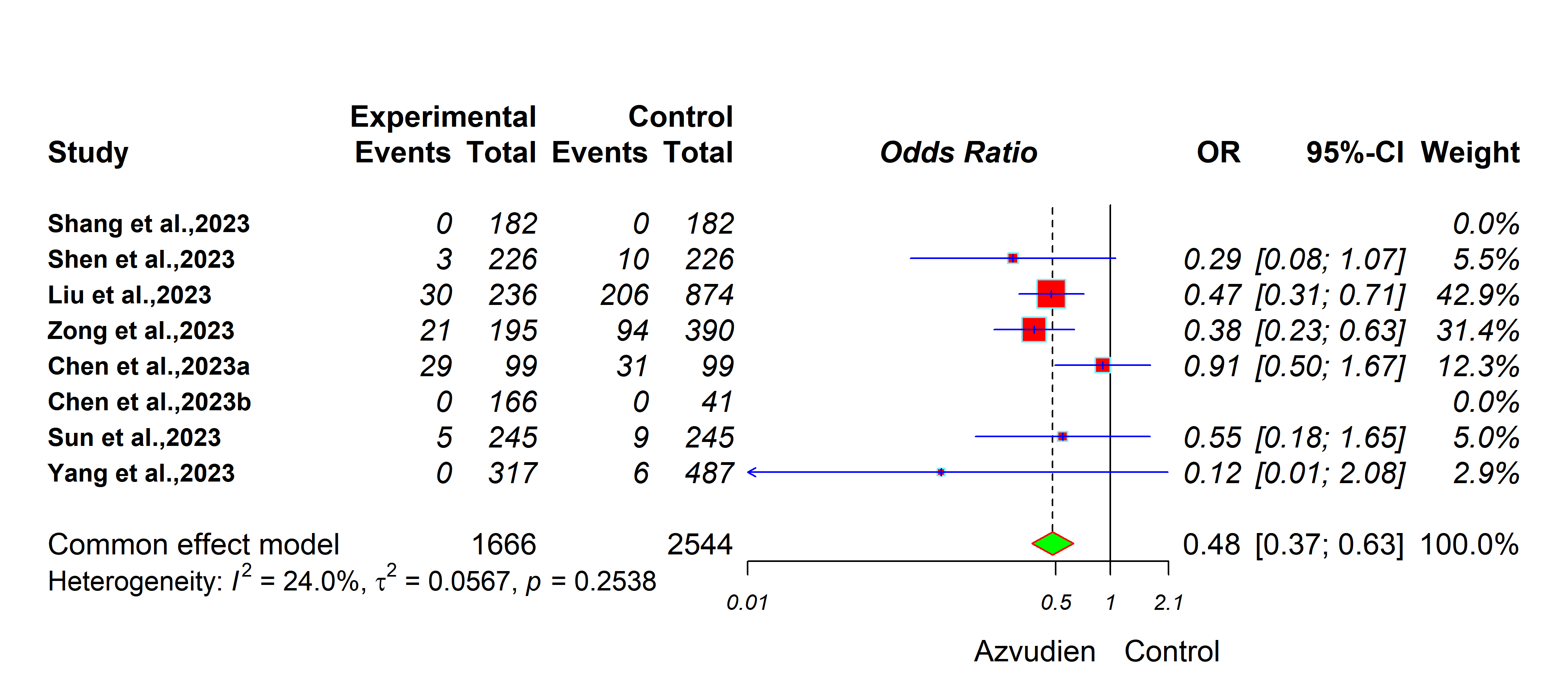


Figure S18. All cause mortality (Removing Qi et al.,2023).


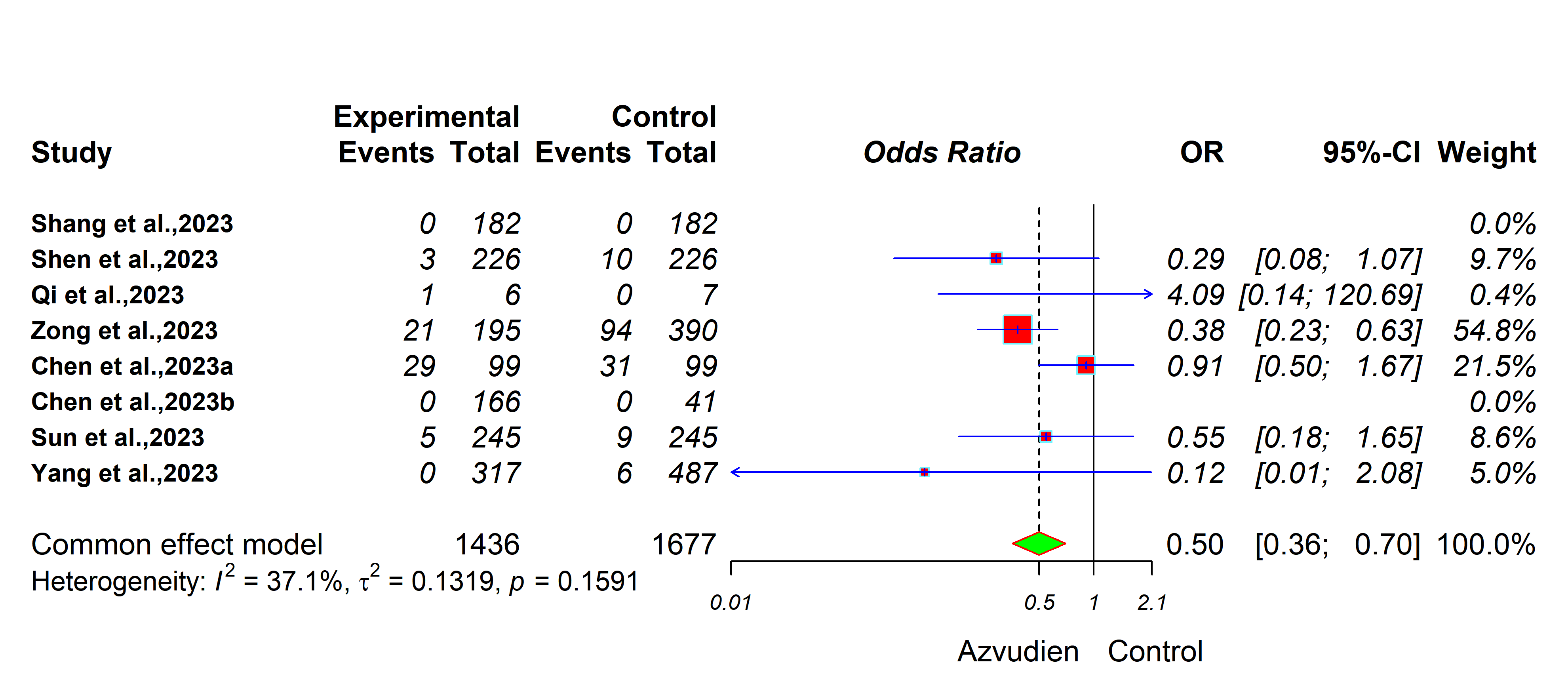


Figure S19. All cause mortality (Removing Liu et al.,2023).


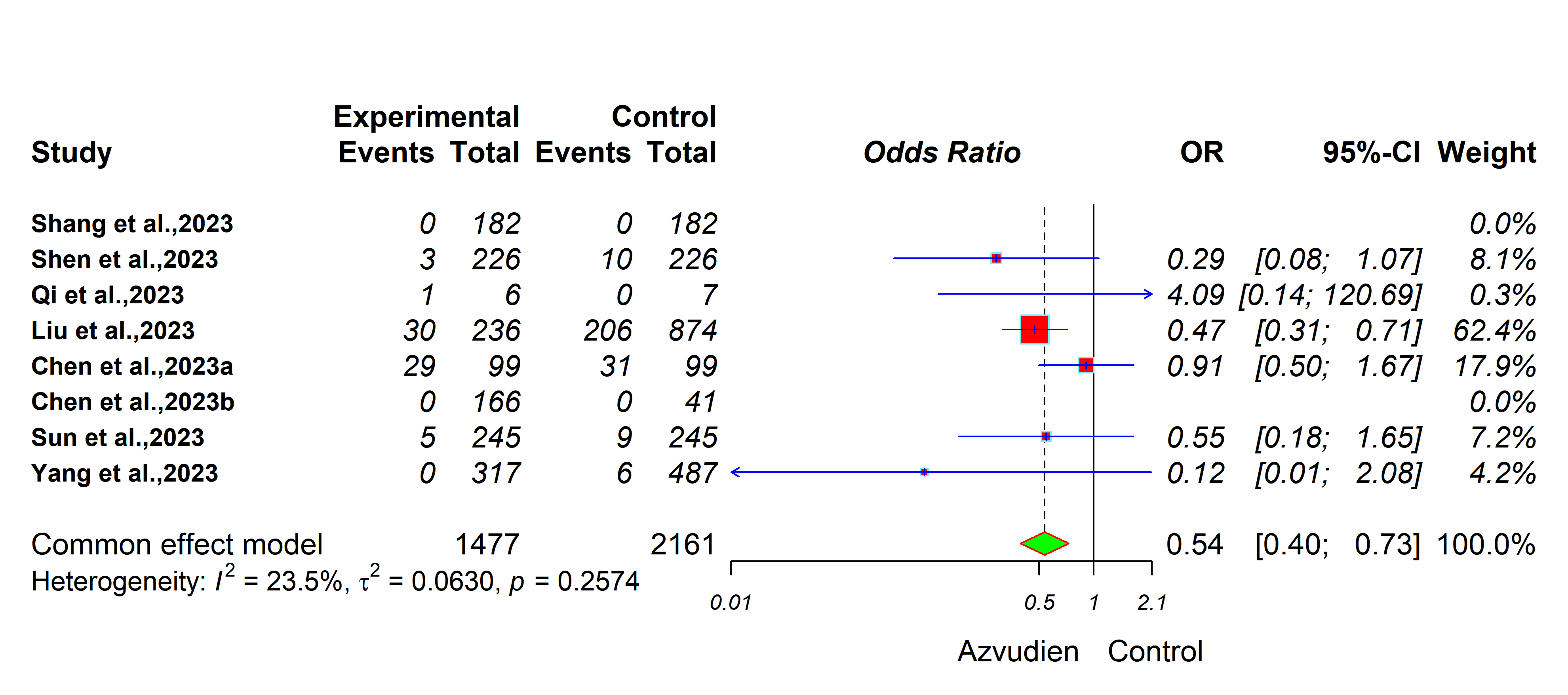


Figure S20. All cause mortality (Removing Zong et al.,2023).


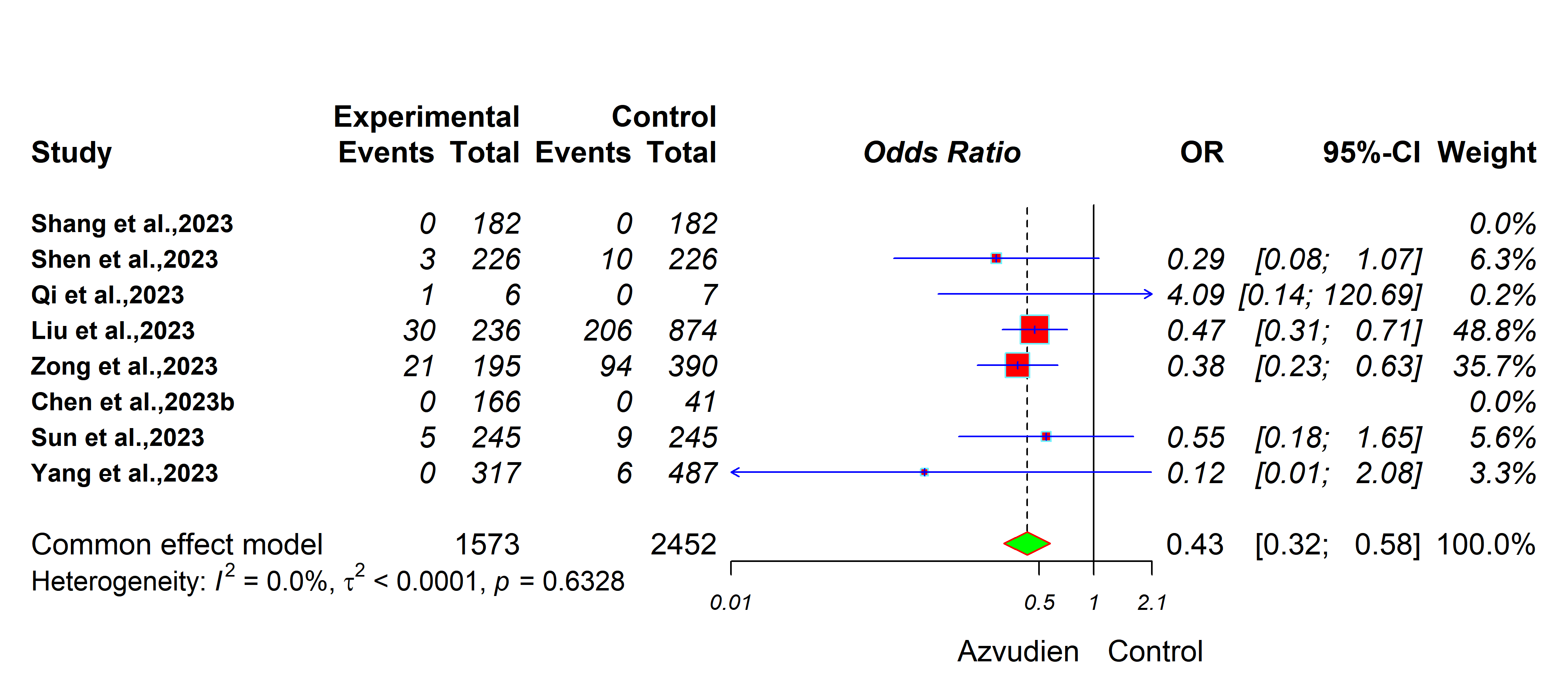


Figure S21. All cause mortality (Removing Chen et al.,2023a).


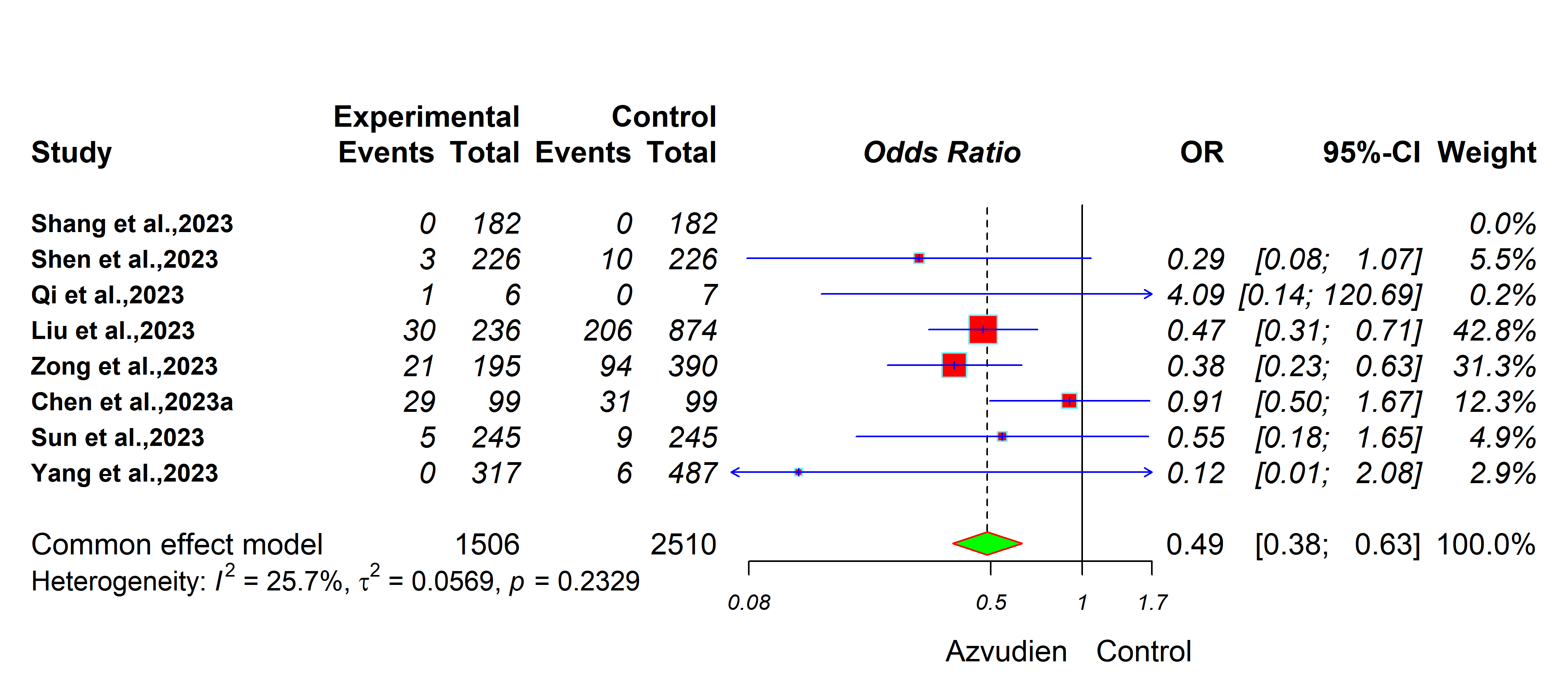


Figure S22. All cause mortality (Removing Chen et al.,2023b).


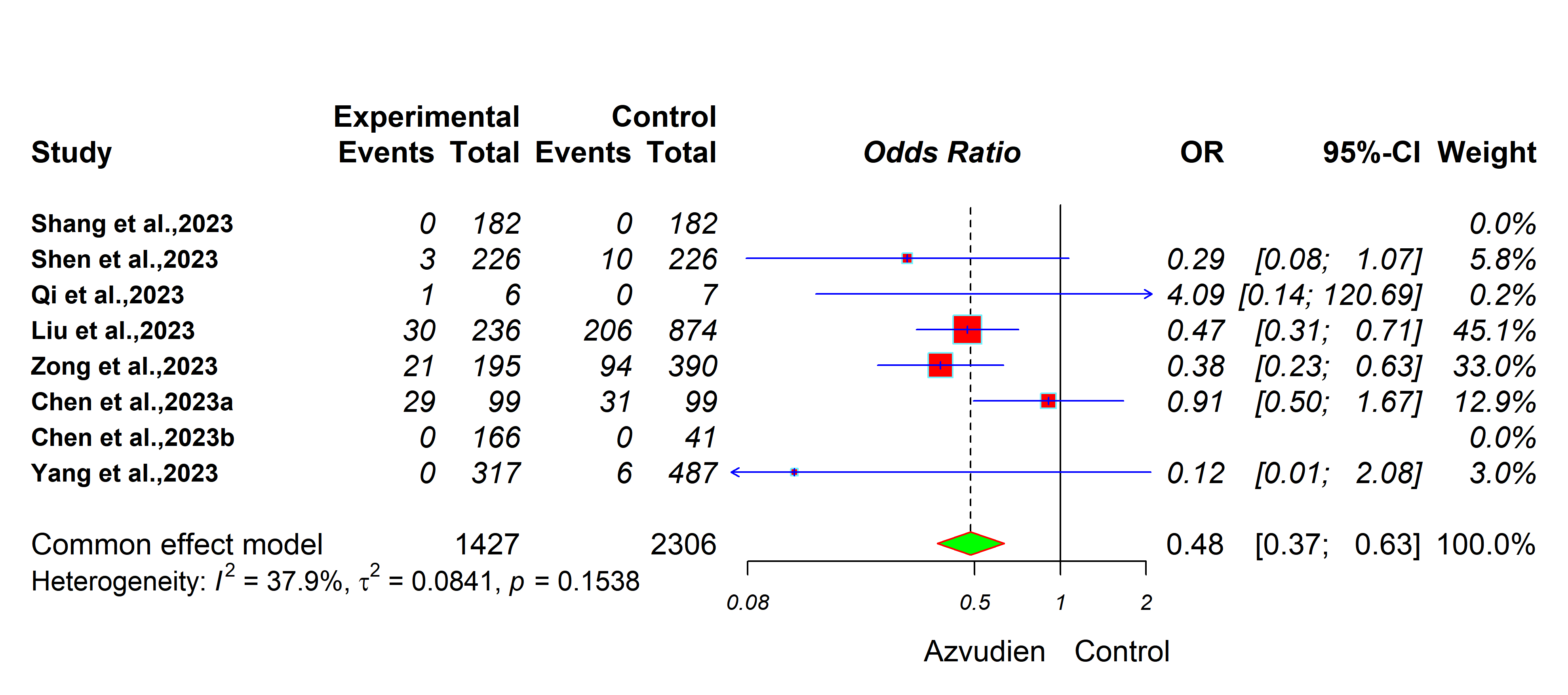


Figure S23. All cause mortality (Removing Sun et al.,2023).


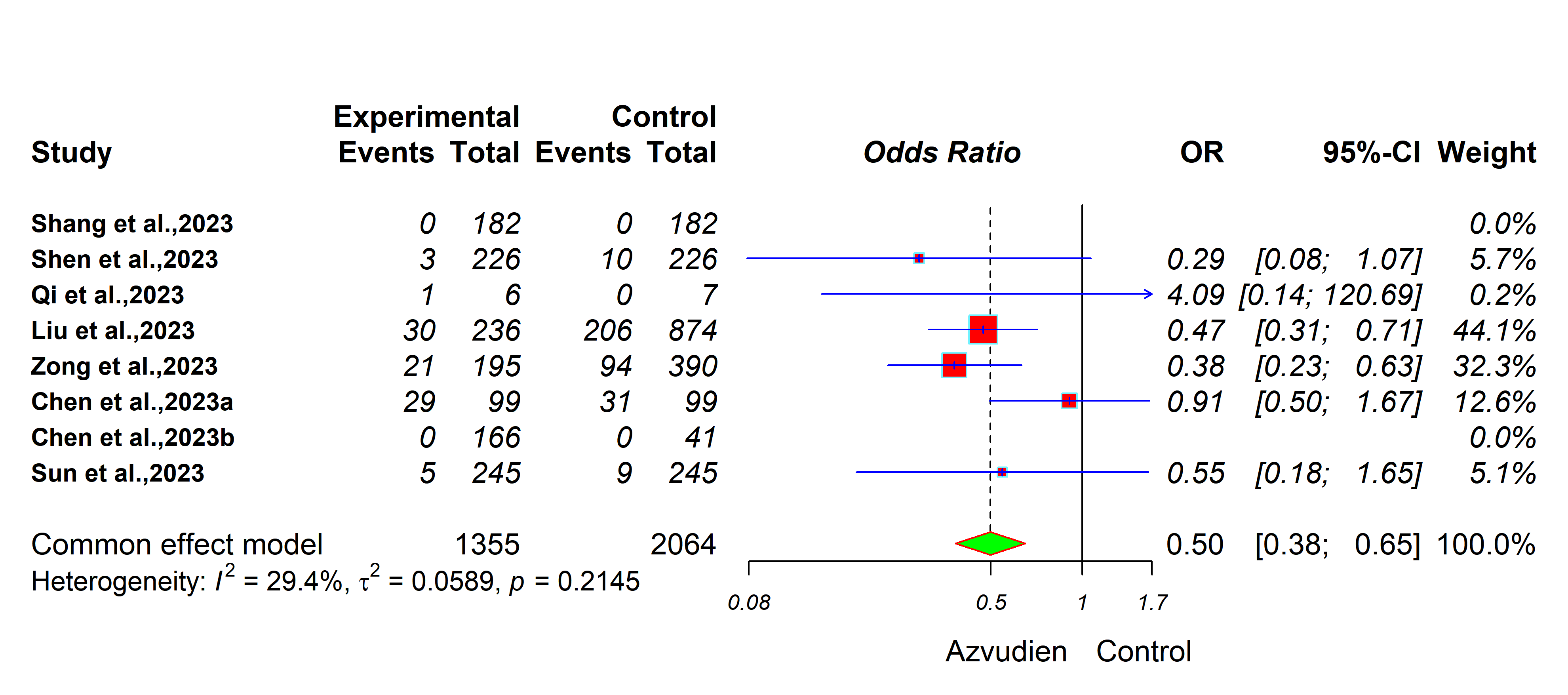


Figure S24. All cause mortality (Removing Yang et al.,2023).

4. Composite disease progression in Retrospective cohort studies


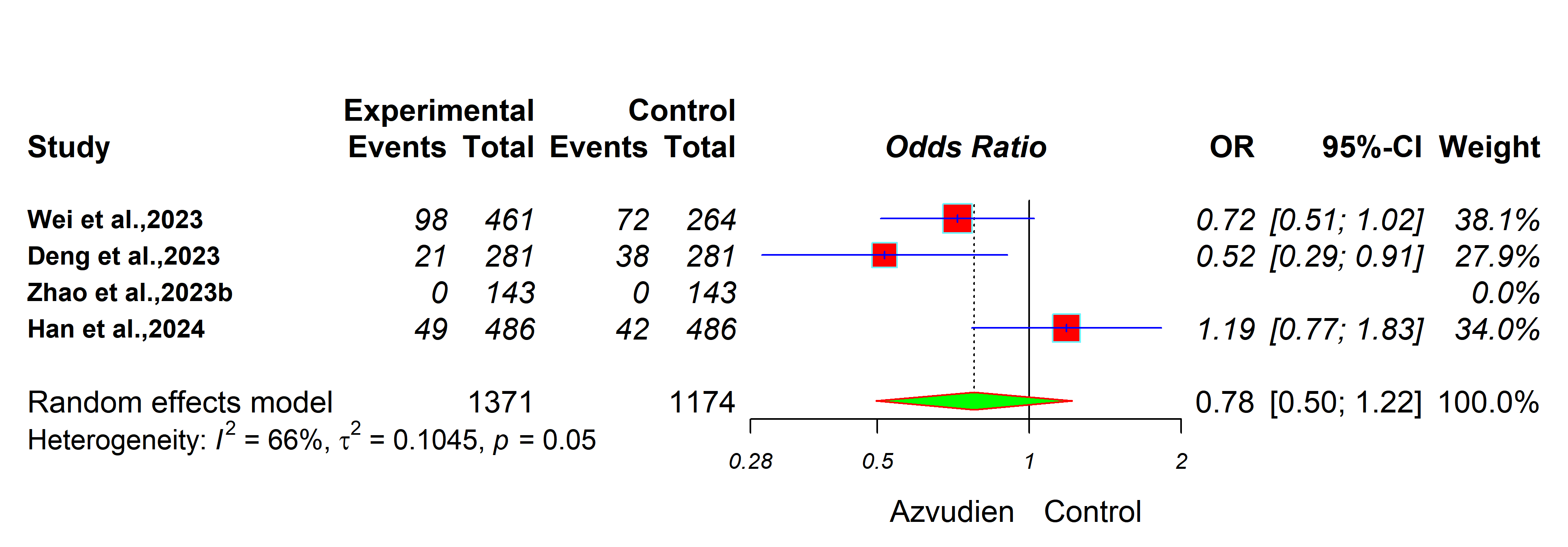


Figure S25. Composite disease progression (Removing Zhao et al.,2023a).


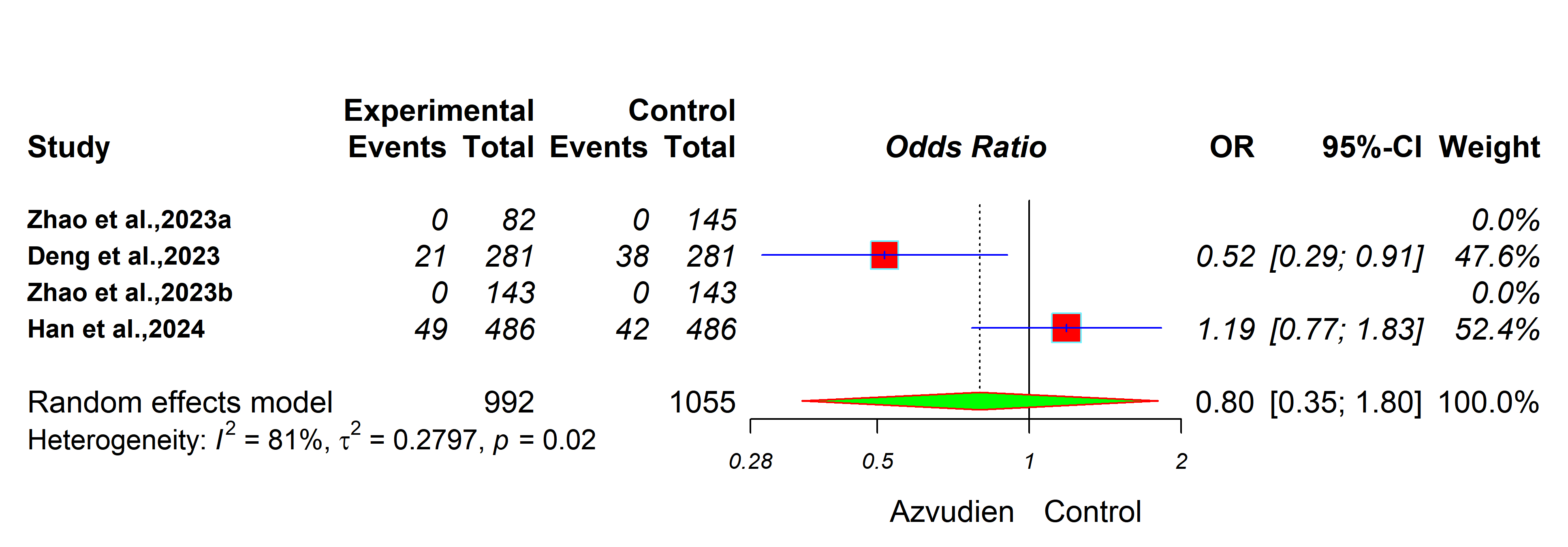


Figure S26. Composite disease progression (Removing Wei et al.,2023).


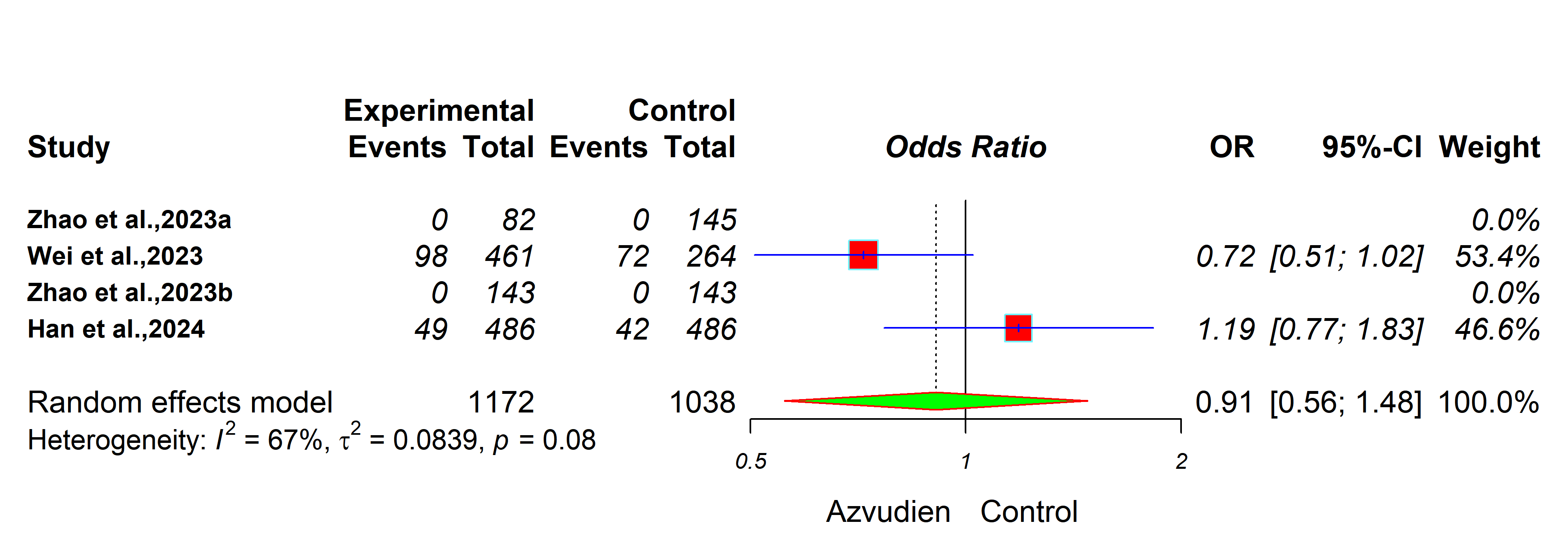


Figure S27. Composite disease progression (Removing Deng et al.,2023).


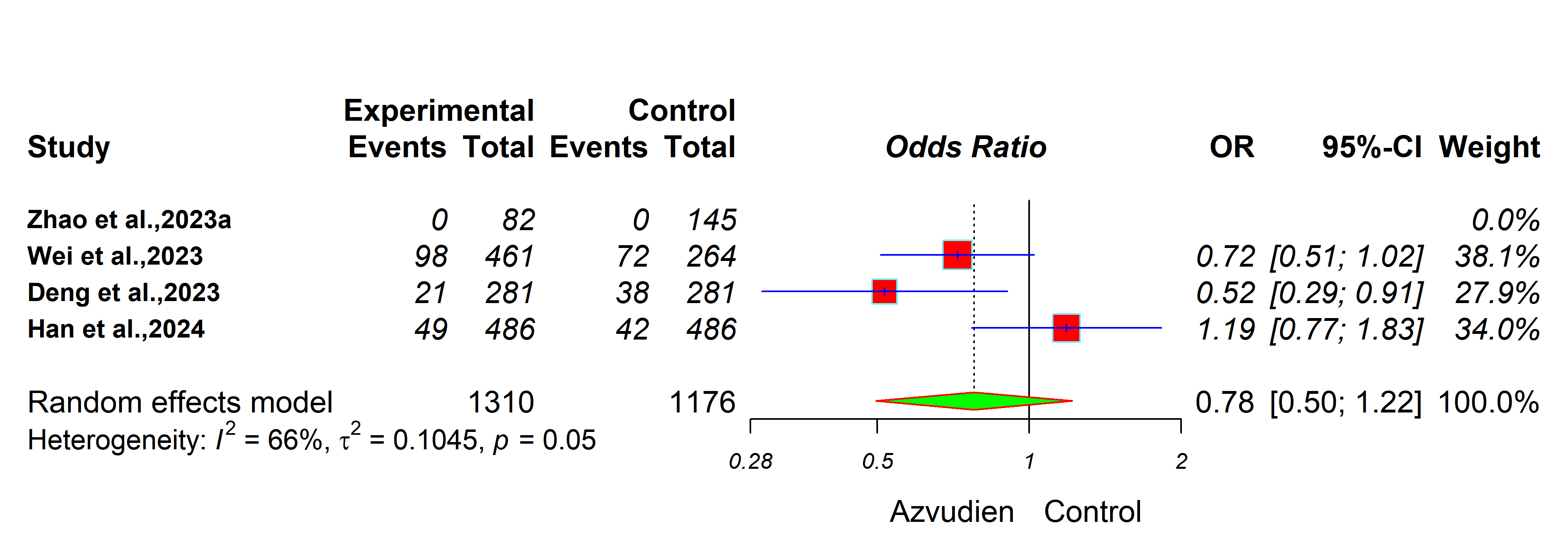


Figure S28. Composite disease progression (Removing Zhao et al.,2023b).


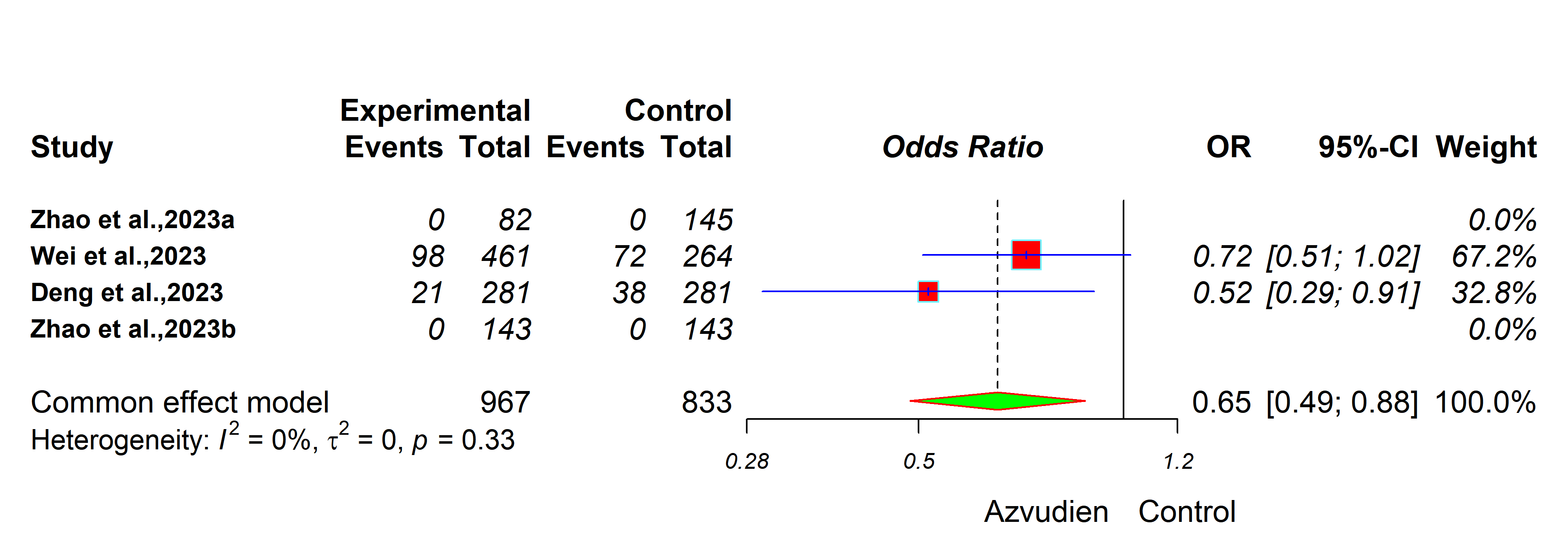


Figure S29. Composite disease progression (Removing Han et al.,2024).


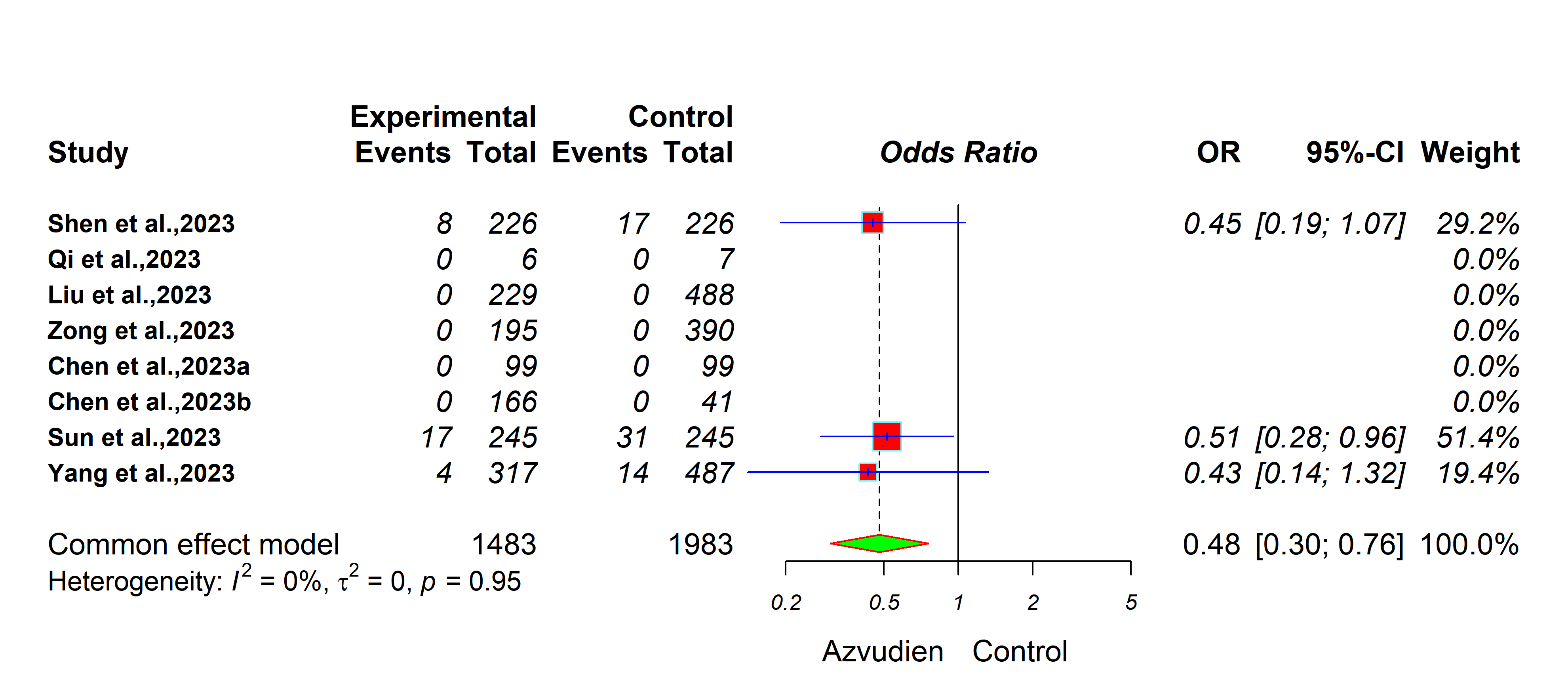


Figure S30. Composite disease progression (Removing Shang et al.,2023).


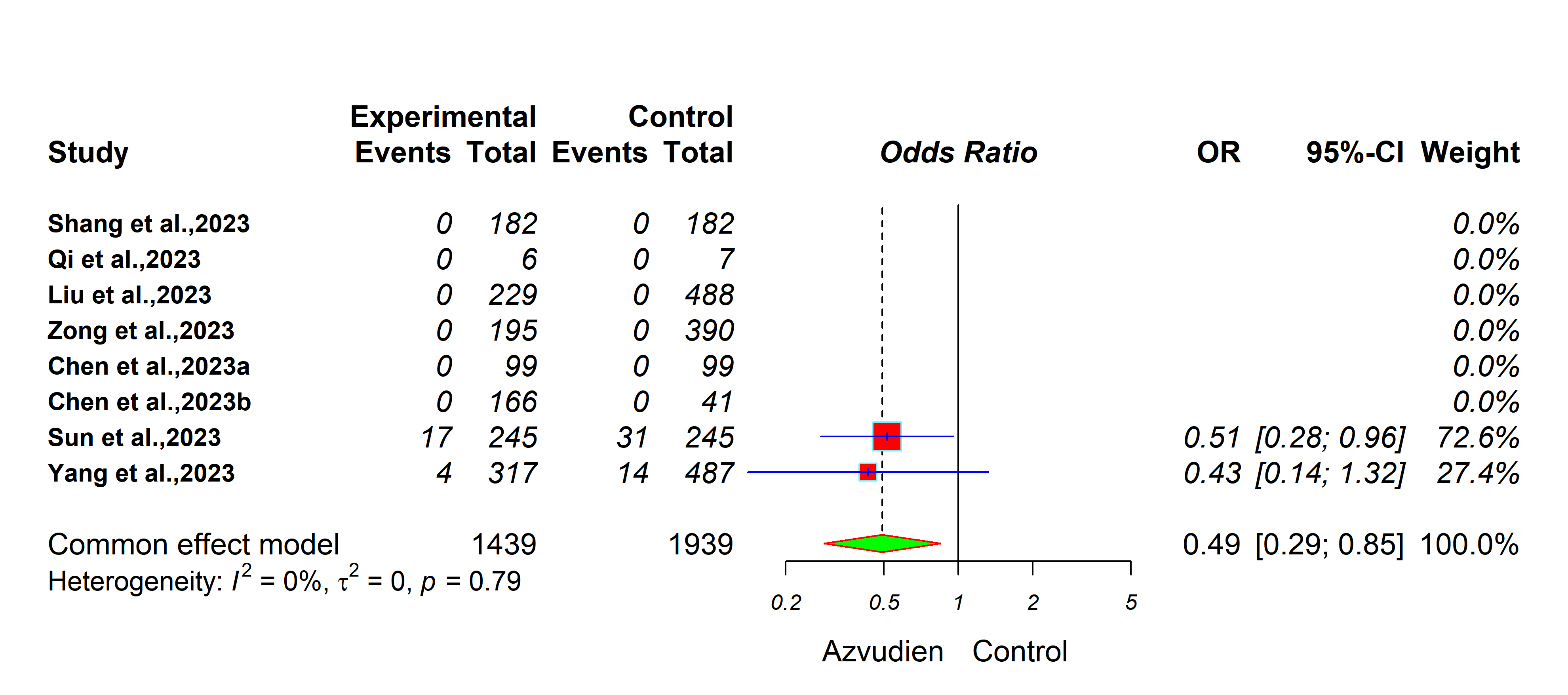


Figure S31. Composite disease progression (Removing Shen et al.,2023).


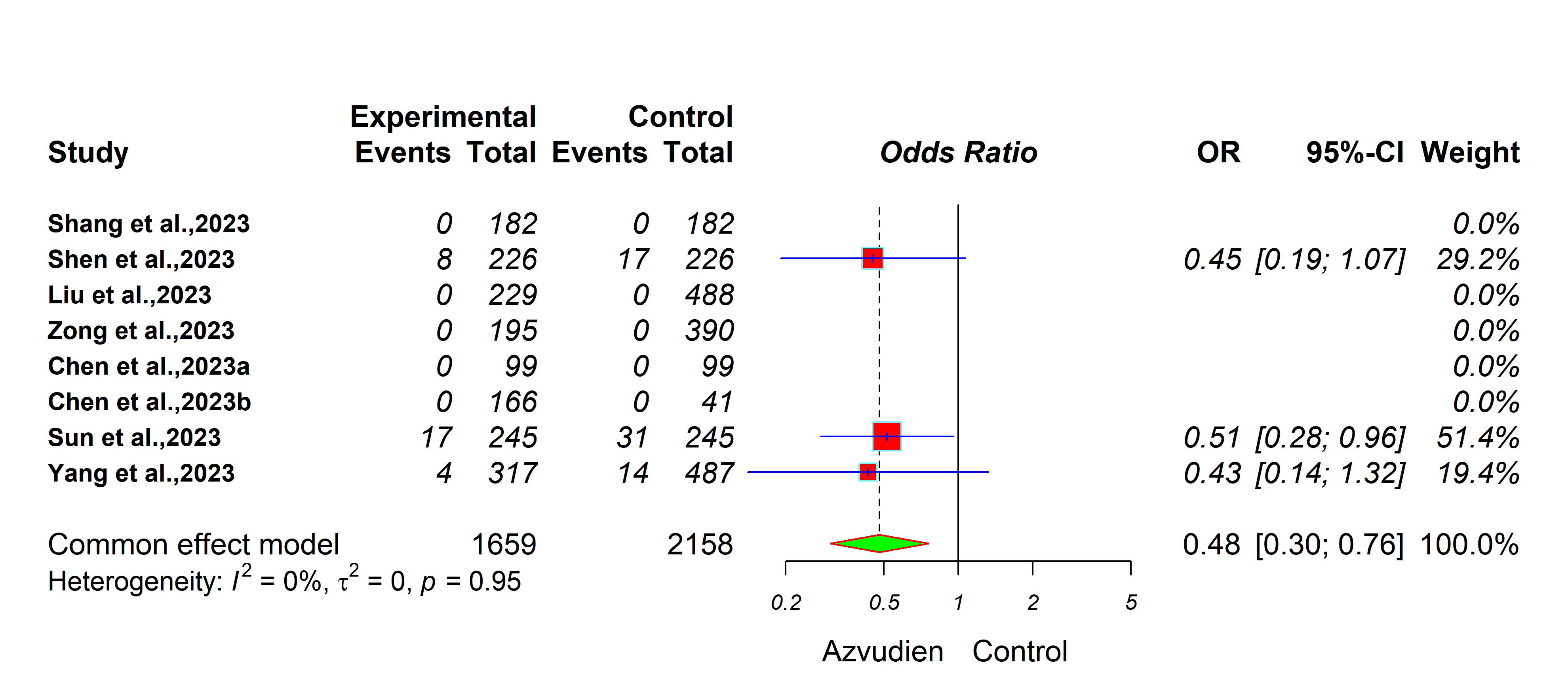


Figure S32. Composite disease progression (Removing Qi et al.,2023).


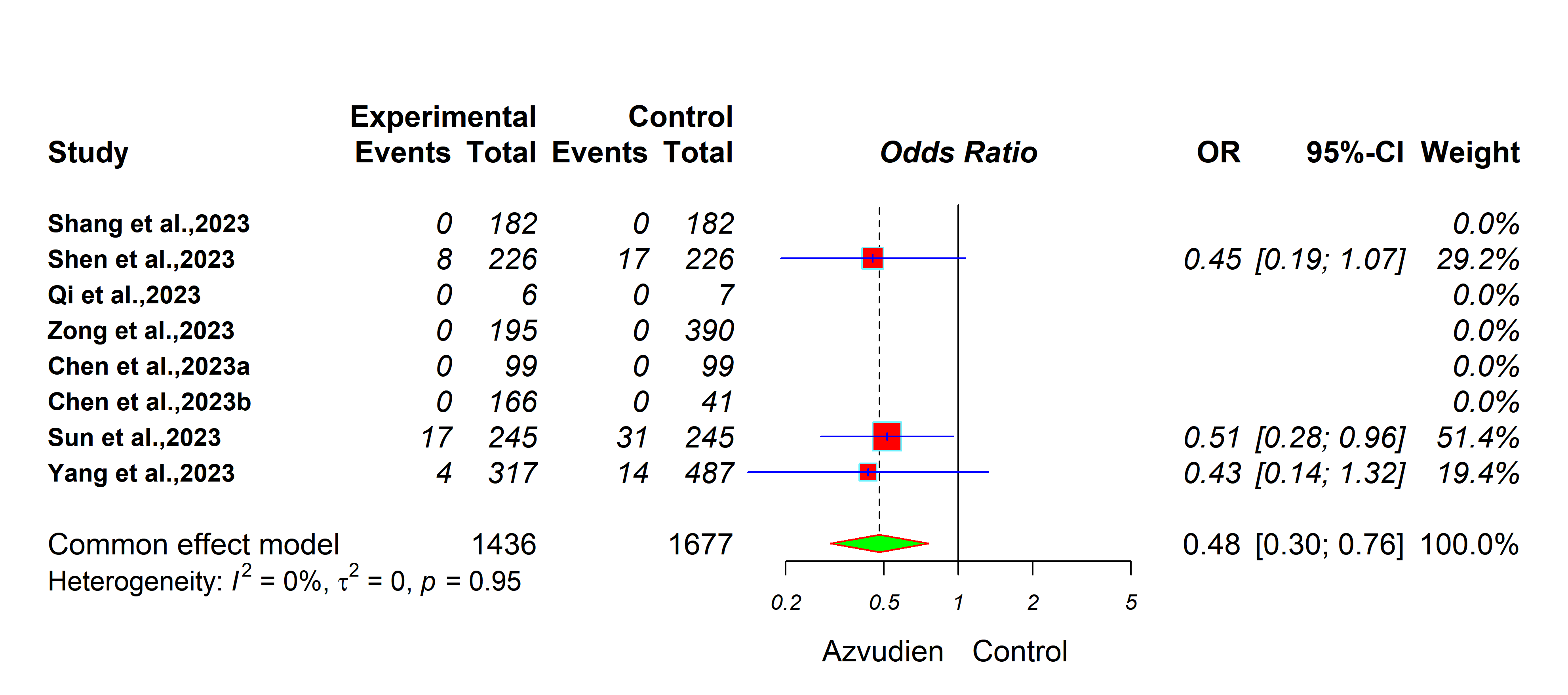


Figure S33. Composite disease progression (Removing Liu et al.,2023).


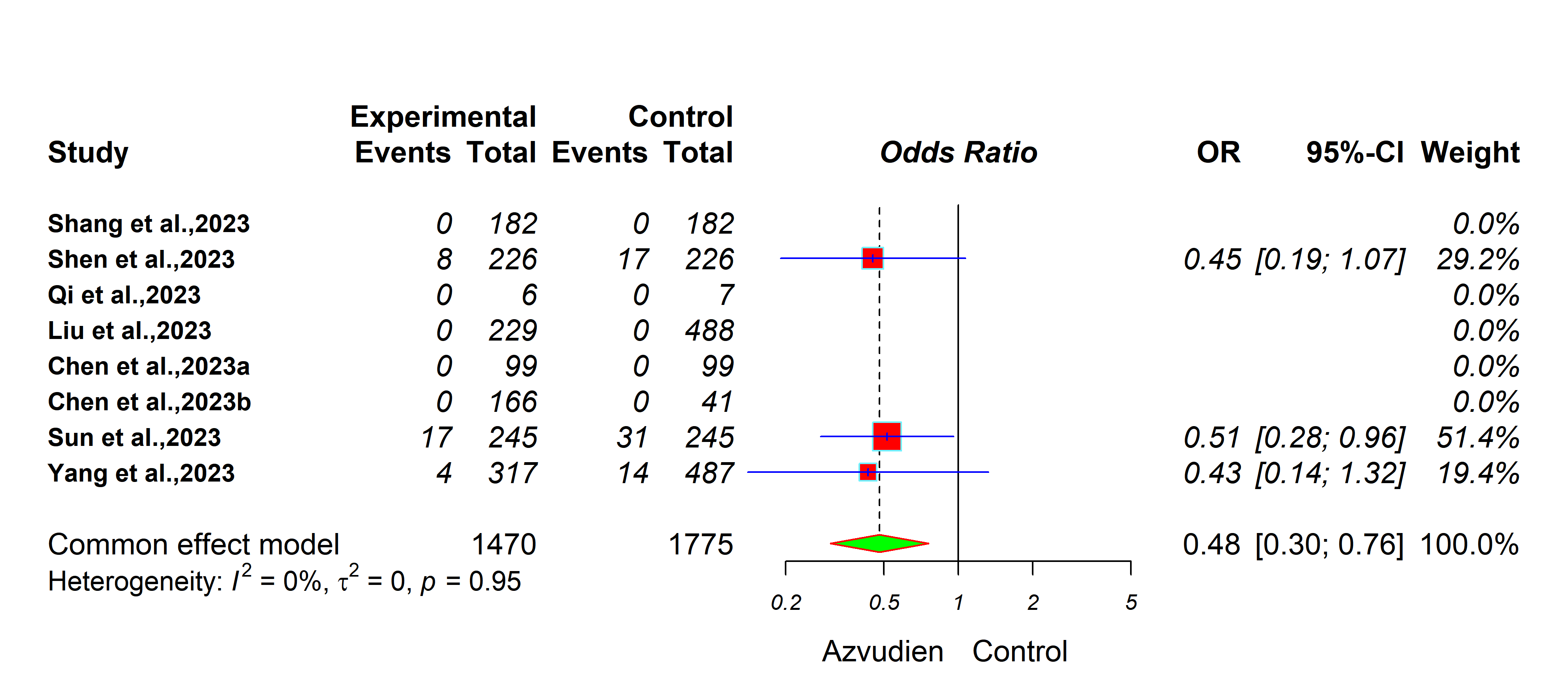


Figure S34. Composite disease progression (Removing Zong et al.,2023).


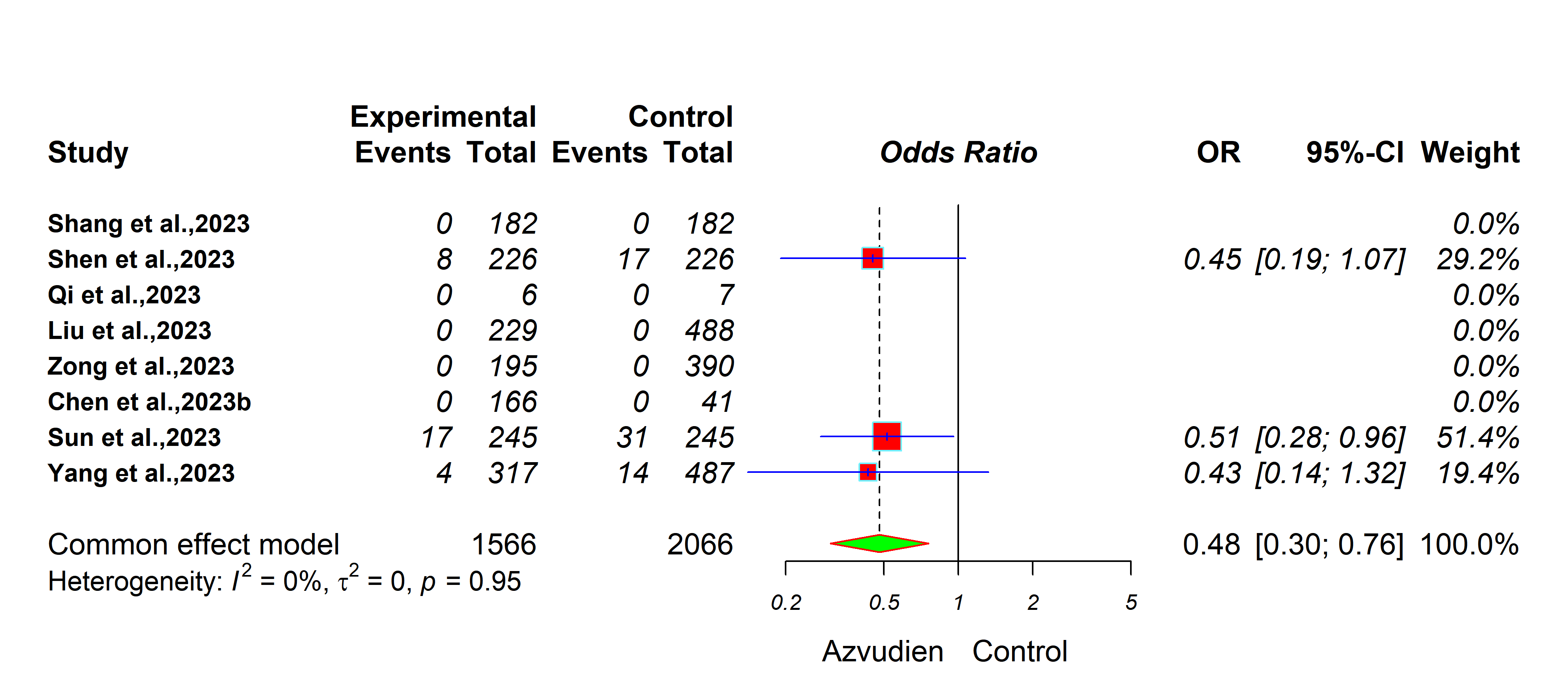


Figure S35. Composite disease progression (Removing Chen et al.,2023a).


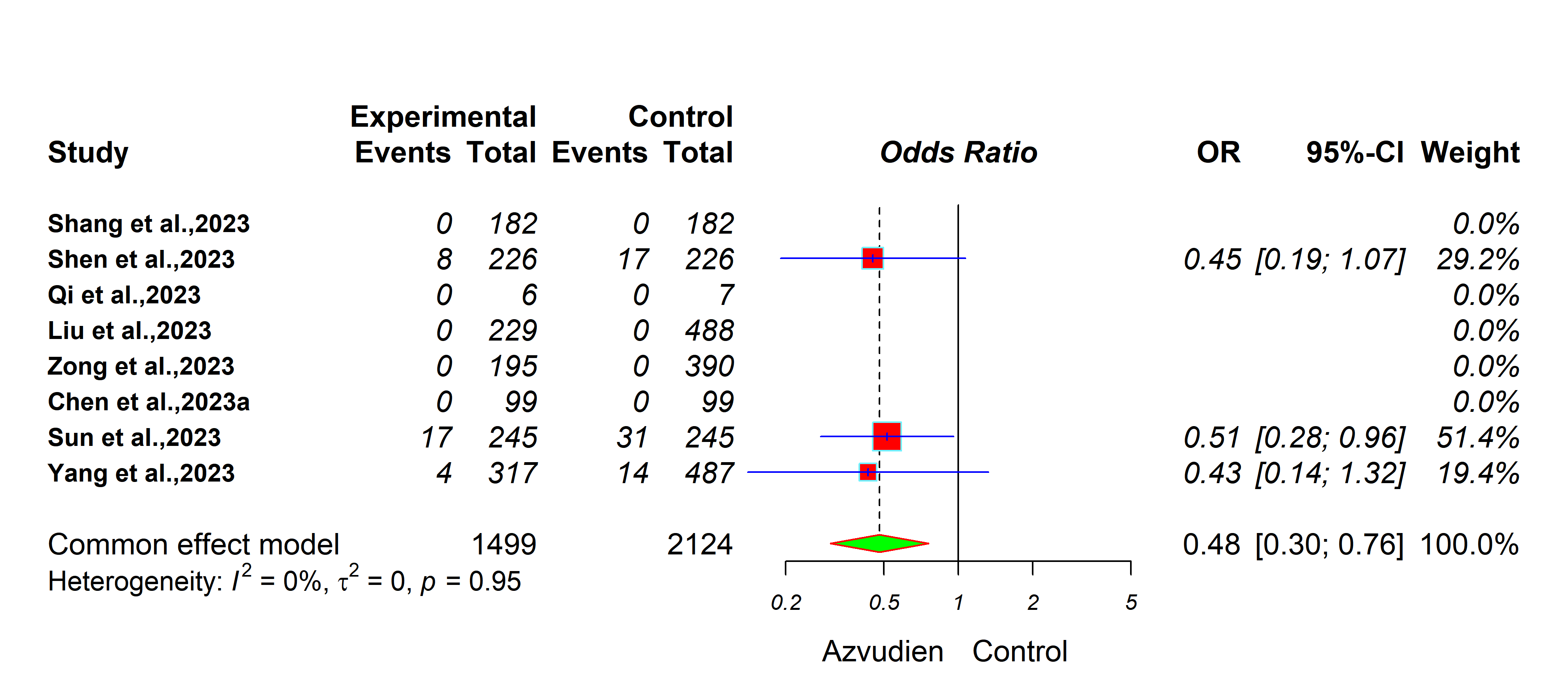


Figure S36. Composite disease progression (Removing Chen et al.,2023b).


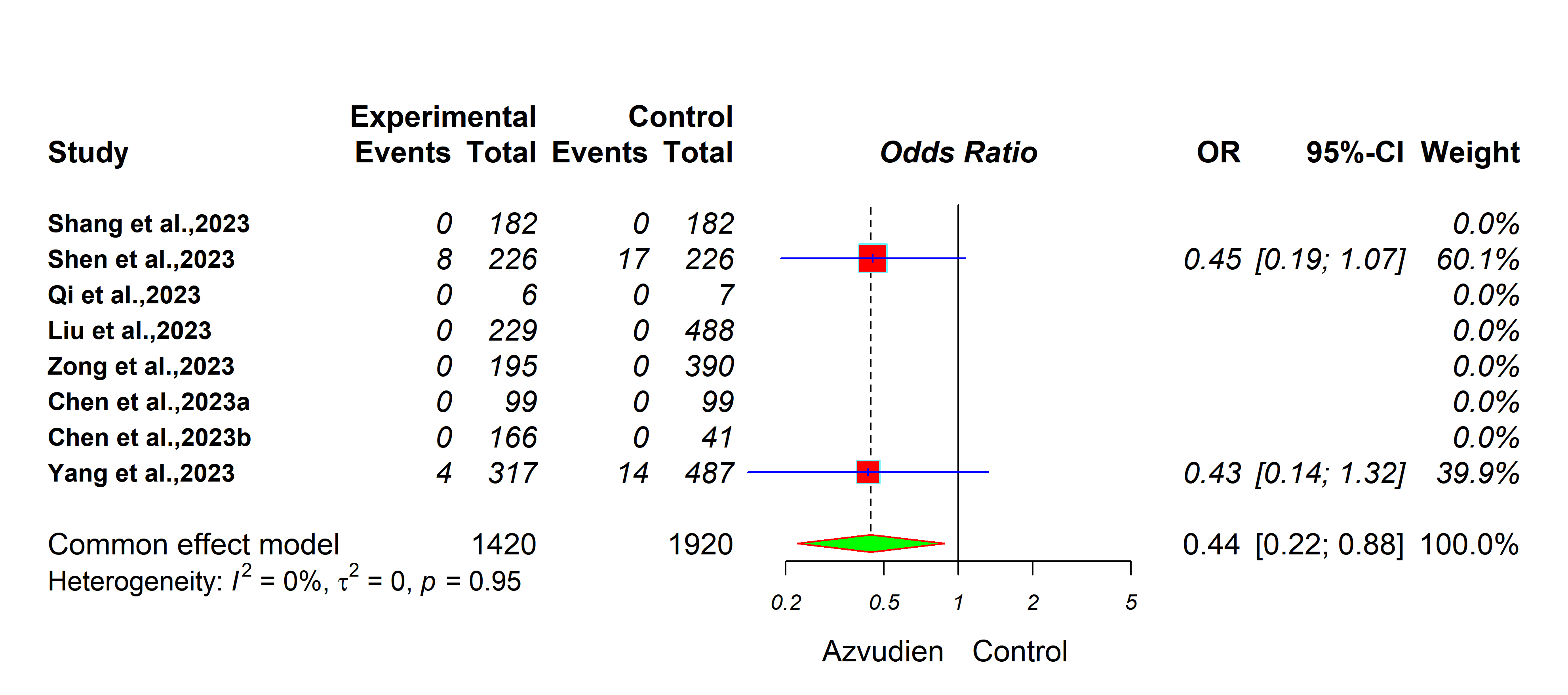


Figure S37. Composite disease progression (Removing Sun et al.,2023).


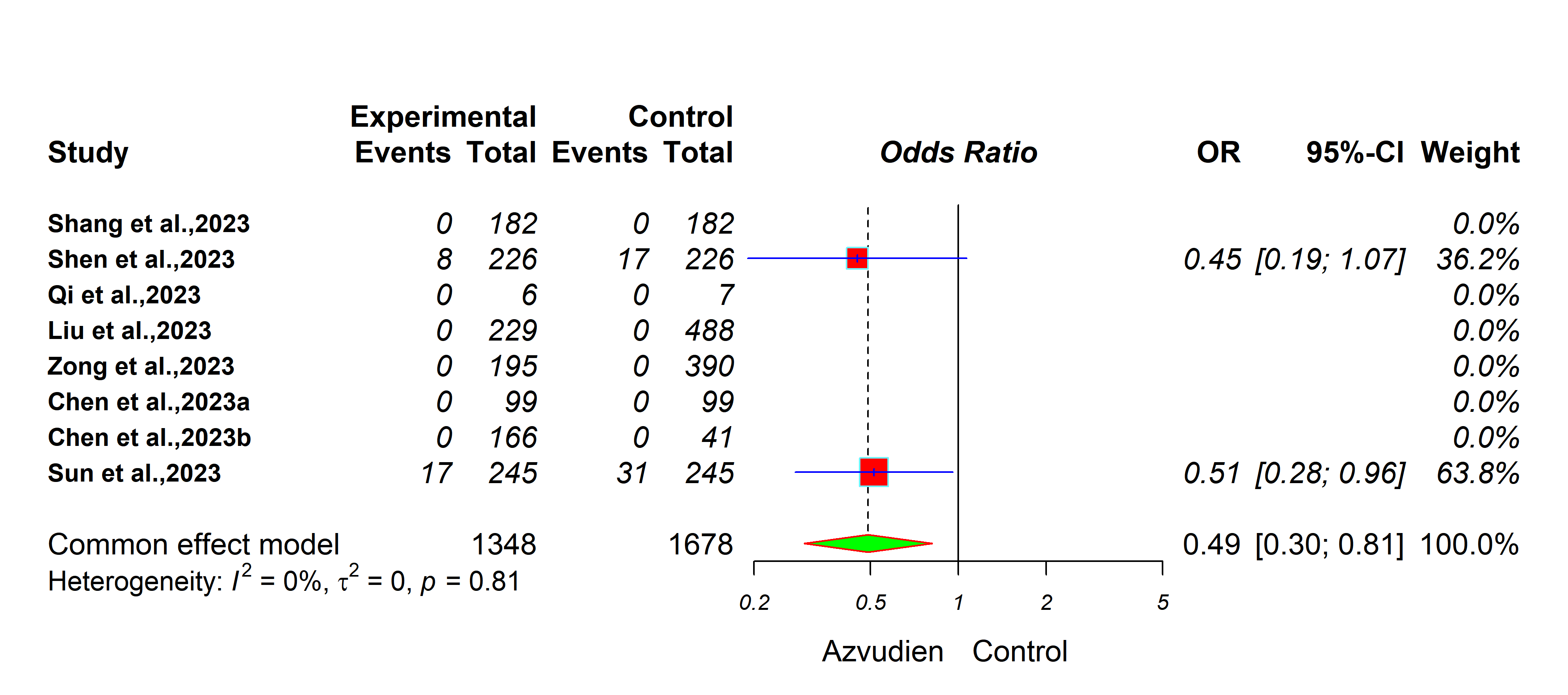


Figure S38. Composite disease progression (Removing Yang et al.,2023).

5. Adverse events in Retrospective cohort studies


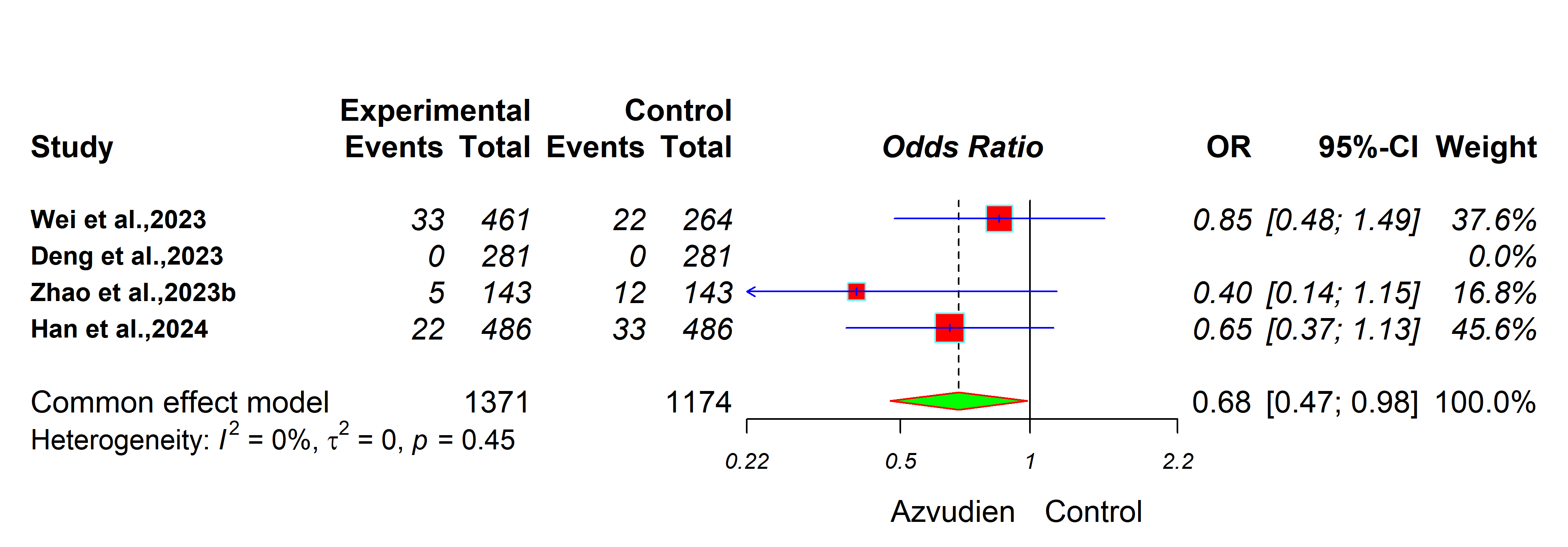


Figure S39. Adverse events (Removing Zhao et al.,2023a).


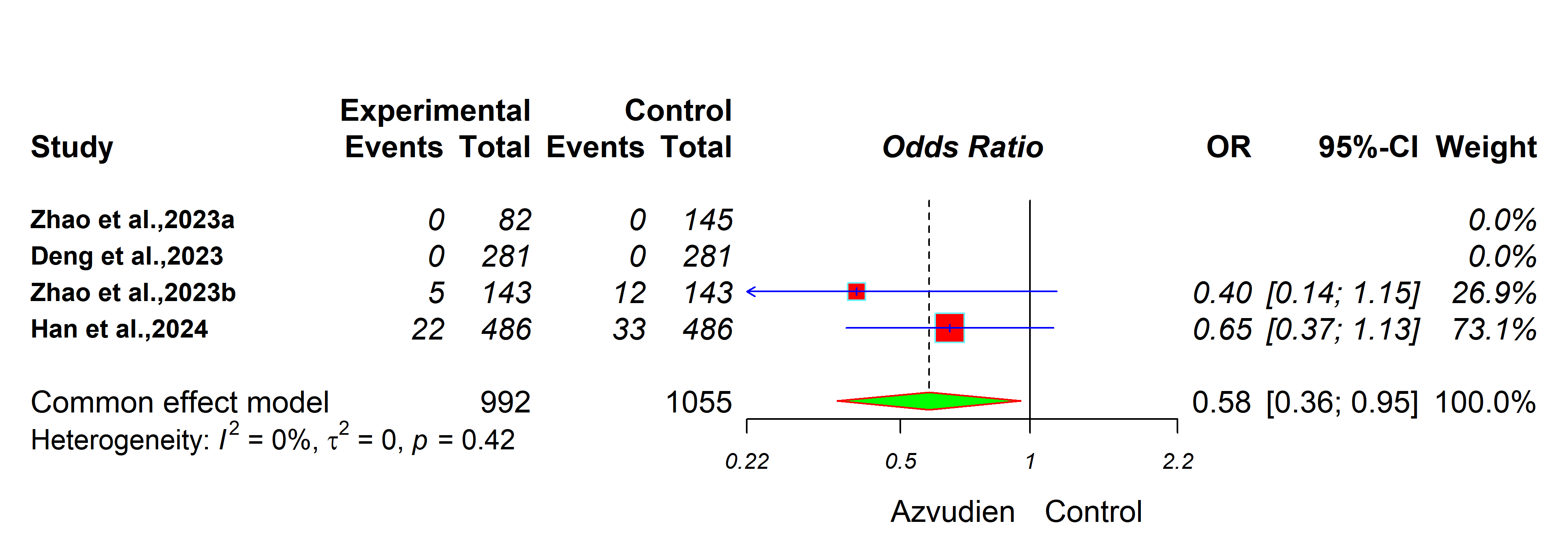


Figure S40. Adverse events (Removing Wei et al.,2023).


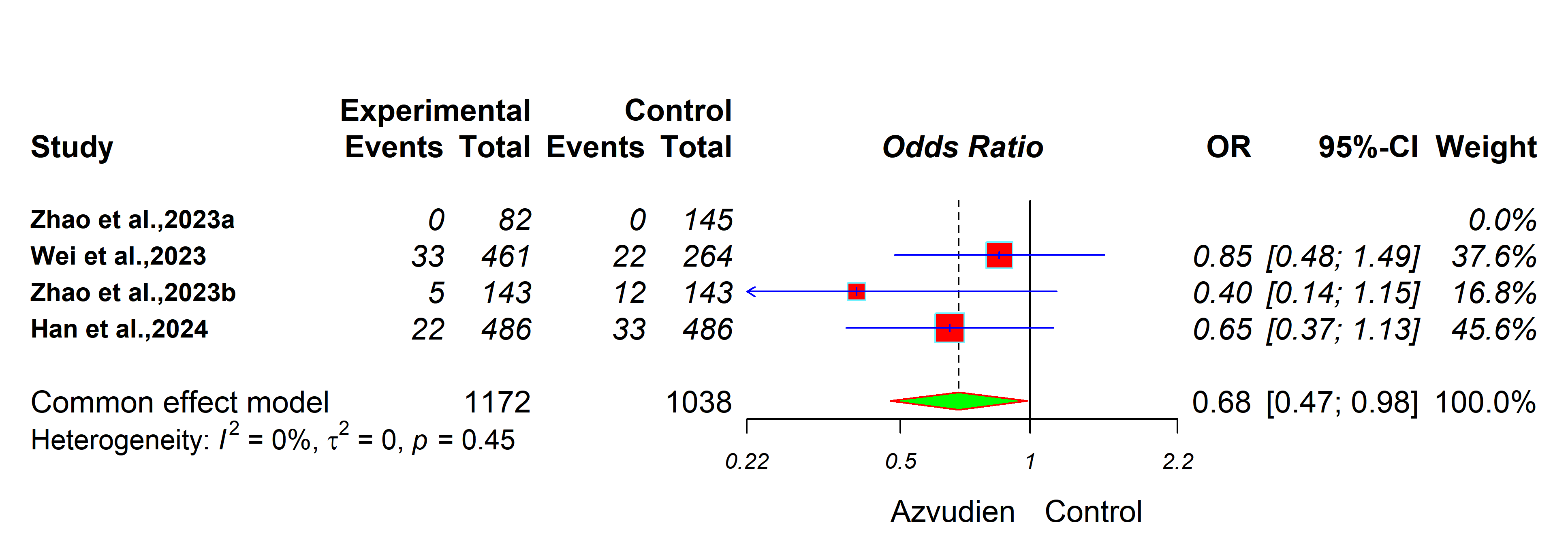


Figure S41. Adverse events (Removing Deng et al.,2023).


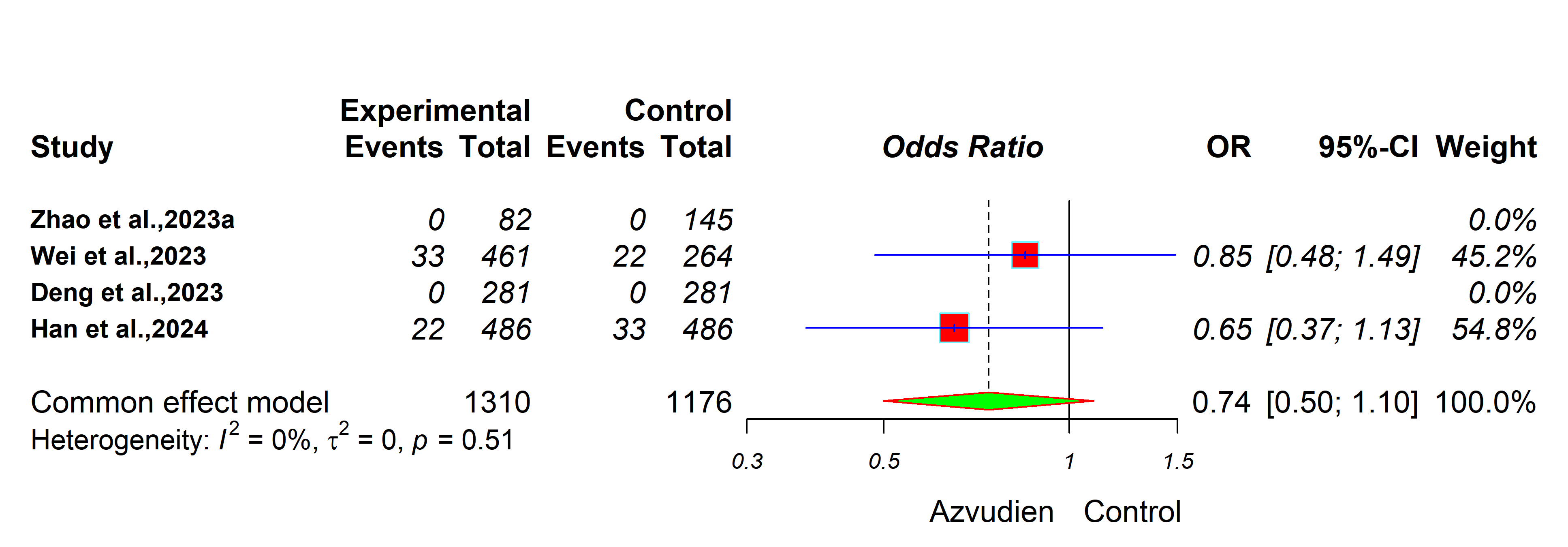


Figure S42. Adverse events (Removing Zhao et al.,2023b).


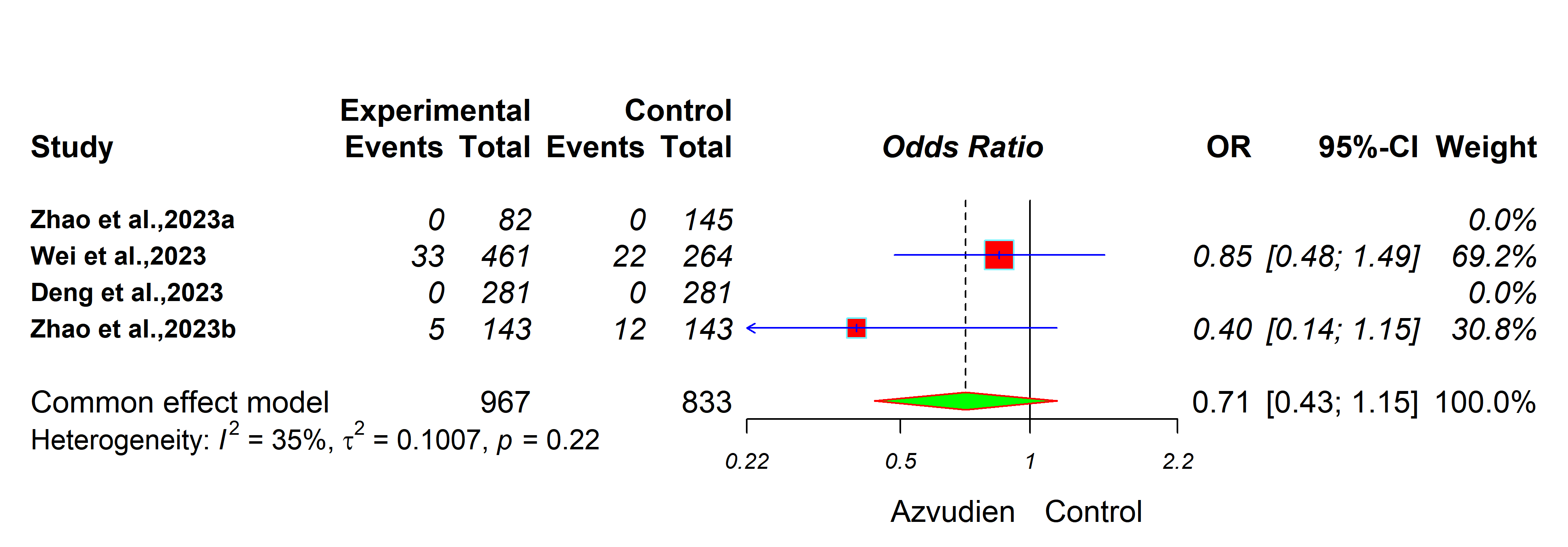


Figure S43. Adverse events (Removing Han et al.,2024).


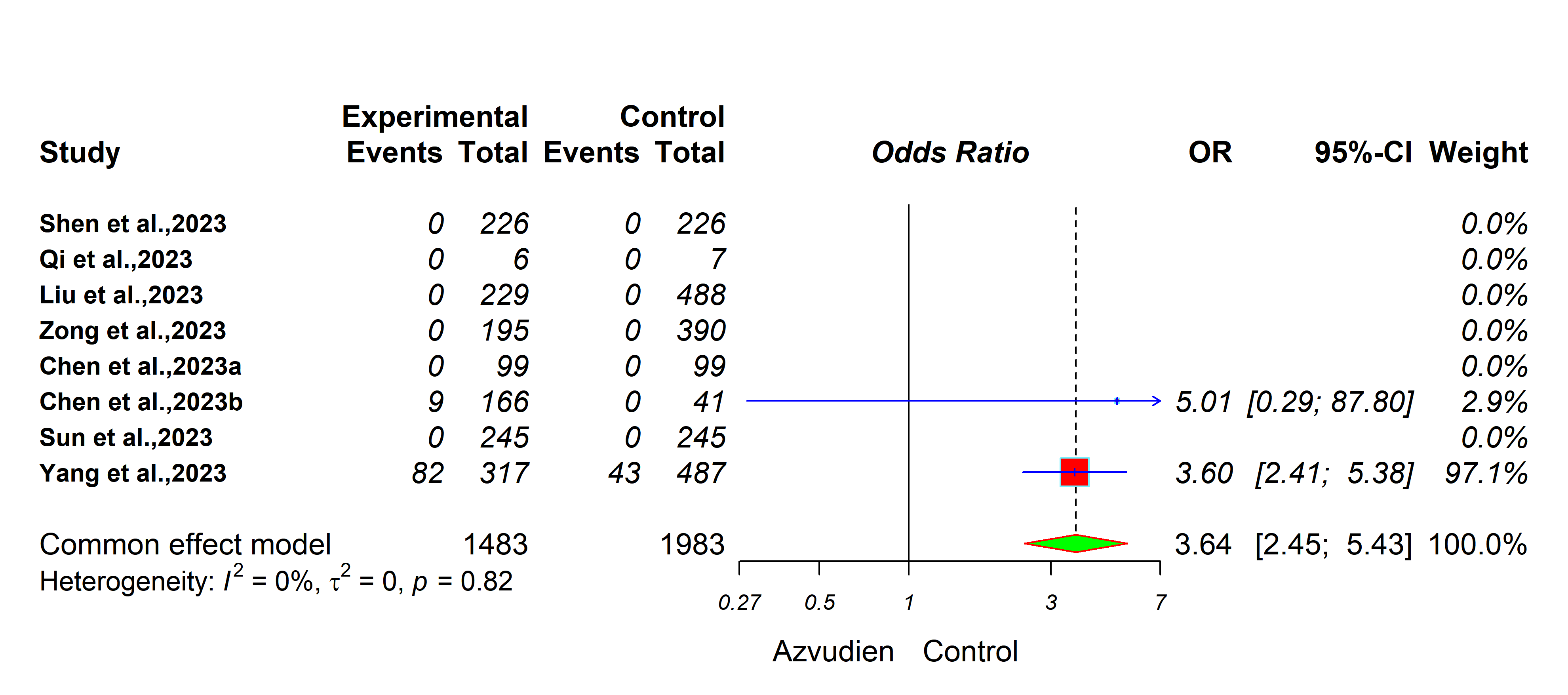


Figure S44. Adverse events (Removing Shang et al.,2023).


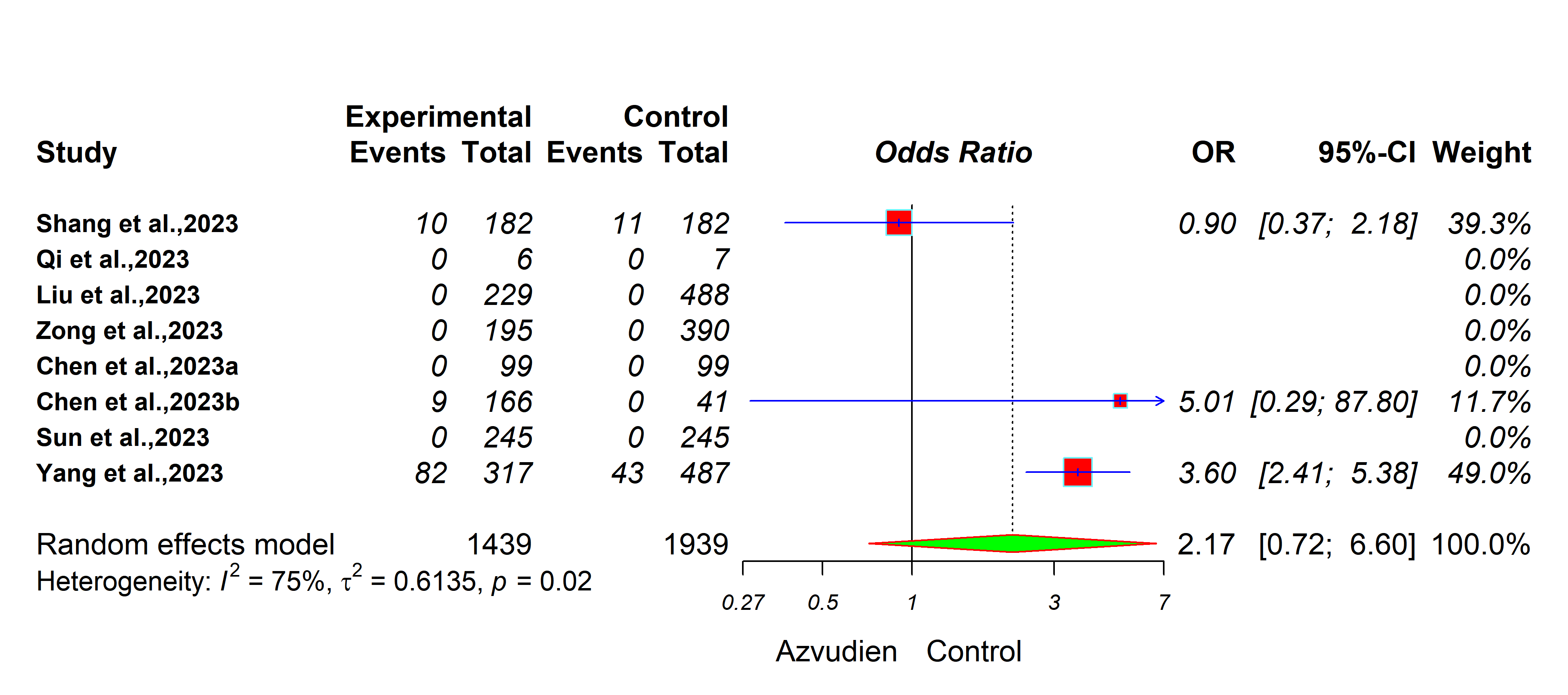


Figure S45. Adverse events (Removing Shen et al.,2023).


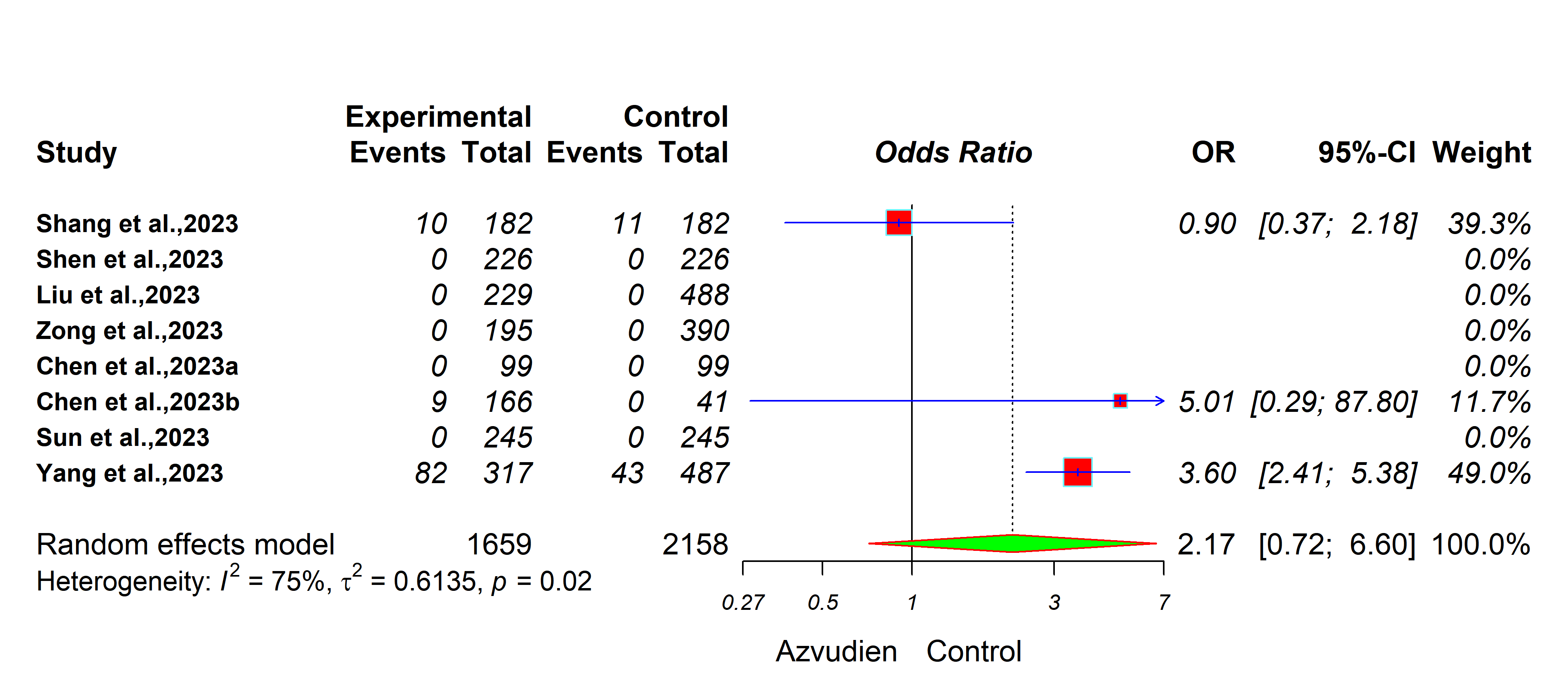


Figure S46. Adverse events (Removing Qi et al.,2023).


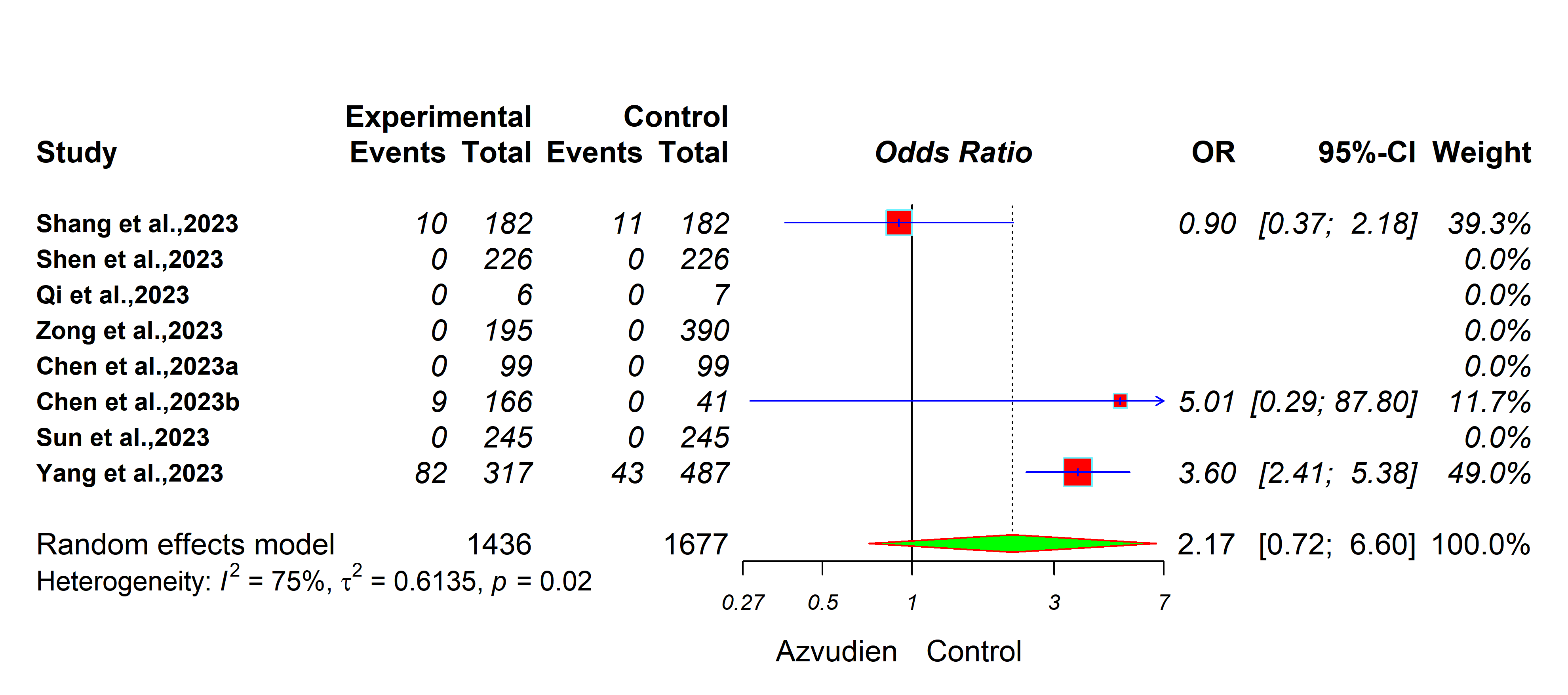


Figure S47. Adverse events (Removing Liu et al.,2023).


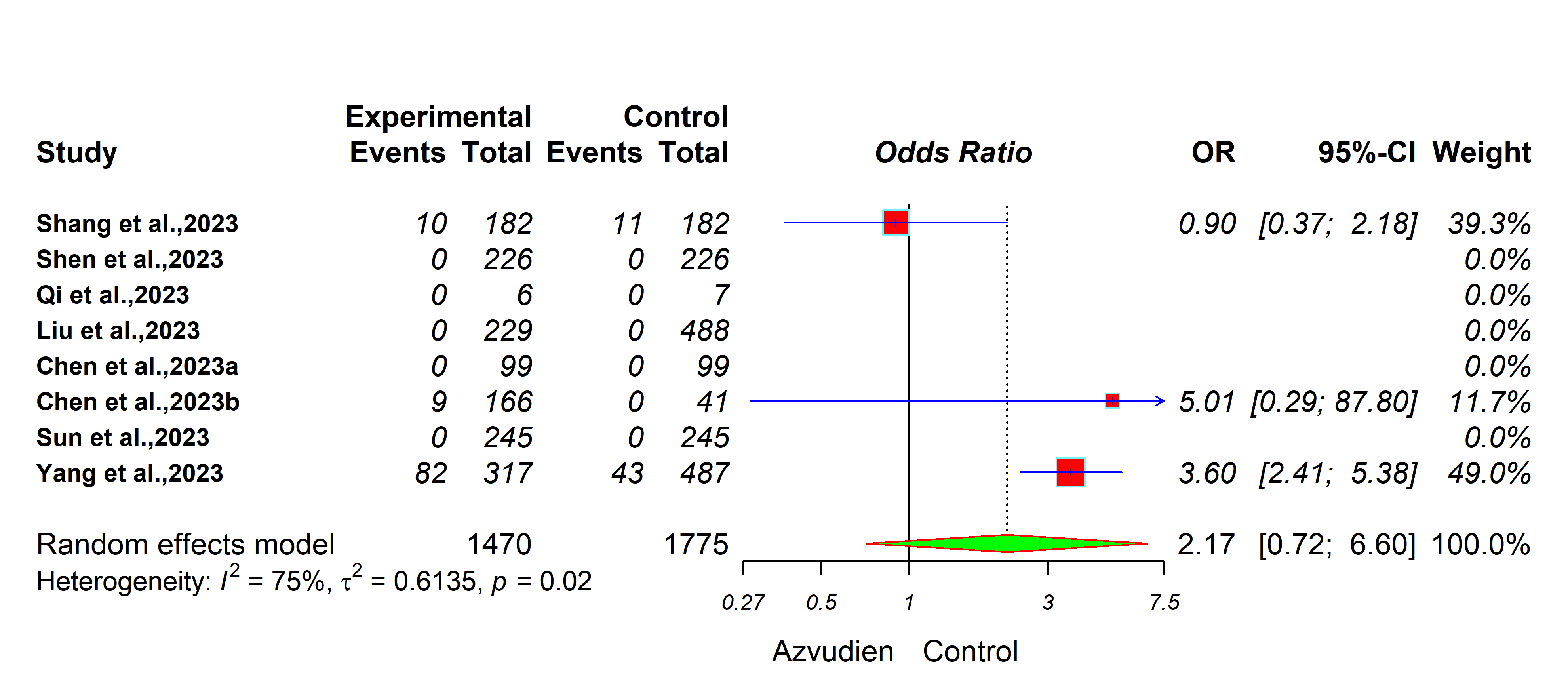


Figure S48. Adverse events (Removing Zong et al.,2023).


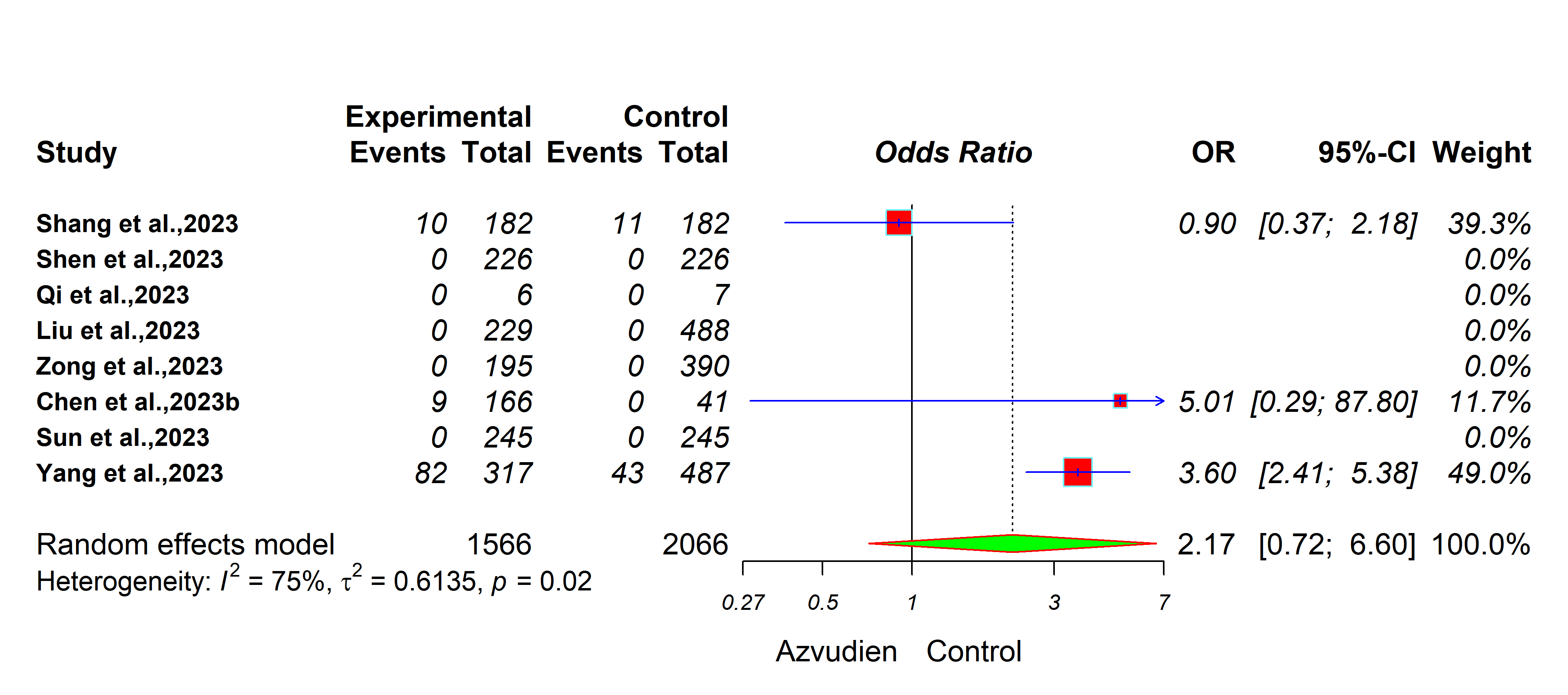


Figure S49. Adverse events (Removing Chen et al.,2023a).


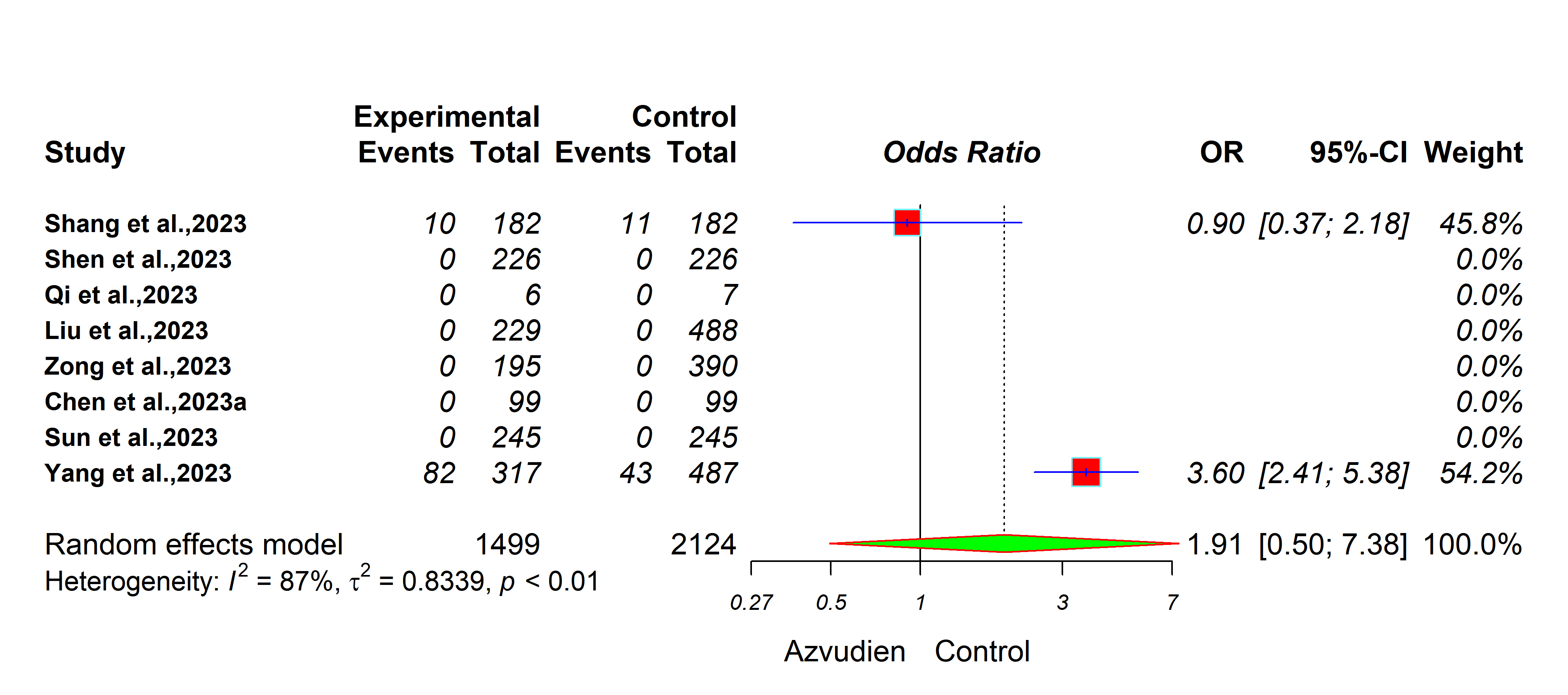


Figure S50. Adverse events (Removing Chen et al.,2023b).


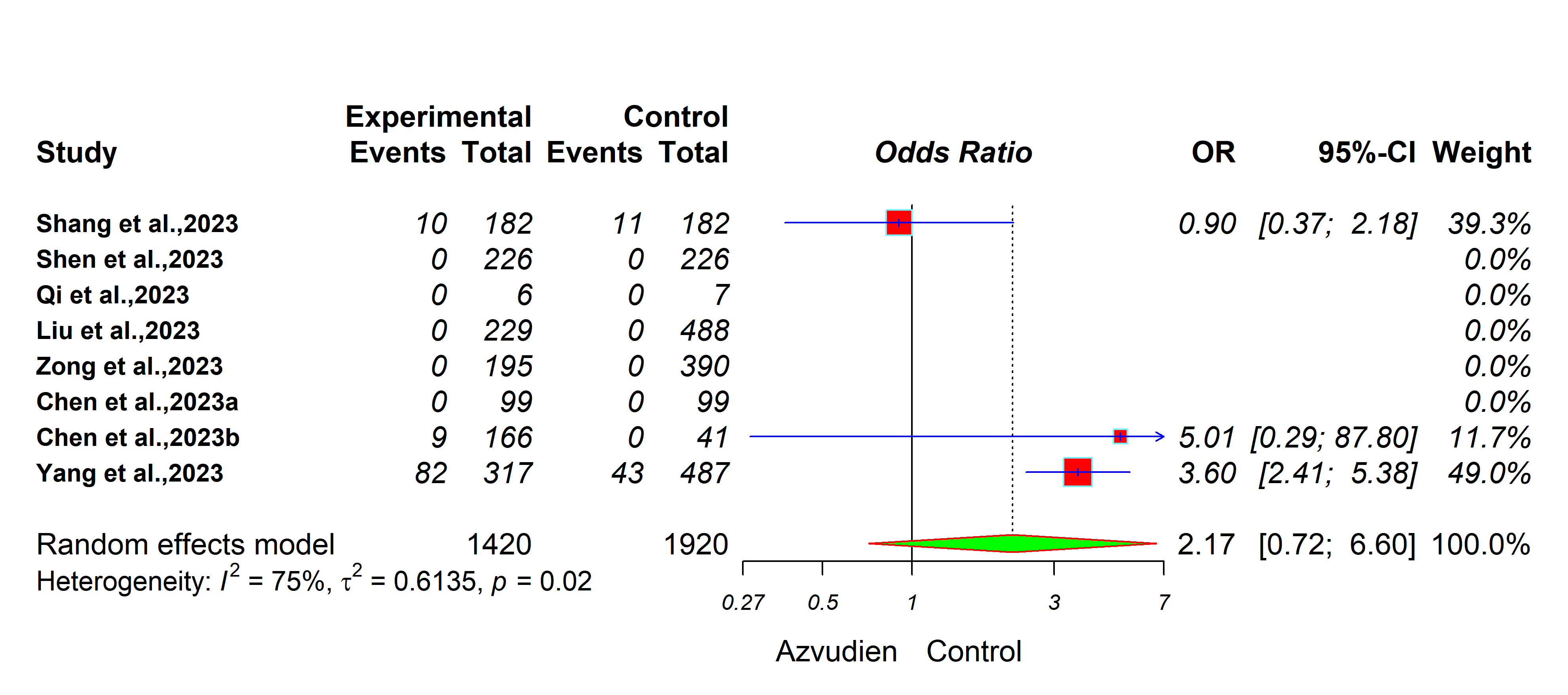


Figure S51. Adverse events (Removing Sun et al.,2023).


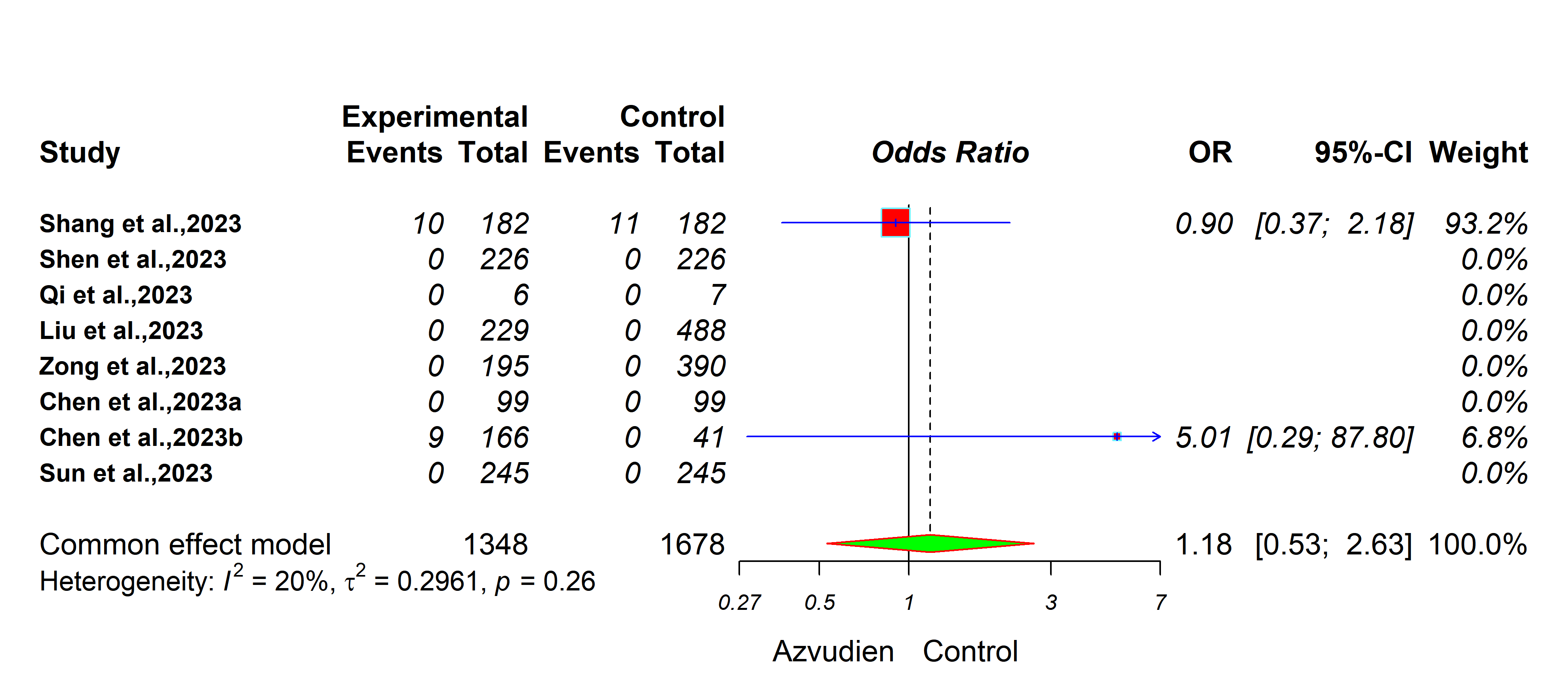


Figure S52. Adverse events (Removing Yang et al.,2023).

6. Adverse events in all included studies


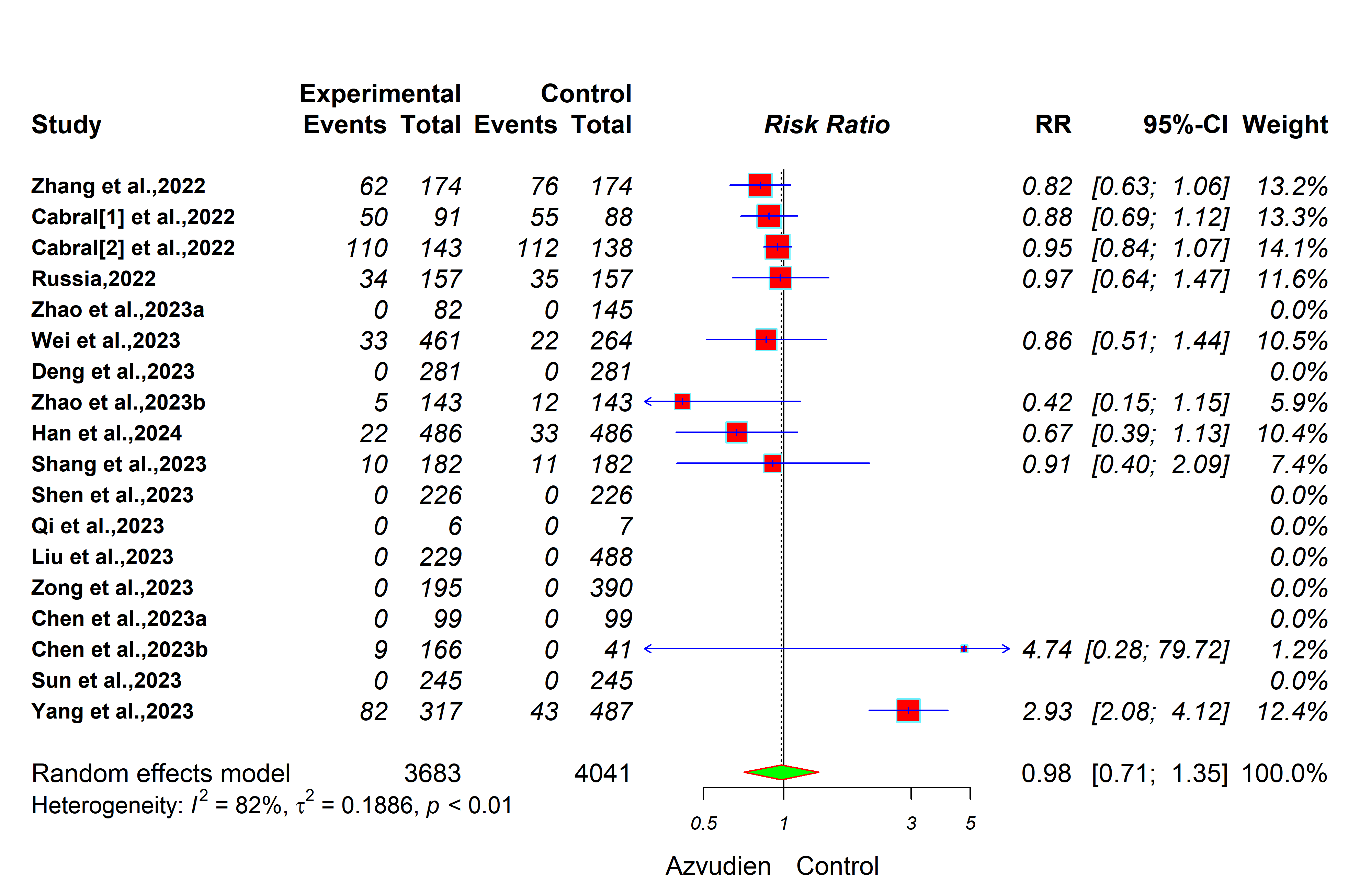


Figure S 53. Adverse events (Removing Ren et al.,2020).


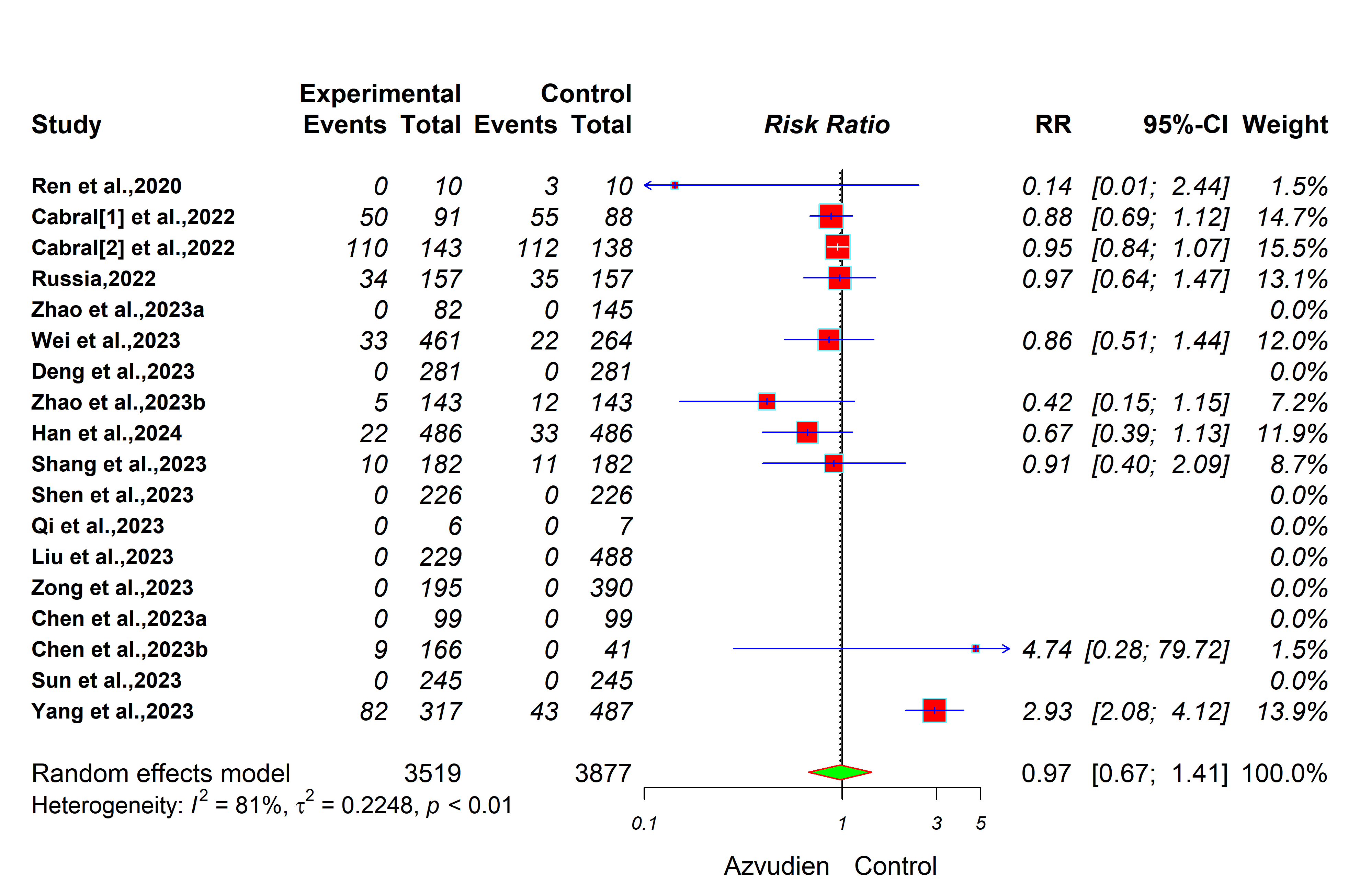


Figure S 54. Adverse events (Removing Zhang et al.,2022).


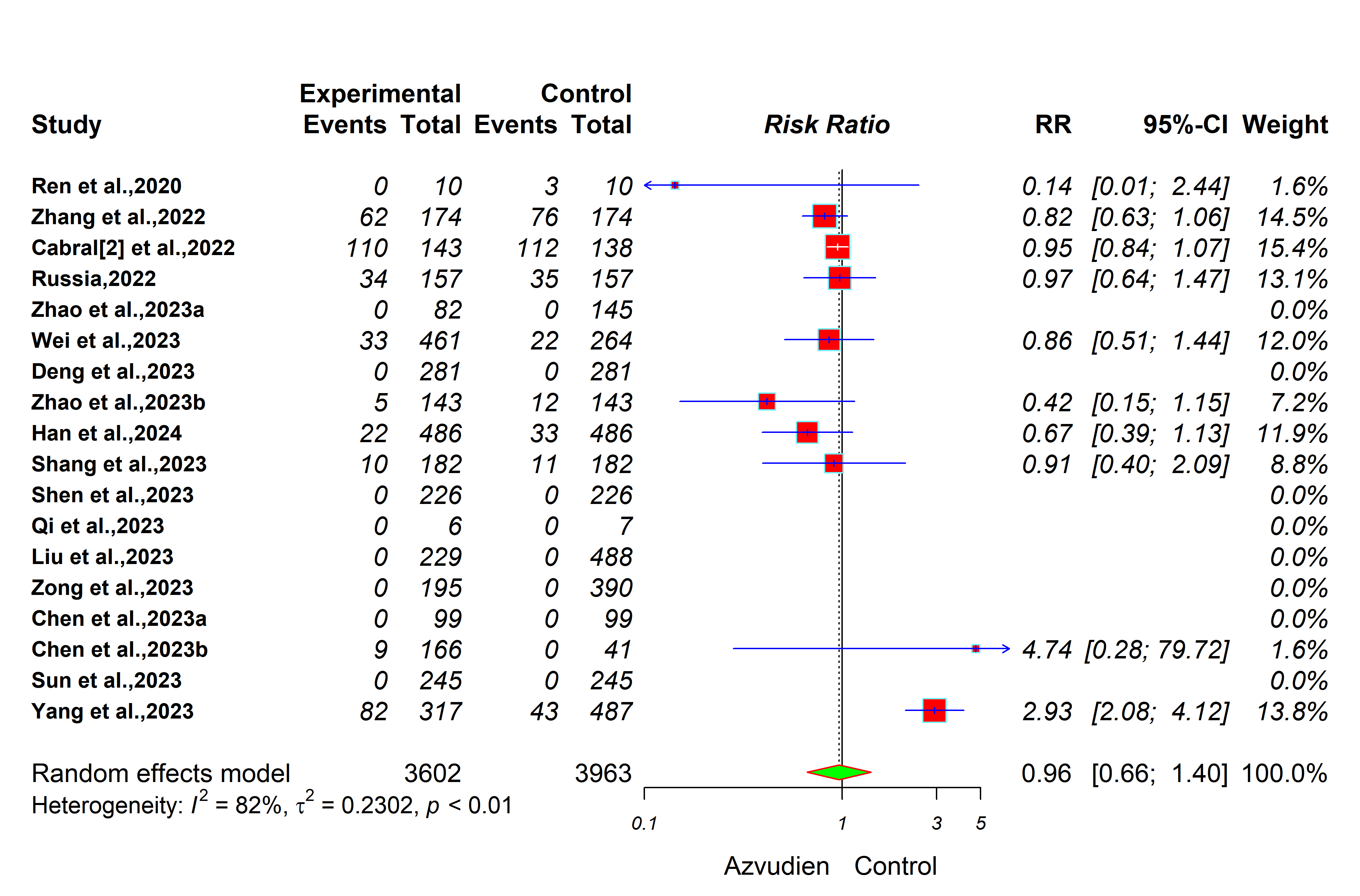


Figure S 55. Adverse events (Removing Cabral[1] et al.,2022).


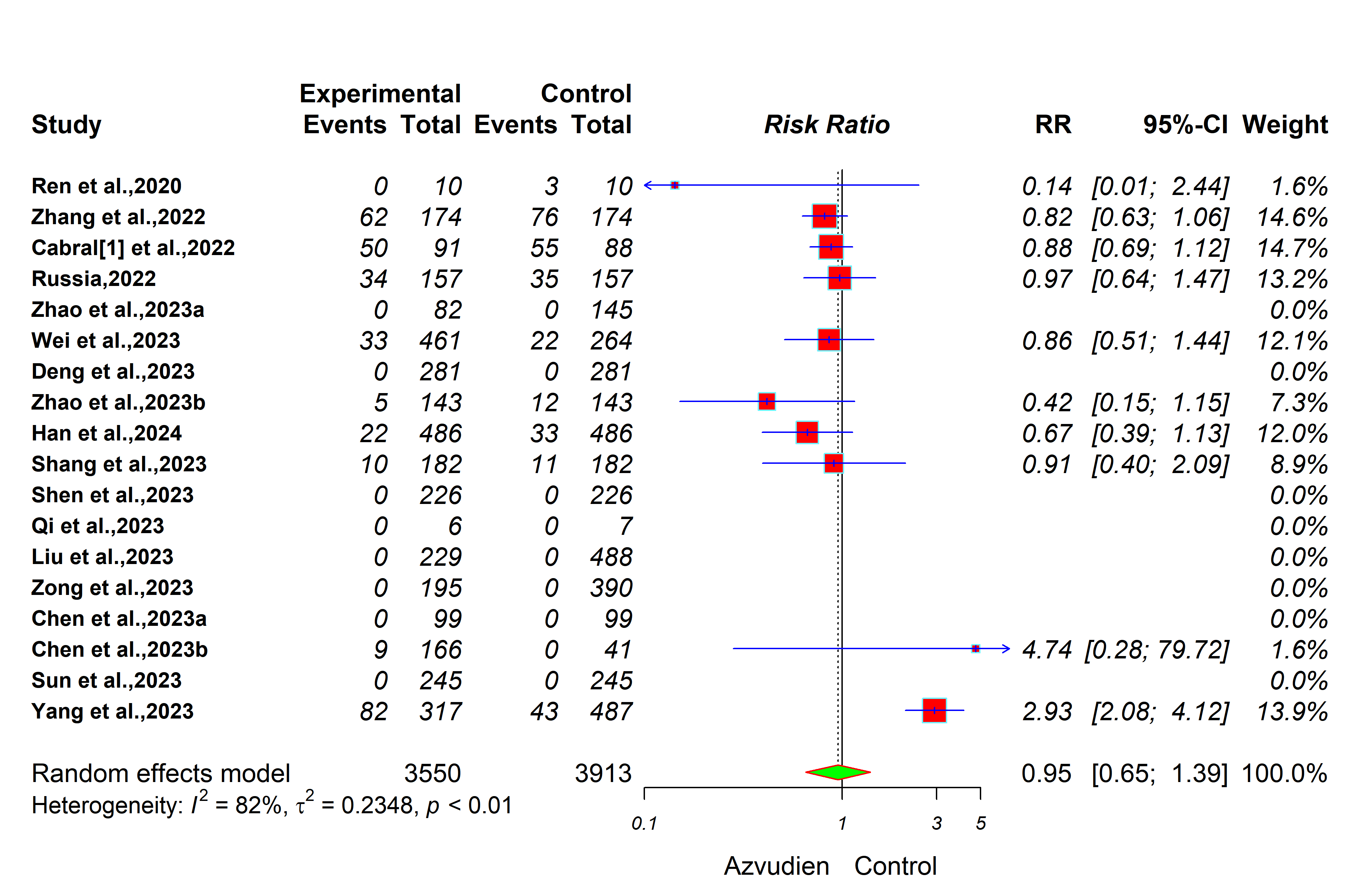


Figure S 56. Adverse events (Removing Cabral[2] et al.,2022).


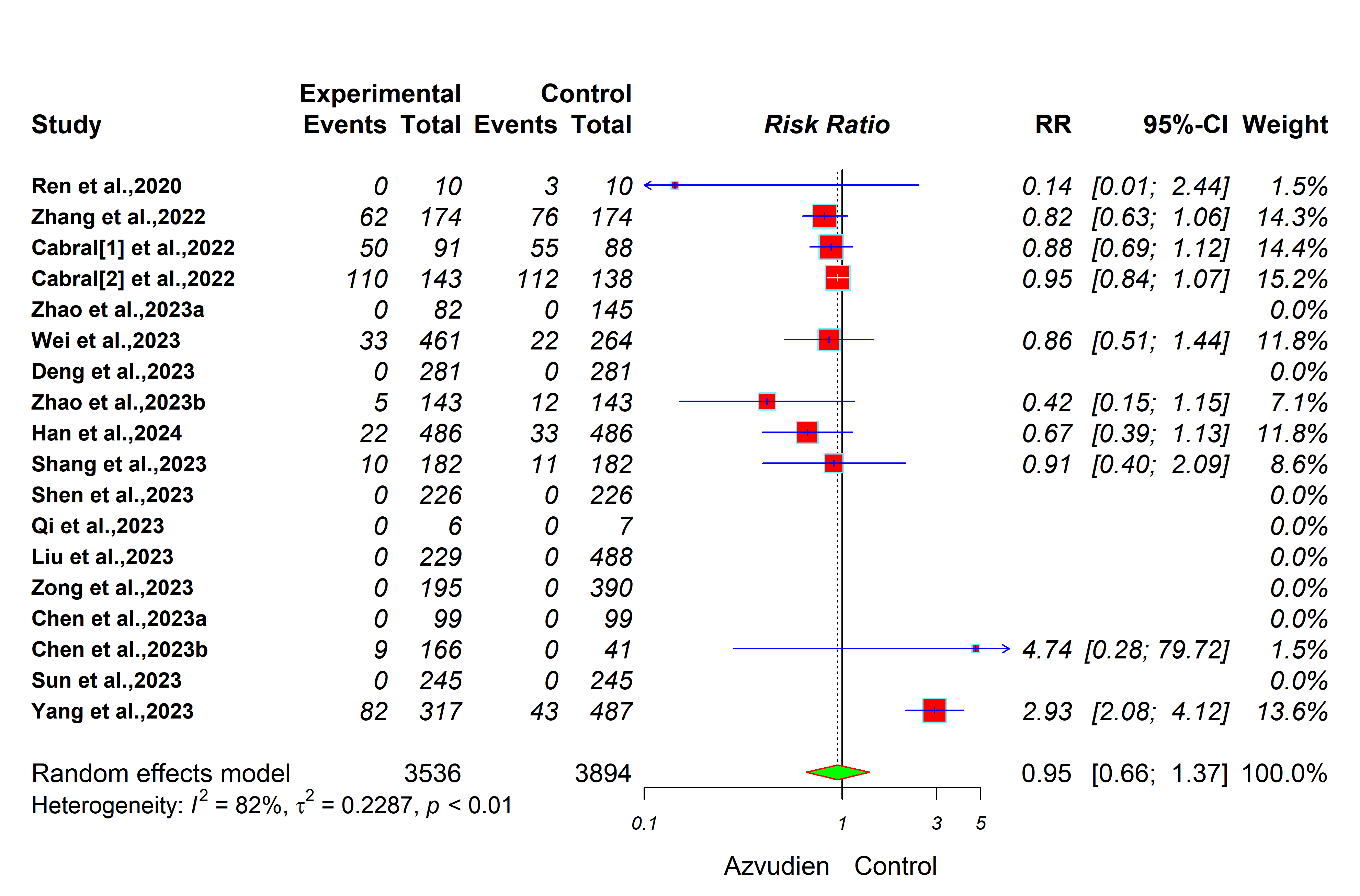


Figure S 57. Adverse events (Removing Russia, 2022).


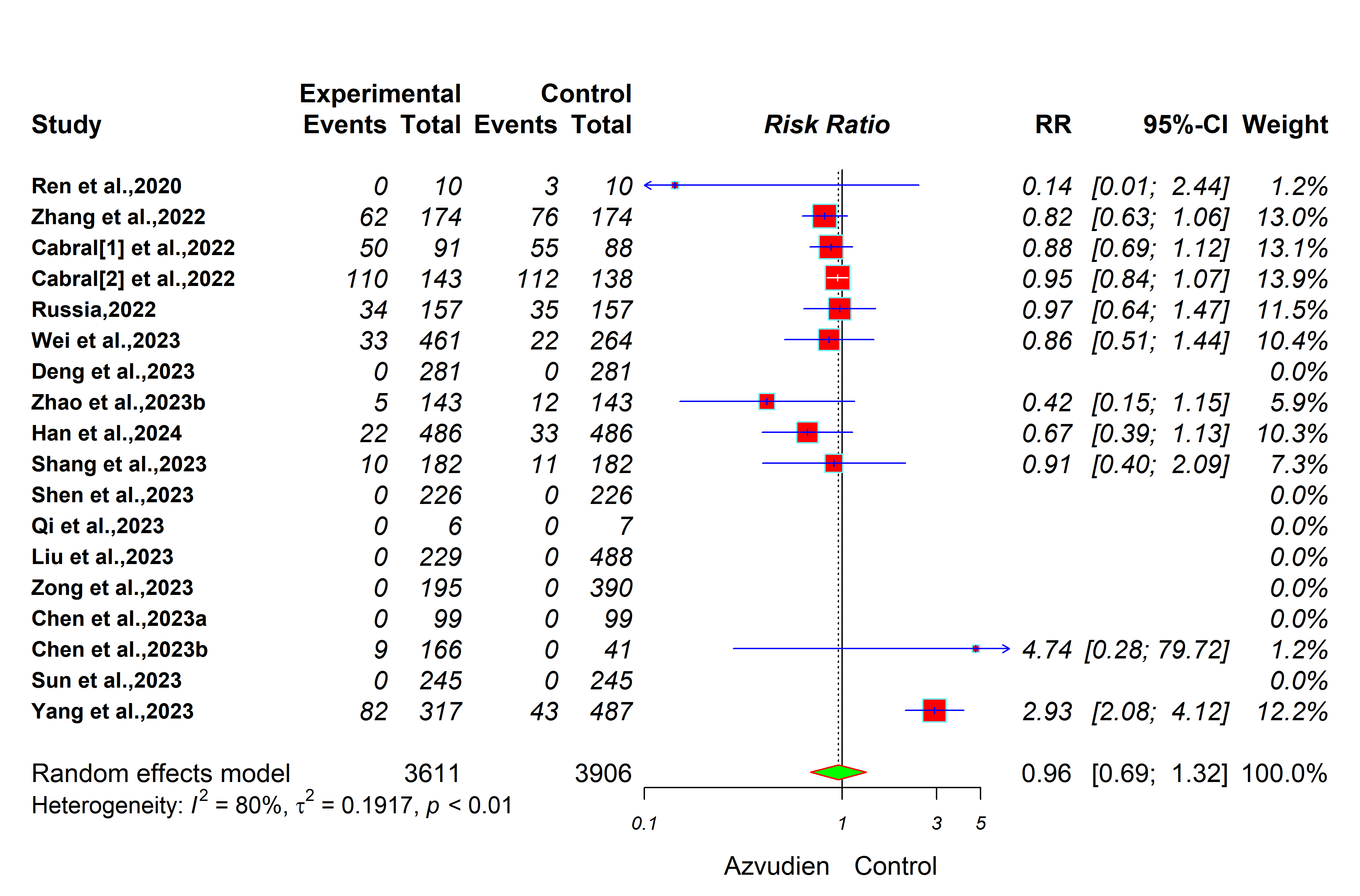


Figure S 58. Adverse events (Removing Zhao et al.,2023a).


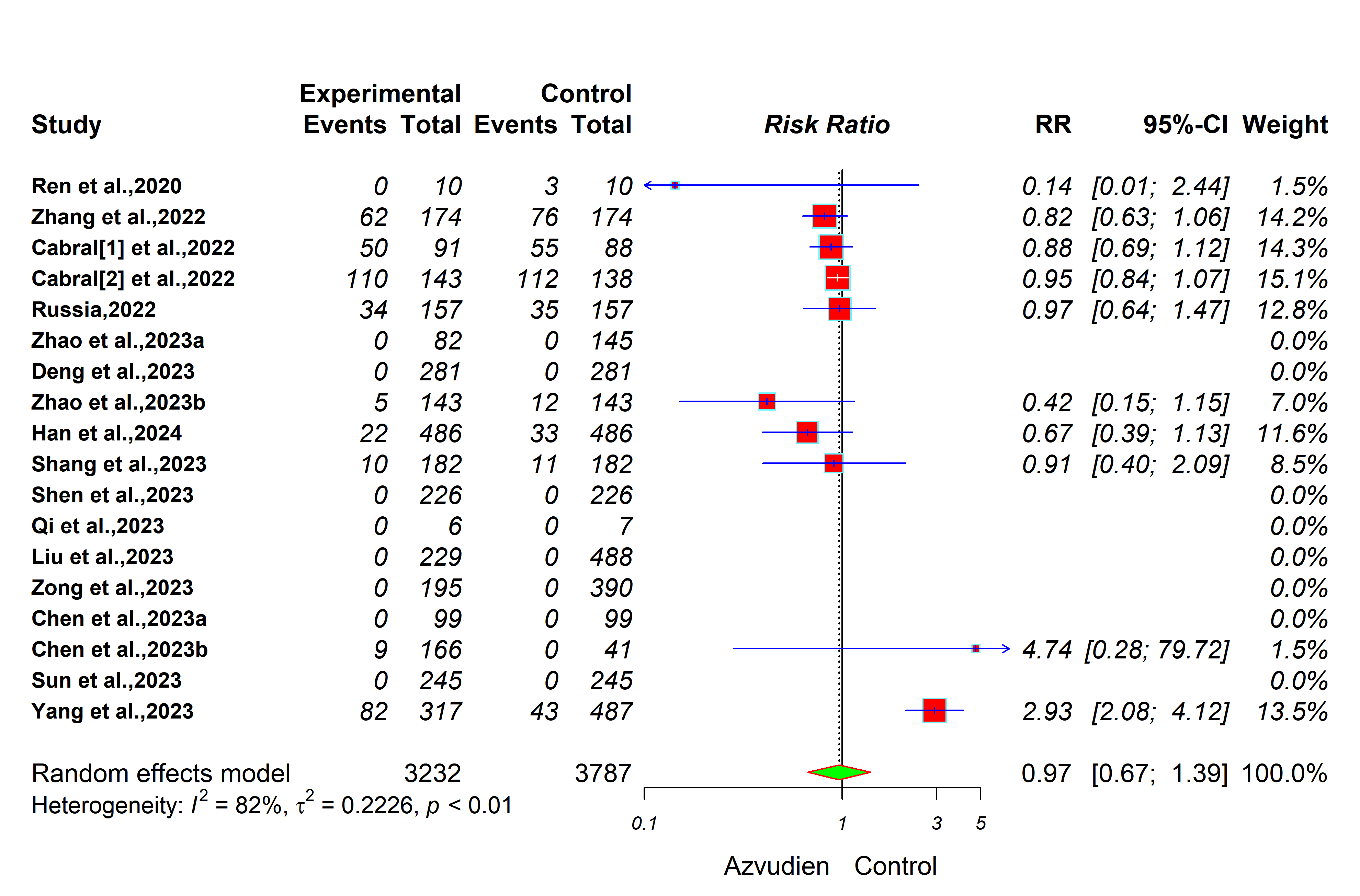


Figure S 59. Adverse events (Removing Wei et al.,2023).


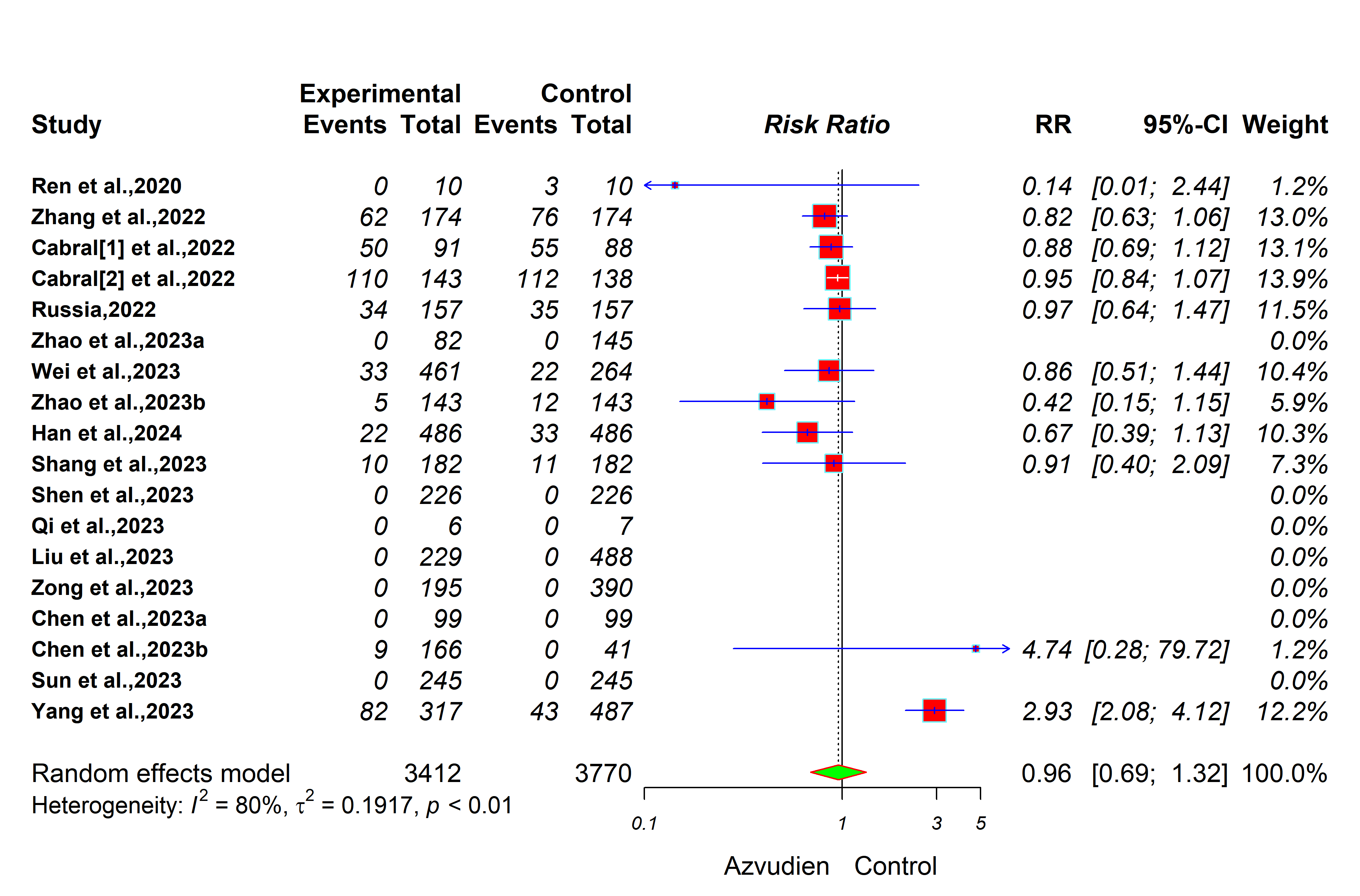


Figure S 60. Adverse events (Removing Deng et al.,2023).


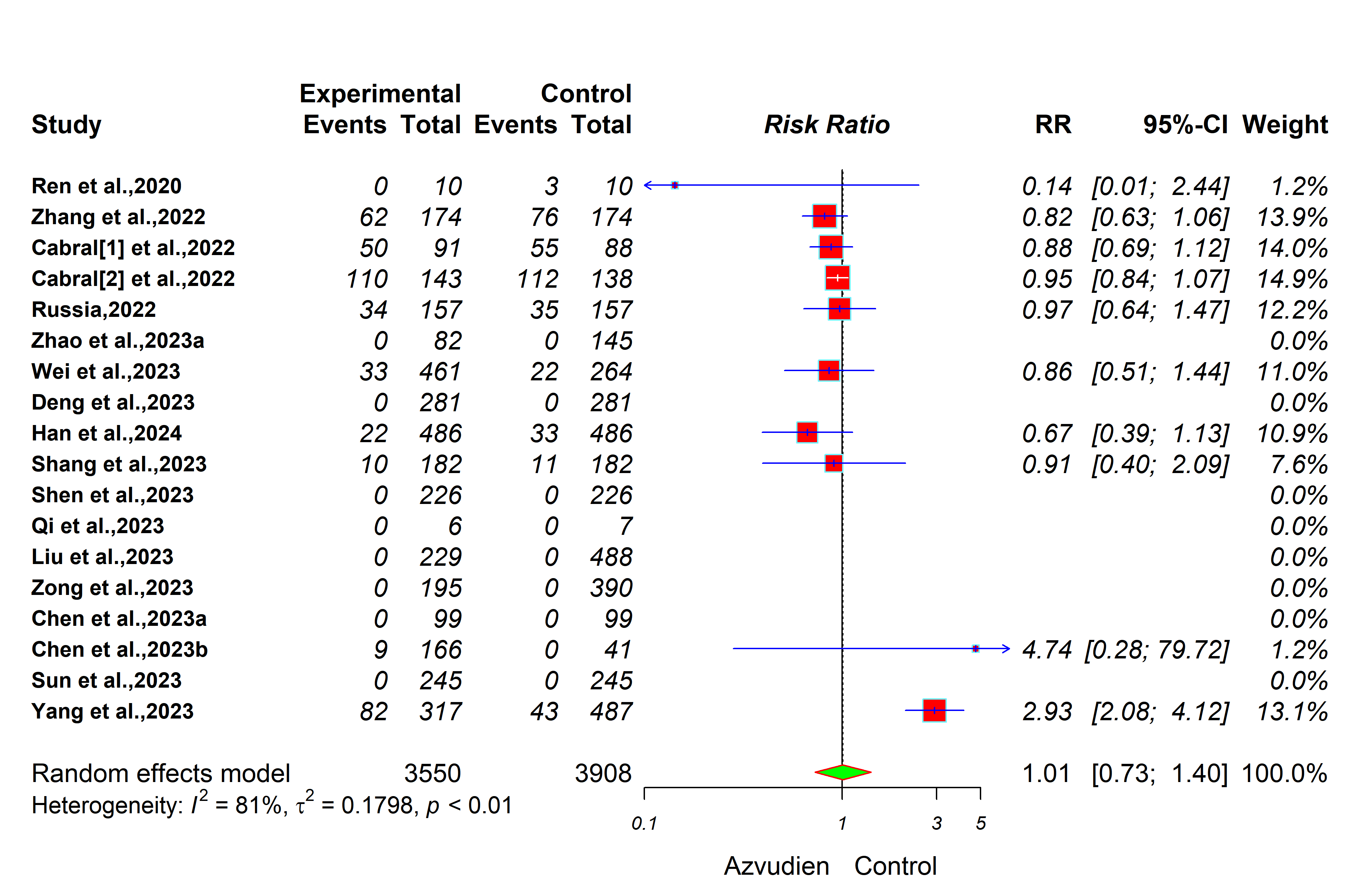


Figure S 61. Adverse events (Removing Zhao et al.,2023b).


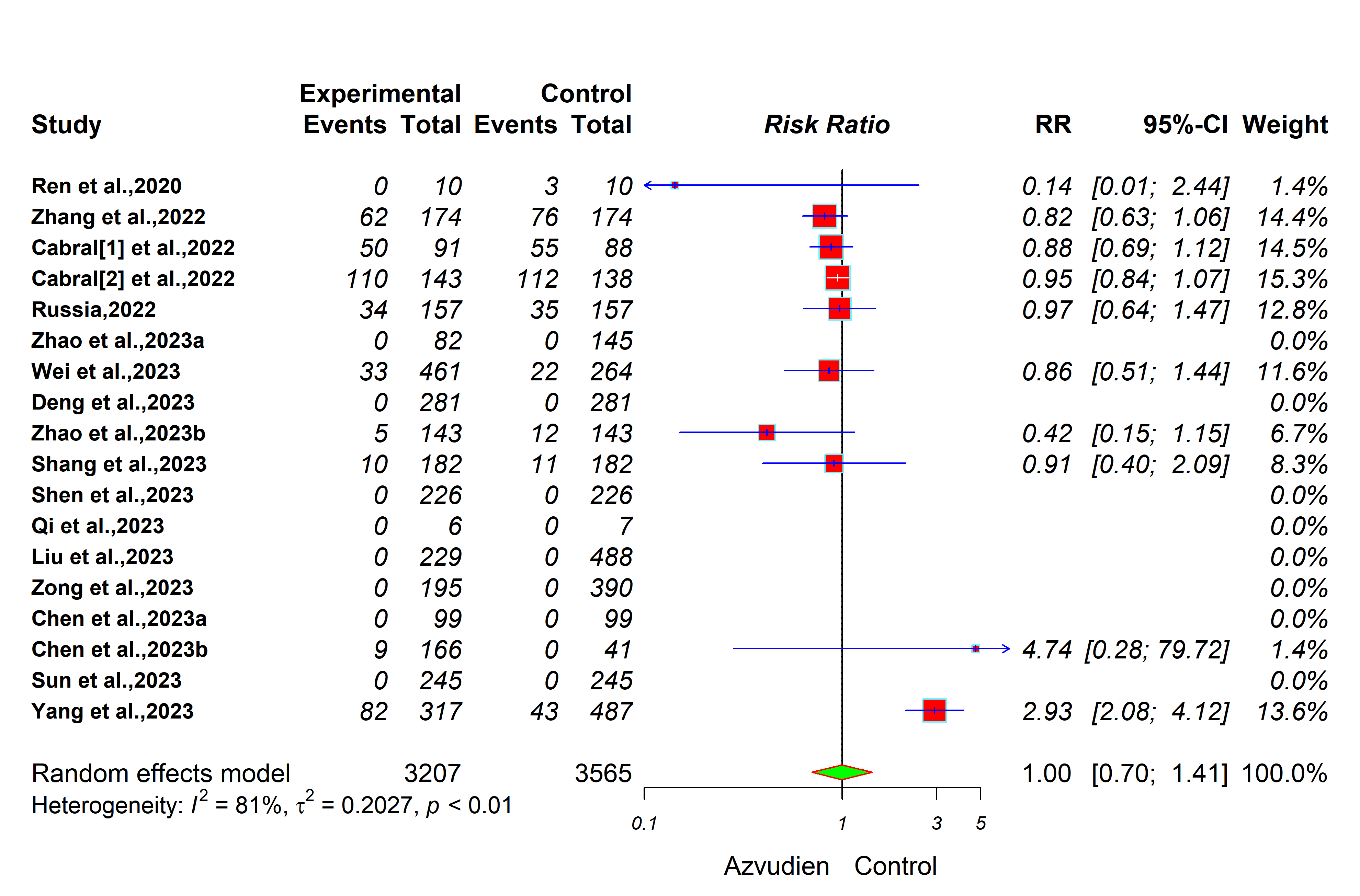


Figure S 62. Adverse events (Removing Han et al.,2024).


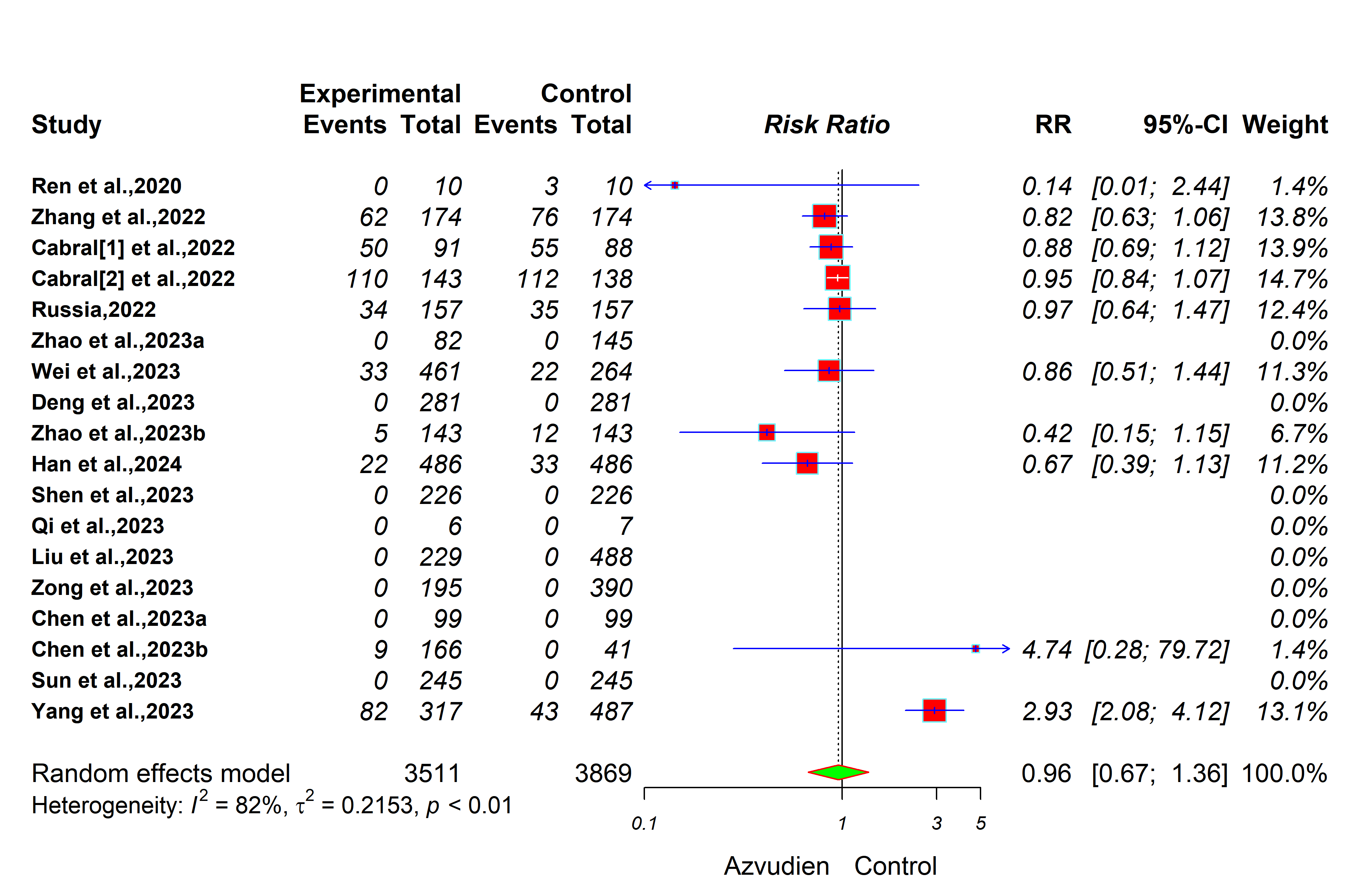


Figure S 63. All cause mortality (Removing Shang et al.,2023).


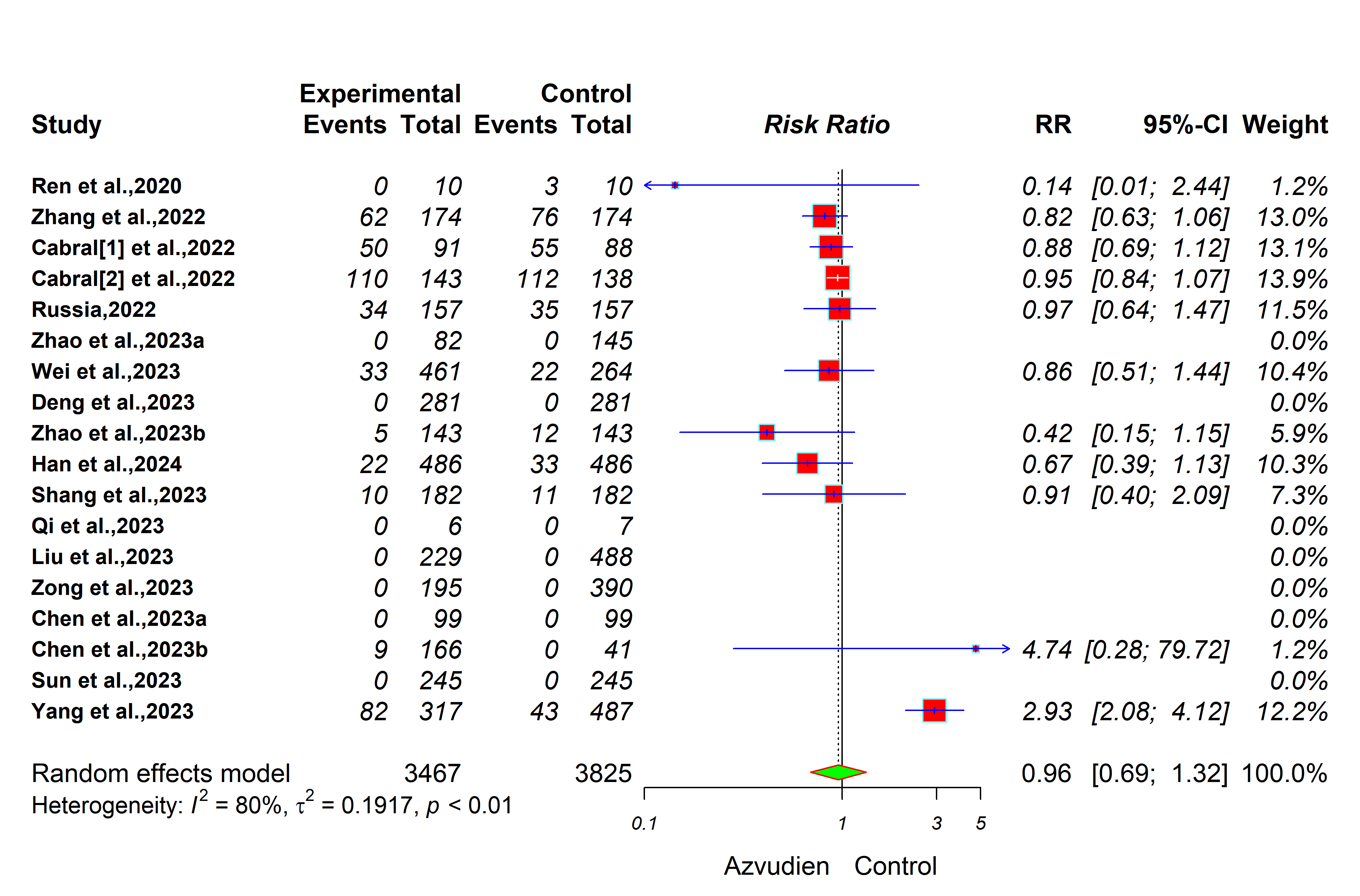


Figure S 64. Adverse events (Removing Shen et al.,2023).


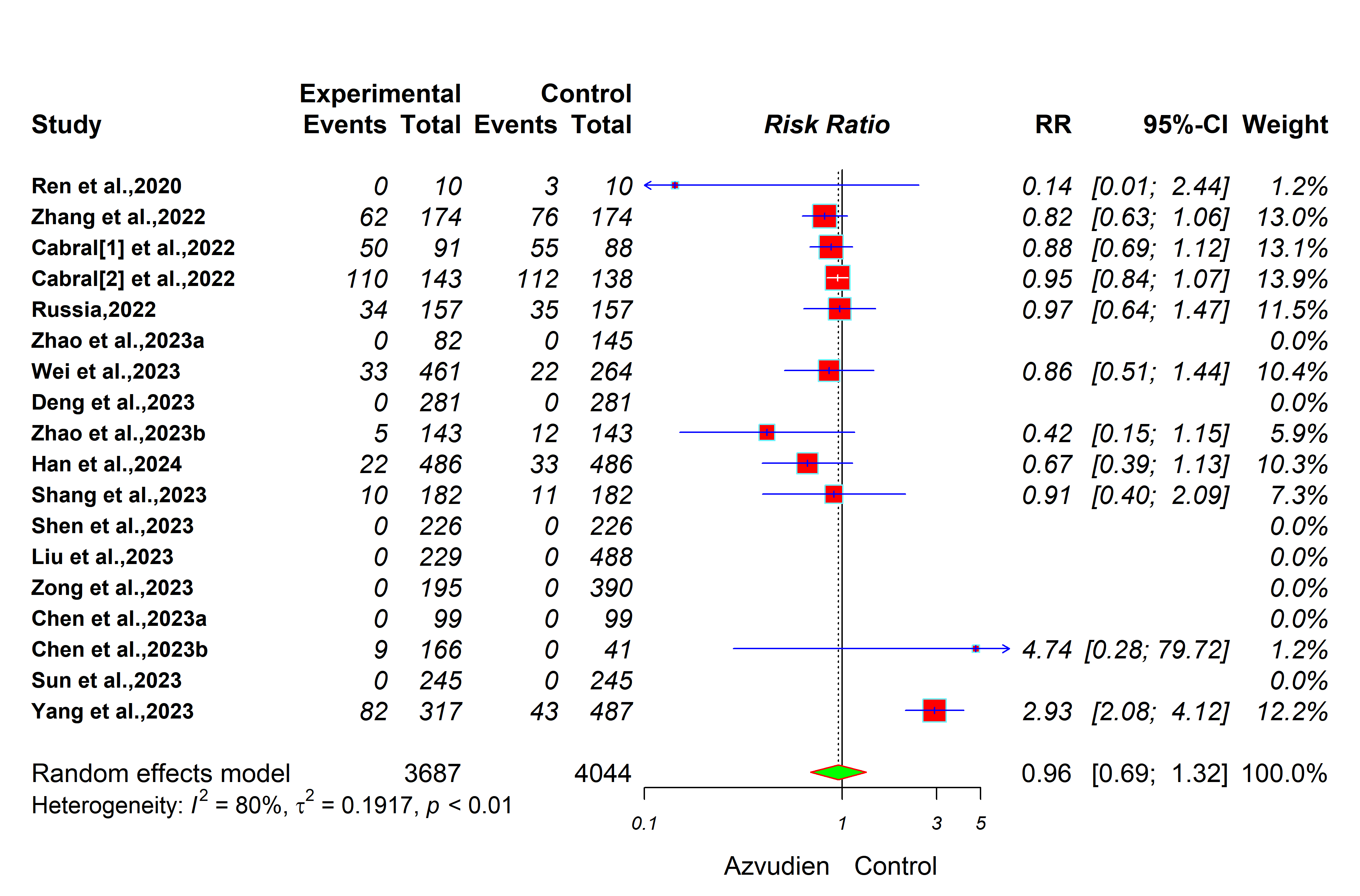


Figure S 65. Adverse events (Removing Qi et al.,2023).


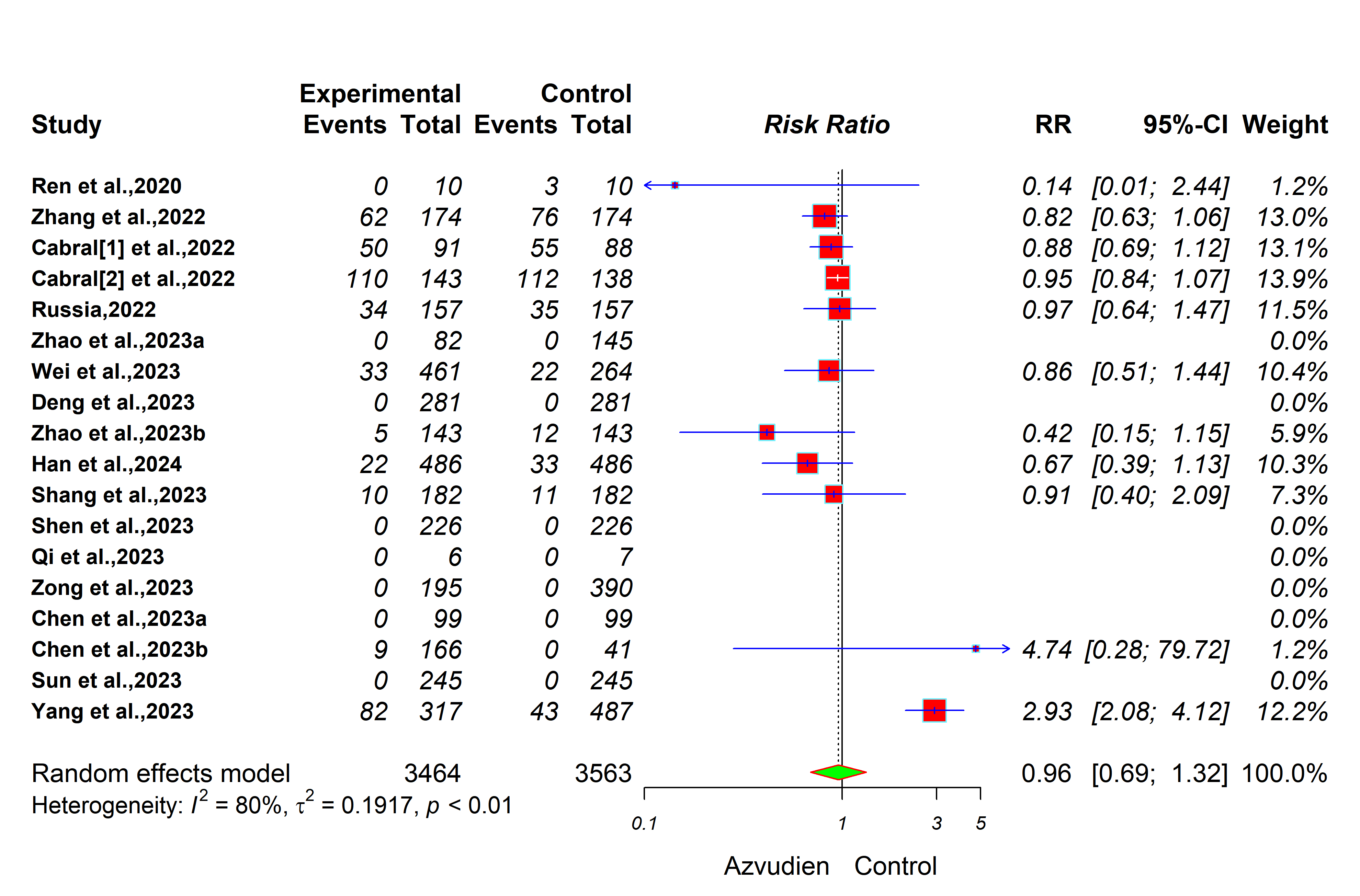


Figure S 66. Adverse events (Removing Liu et al.,2023).


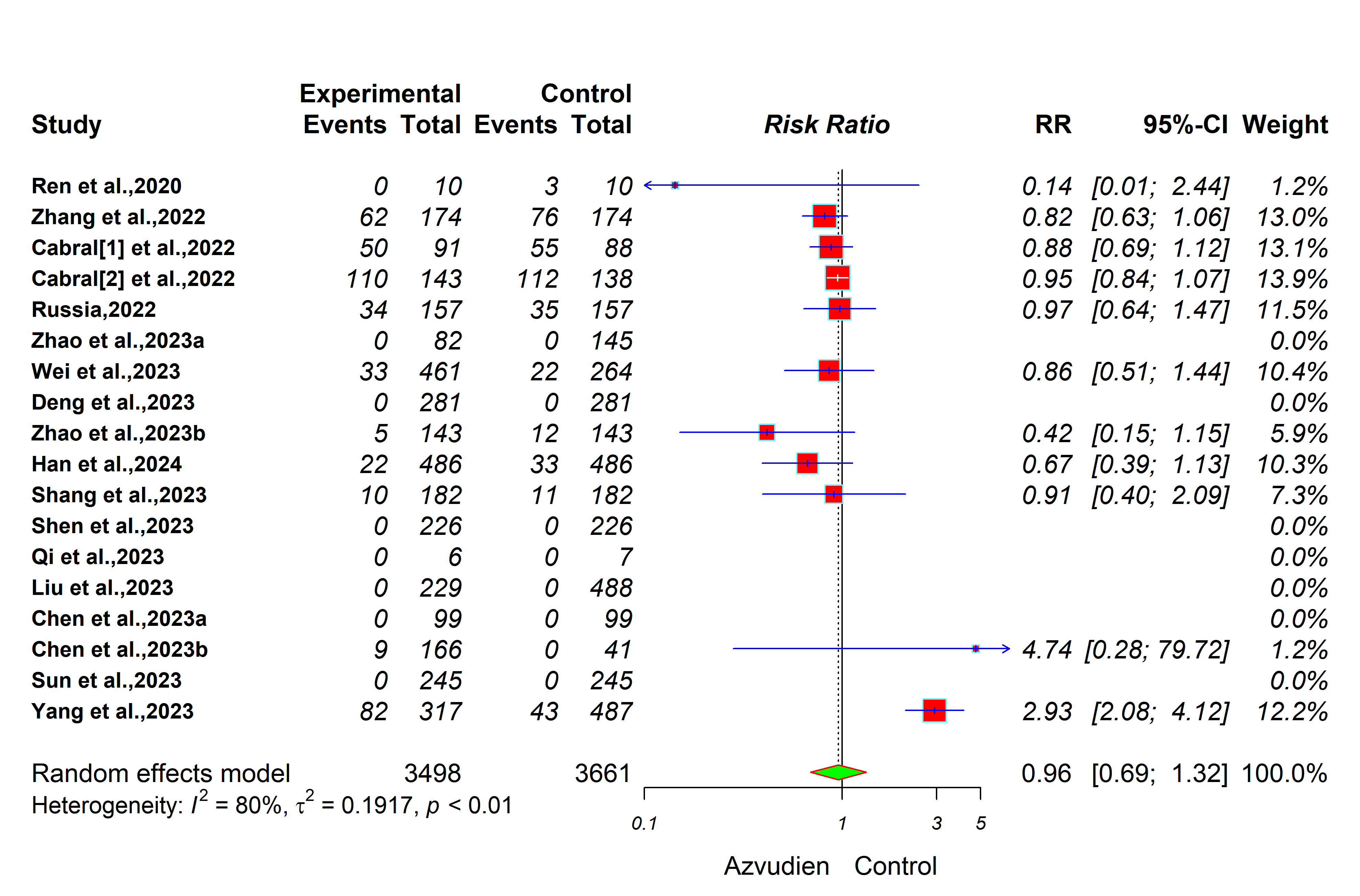


Figure S 67. Adverse events (Removing Zong et al.,2023).


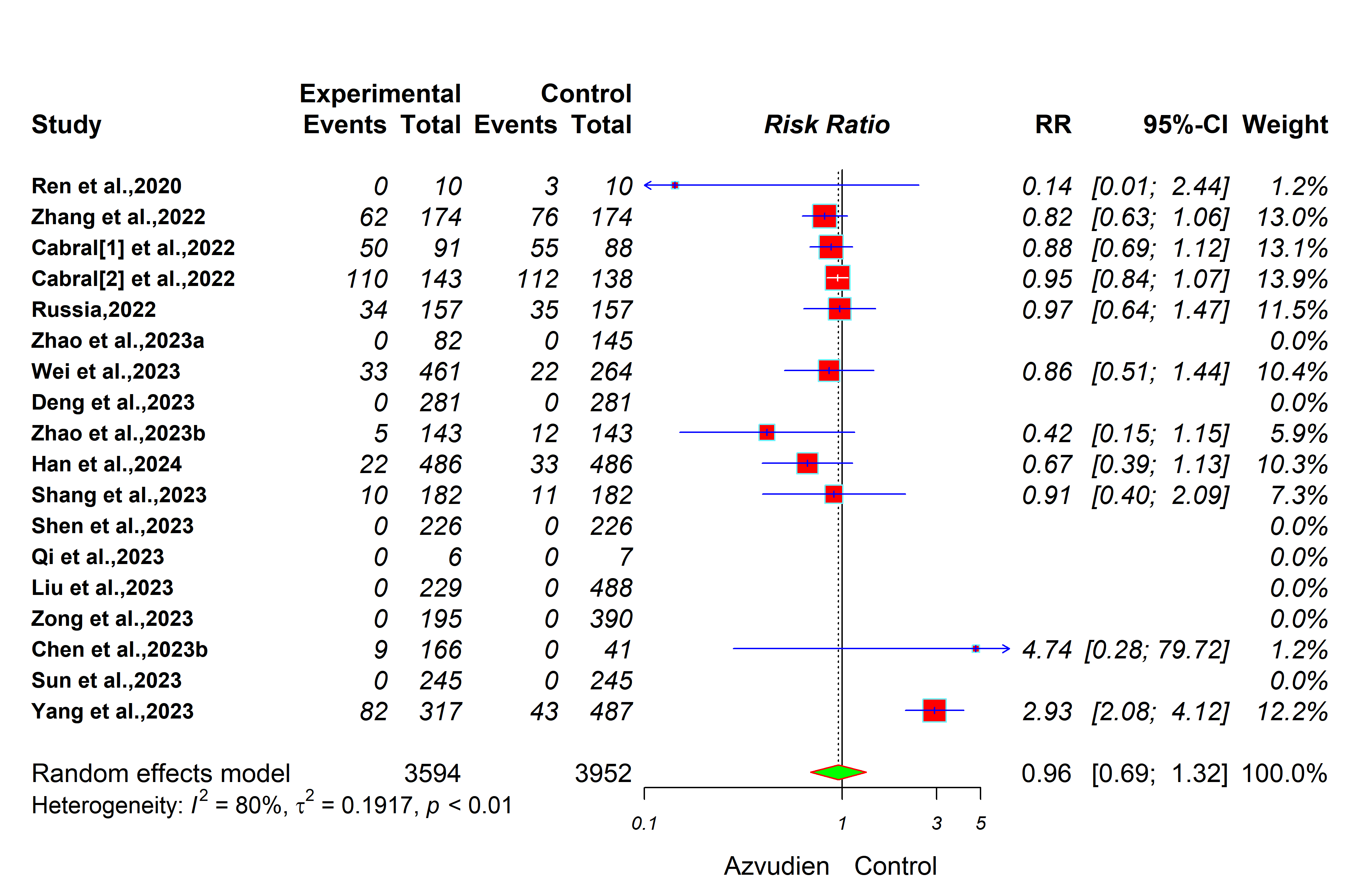


Figure S 68. Adverse events (Removing Chen et al.,2023a).


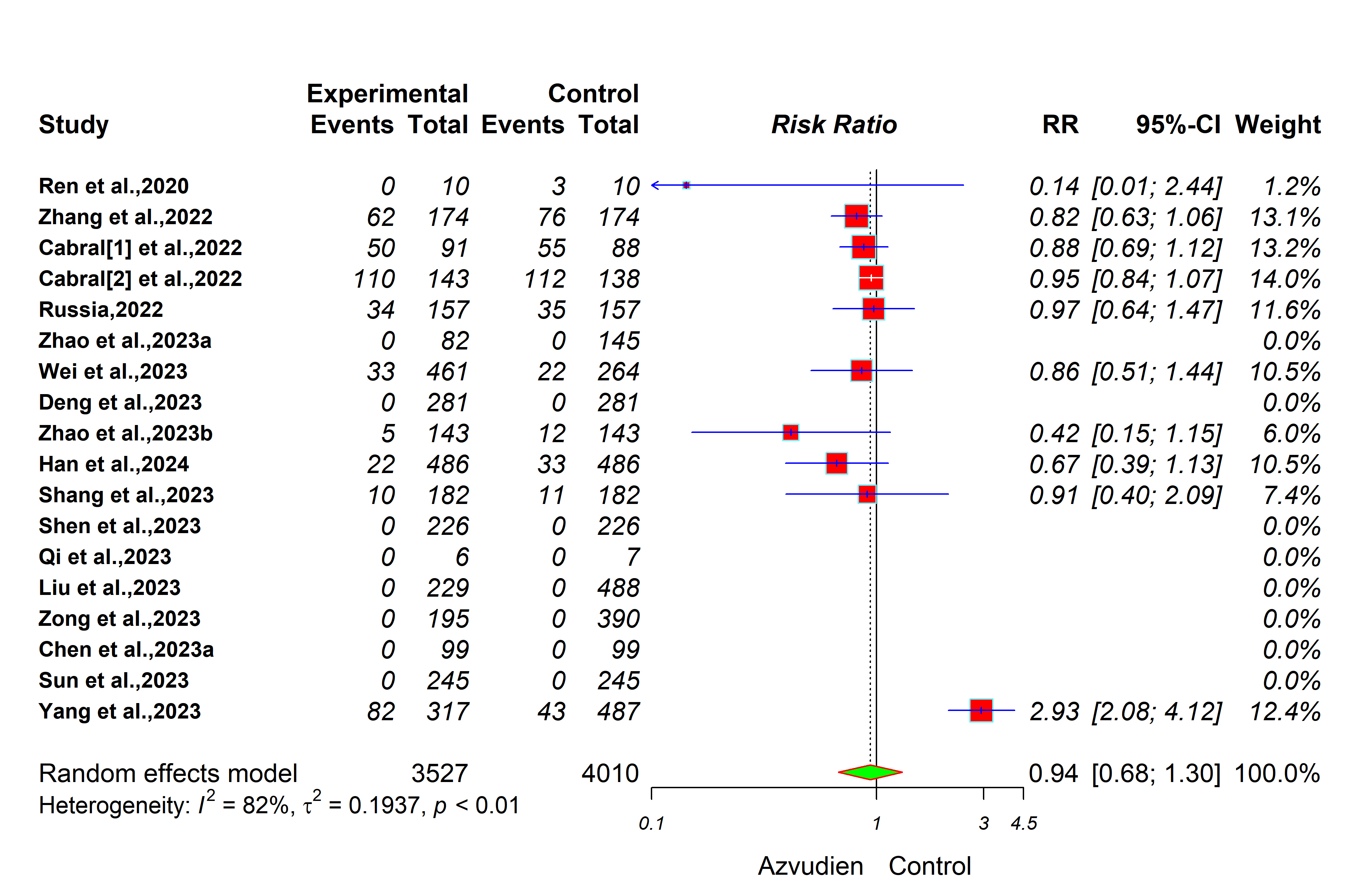


Figure S 69. Adverse events (Removing Chen et al.,2023b).


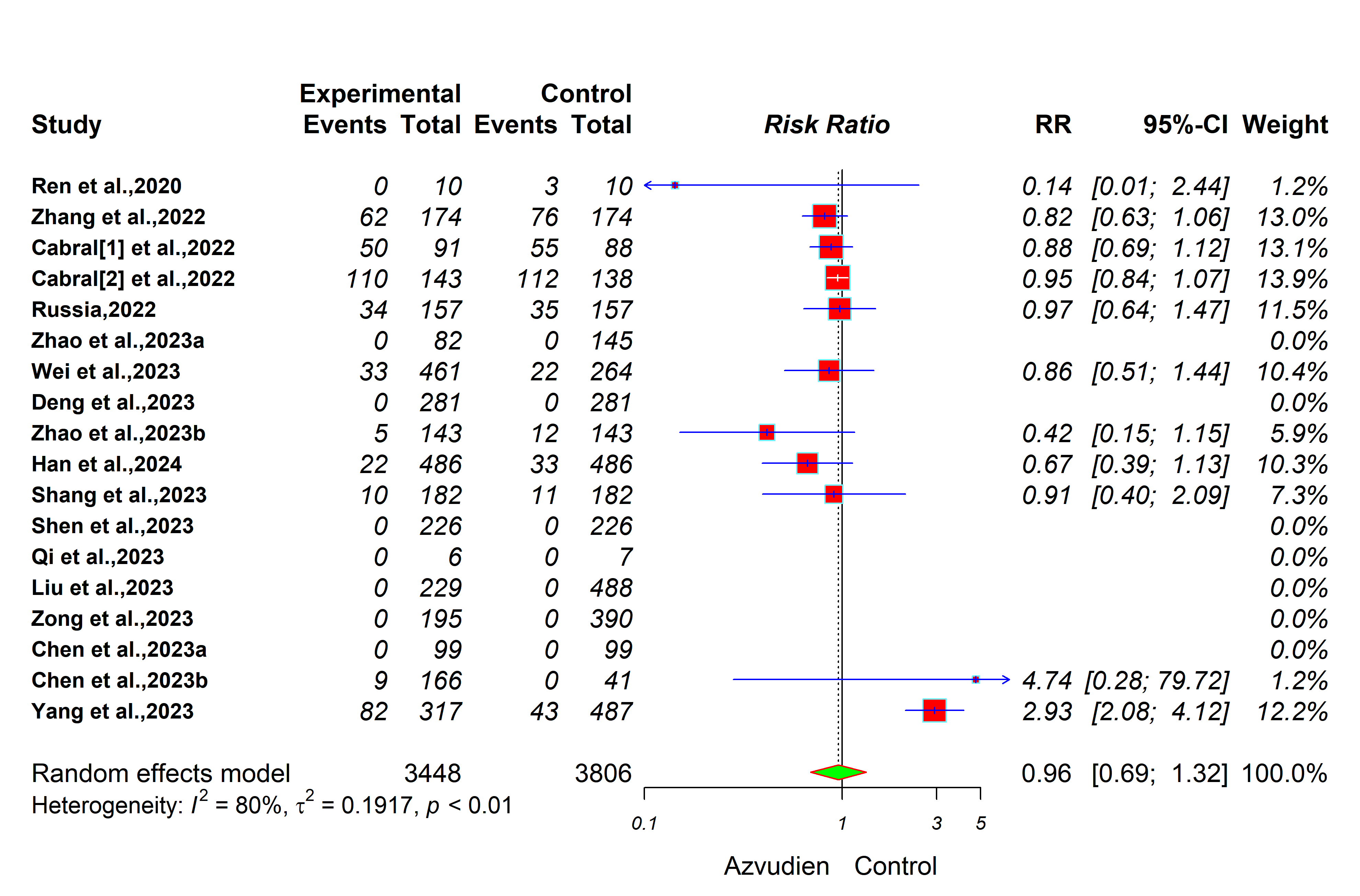


Figure S 70. Adverse events (Removing Sun et al.,2023).


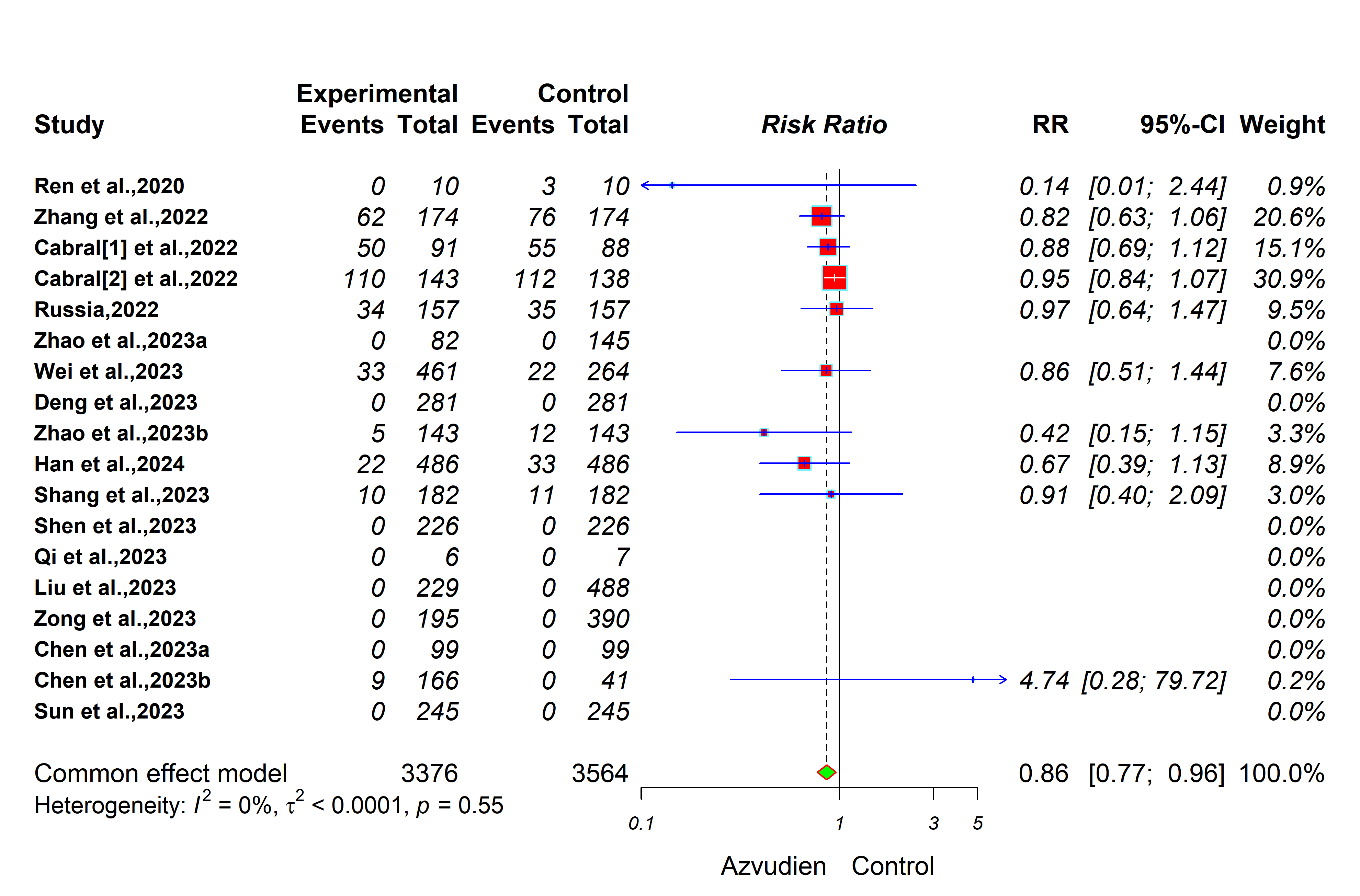


Figure S 71. Adverse events (Removing Yang et al.,2023).

# **Ⅱ. Supplementary table**

## Table S1. Quality assessment of included studies.

| **Study** | **Selection bias** | | **Performance bias** | **Detection bias** | **attrition bias** | **Reporting bias** | **Other bias** |
| --- | --- | --- | --- | --- | --- | --- | --- |
|  | **Random sequence generation** | **Allocation concealment** | **Blinding of subjects and experimenters** | **Blinding the outcome evaluator** | **Incomplete outcome data** | **Selective reporting of research outcome** |  |
| Ren et al.,2020  China^[21]^ | ↑^a^ | ↑^b^ | ↑^c^ | ↑^d^ | ↓ | ↓ | ? |
| Zhang et al.,2022  China^[22]^ | ↓ | ↓ | ↓ | ↓ | ↓ | ↓ | ? |
| Cabral[1] et al.,2022  Brazil^[23]^ | ↓ | ↓ | ↓ | ↓ | ↓ | ↑^e^ | ? |
| Cabral[2] et al.,2022  Brazil^[24]^ | ↓ | ↓ | ↓ | ↓ | ↓ | ↑^e^ | ? |
| 2022 Russia^[22]^ | ↓ | ↓ | ↓ | ↓ | ↓ | ↓ | ? |
| ↑: High risk of bias; ↓: Low risk of bias; ?: Unclear risk of bias  a: Small sample size RCTs may have difficulty ensuring adequate random sequence generation.  b: Small sample size RCTs may have difficulty ensuring adequate allocation concealment.  c: Small sample size RCTs may have difficulty implementing sufficient blinding.  d: Small sample size RCTs may have difficulty ensuring blinding of outcome assessors.  e: Only patients with mild or moderate COVID-19 were included in the study. | | | | | | | |

## Table S2**.** Quality assessment of included studies.

| **Study** | **Section** | | | | **Comparability** | **Outcome** | | |
| --- | --- | --- | --- | --- | --- | --- | --- | --- |
|  | **Representativeness of the exposed cohort** | **Selection of the non-exposed cohort** | **Ascertainment of the exposure** | **Outcome status at start of study** |  | **Assessment of the outcome** | **Length of follow-up** | **Adequacy of**  **follow-up** |
| Zhao et al.,  2023a China^[25]^ | + | + | + | + | + | + | -^a^ | + |
| Wei et al.,  2023 China^[26]^ | + | + | + | + | + | + | -^a^ | + |
| Deng et al.,  2023 China^[27]^ | + | + | + | + | ++ | + | + | + |
| Zhao et al.,  2023b China^[28]^ | + | + | + | + | ++ | + | + | + |
| Han et al.,  2024 China^[29]^ | + | + | + | + | + | + | + | + |
| Shang et al.,  2023 China^[30]^ | + | + | + | + | + | + | + | + |
| Shen et al.,  2023 China^[31]^ | + | + | + | + | ++ | + | + | + |
| Qi et al.,  2023 China^[32]^ | - | + | + | + | + | + | + | + |
| Liu et al.,  2023 China^[33]^ | + | + | + | + | ++ | + | + | + |
| Zong et al.,  2023 China^[34]^ | + | + | + | + | ++ | + | -^a^ | + |
| Chen et al.,  2023a China^[35]^ | + | + | + | + | + | + | -^a^ | + |
| Chen et al.,  2023b China^[36]^ | + | + | + | + | + | + | -^a^ | + |
| Sun et al.,  2023 China^[37]^ | + | + | + | + | + | + | + | + |
| Yang et al.,  2023 China^[38]^ | + | + | + | + | ++ | + | + | + |
| +: one point; ++: two point; -: zero point  a: No specific follow-up duration was defined. | | | | | | | | |

## Table S3. Severity of COVID-19 upon admission of patients.

| Study | Disease severity | Azvudine group | | Control group | |
| --- | --- | --- | --- | --- | --- |
|  |  | Events | Total | Events | Total |
| Zhao et al.,2023a^[25]^ | Mild | 35 | 82 | 55 | 145 |
|  | Common | 42 |  | 80 |  |
|  | Severe and critical | 5 |  | 10 |  |
| Wei et al.,2023^[26]^ | Moderate | 129 | 461 | 87 | 264 |
|  | Severe | 217 |  | 114 |  |
|  | Critical | 115 |  | 63 |  |
| Deng et al.,2023^[27]^ | Nonsevere | 101 | 281 | 103 | 281 |
|  | Severe | 180 |  | 178 |  |
| Zhao et al.,2023b^[28]^ | Mild to Moderate | 121 | 143 | 118 | 143 |
|  | Severe | 22 |  | 25 |  |
| Han et al.,2024^[29]^ | NR | NR | 486 | NR | 486 |
| Shang et al.,2023^[30]^ | NR | NR | 182 | NR | 182 |
| Shen et al.,2023^[31]^ | Nonsevere | 80 | 226 | 75 | 226 |
|  | Severe | 146 |  | 151 |  |
| Qi et al.,2023^[32]^ | Mild | 0 | 6 | 1 | 7 |
|  | Moderate | 1 |  | 0 |  |
|  | Severe | 2 |  | 3 |  |
|  | Critical | 3 |  | 3 |  |
| Liu et al.,2023^[33]^ | Mild | 27 | 229 | 36 | 488 |
|  | Moderate | 131 |  | 27 |  |
|  | Severe | 57 |  | 137 |  |
|  | Critical | 14 |  | 37 |  |
| Zong et al.,2023^[34]^ | Moderate | 94 | 195 | 205 | 390 |
|  | Severe | 79 |  | 153 |  |
|  | Critical | 22 |  | 32 |  |
| Chen et al.,2023a^[35]^ | Moderate | 9 | 99 | 9 | 99 |
|  | Severe | 58 |  | 57 |  |
|  | Grave | 32 |  | 33 |  |
| Chen et al.,2023b^[36]^ | Asymptomatic | 59 | 166 | 20 | 41 |
|  | Mild | 93 |  | 18 |  |
|  | Moderate | 14 |  | 3 |  |
| Sun et al.,2023^[37]^ | Nonsevere | 88 | 245 | 81 | 245 |
|  | Severe | 157 |  | 164 |  |
| Yang et al.,2023^[38]^ | Mild | 97 | 317 | 414 | 487 |
|  | Moderate | 220 |  | 73 |  |

NR: Not reported.

# Ⅲ. Supplementary figure

| A: Azvudine and Nirmatrelvir-Ritonavir |
| --- |
| 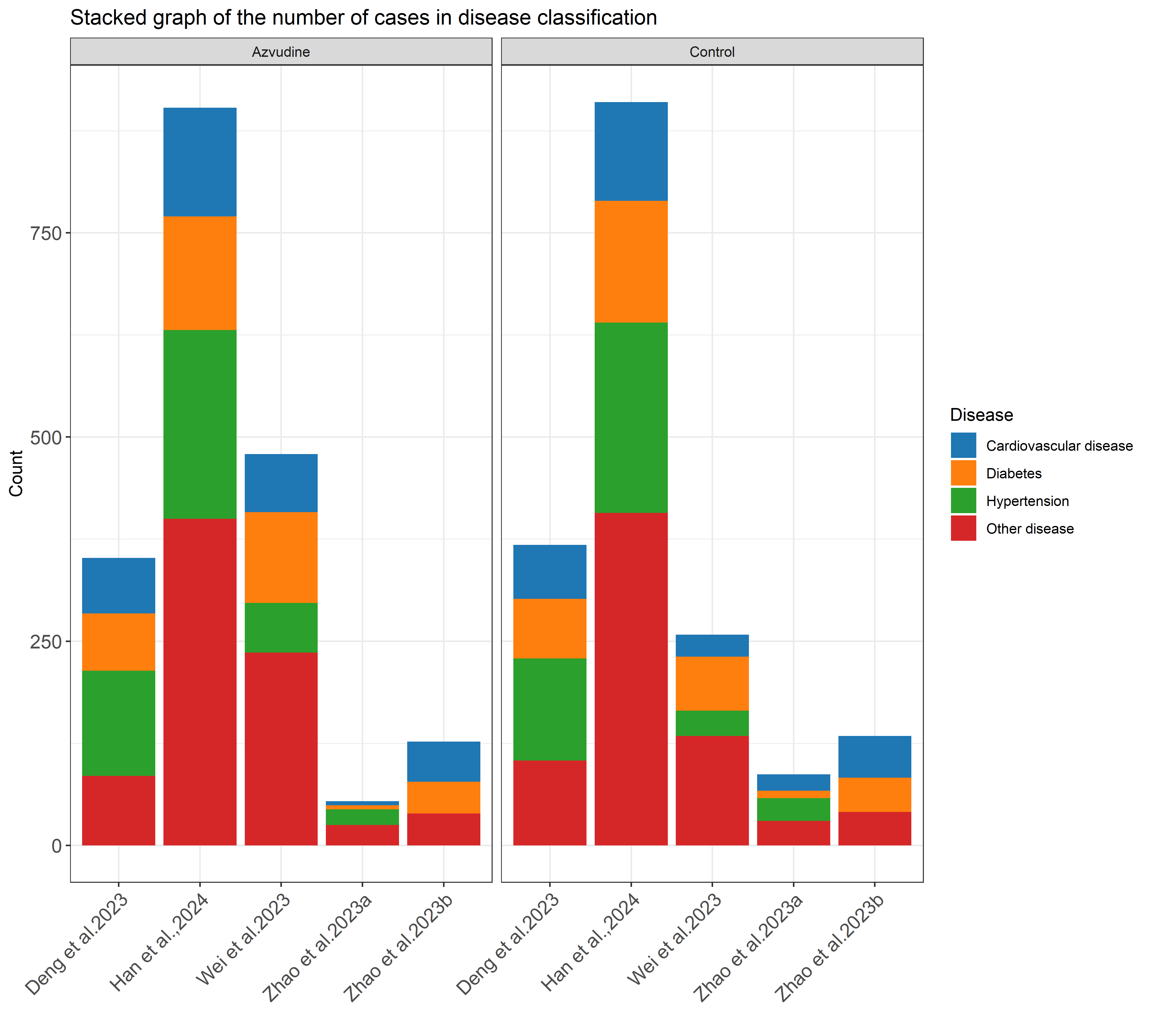 |
| B: Azvudine and control |
| 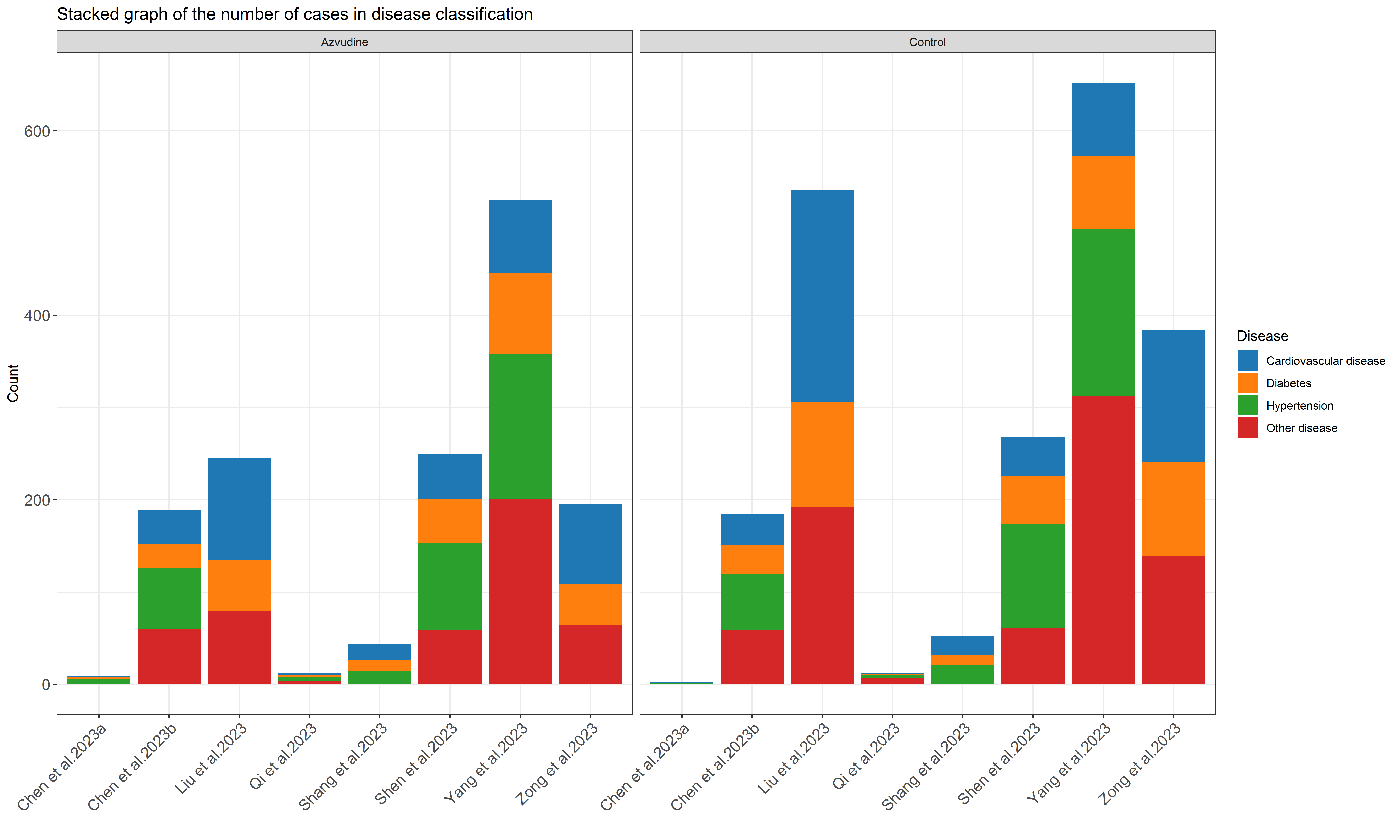 |

Figure S72. Stacked graph of basic disease in patients in the Azvudine group and control group.
